# Spatial transcriptomic analyses highlight distinct erythroid niches in mice and humans

**DOI:** 10.1038/s41588-026-02671-2

**Published:** 2026-07-02

**Authors:** Xu Han, Kehan Ren, Pan Wang, Honghao Bi, Ermin Li, Inci Aydemir, Amy Ji, Wenjie Cai, Laya Soleimanisardoo, Ching Man Wai, Matthew J. Schipma, Yijie Liu, Jeffery Goldstein, Madina Sukhanova, Jing Yang, Peng Ji

**Affiliations:** 1 Department of Pathology, Feinberg School of Medicine, Northwestern University, Chicago, IL USA; 2 Robert H. Lurie Comprehensive Cancer Center, Northwestern University, Chicago, IL USA; 3 Department of Biochemistry and Molecular Genetics, Northwestern University, Chicago, IL USA

**Keywords:** RNA sequencing, Transcriptomics

## Abstract

Erythroid cells require specialized microenvironments called erythroblastic islands (EBIs), niches comprising a central macrophage surrounded by developing erythroid precursors, to complete their maturation. Understanding EBI composition and function has been limited by two-dimensional in vitro models and the unclear composition of EBIs in human hematopoietic tissues. Using spatial transcriptomic mapping in mouse and human hematopoietic tissues during development and under stress conditions, we show that EBI architecture is unexpectedly species-specific. In mice, C1q-expressing macrophages serve as a hallmark of EBI central macrophages and mediate clearance of ejected erythroid nuclei. In humans, however, EBIs are characterized by macrophage-independent erythroid clusters in fetal liver and bone marrow, whose integrity depends critically on the adhesion molecule ICAM4. These human erythroid clusters are disrupted in myeloid diseases but can be restored with therapy. These findings redefine conventional models of erythroid niche biology and establish a framework for understanding niche dynamics across species.

## Abstract (web summary)

Spatial transcriptomic profiling of erythroblastic islands across tissues and developmental stages highlights distinct spatial architecture in humans compared to mice.

## Main

Mammalian terminal erythropoiesis occurs within specialized hematopoietic microenvironments called EBIs, structures comprising a central macrophage surrounded by developing erythroblasts^1–5^. Decades of research, primarily in murine models, have identified key surface molecules on EBI macrophages, including F4/80, EMP, Vcam-1, α_v_-integrin, CD163, CD169 and EpoR, that support erythropoiesis^6–9^. However, most studies rely on in vitro two-dimensional reconstitution systems that disrupt EBIs during isolation and fail to faithfully replicate their in vivo spatial organization. Moreover, the characterization and function of EBIs in humans remain inadequately explored.

Recent advances in imaging have revealed hematopoiesis at the spatial level, including the physical proximity of erythroblast clusters surrounding erythrocyte colony-forming units near the sinusoid^10^, and laser-capture microdissection-coupled sequencing has provided insights into the bone marrow niche^11^. In humans, single-cell transcriptomic and proteomic imaging studies have further elucidated cell–cell communications among hematopoietic and non-hematopoietic bone marrow cells^12^. Although collectively illuminating the native anatomy of hematopoietic niches, these approaches lack comprehensive, unbiased spatial transcriptomic coverage of hematopoiesis across development and species.

## Results

### C1q is a hallmark of mouse EBI macrophages in fetal erythropoiesis

**Fig. 1:**
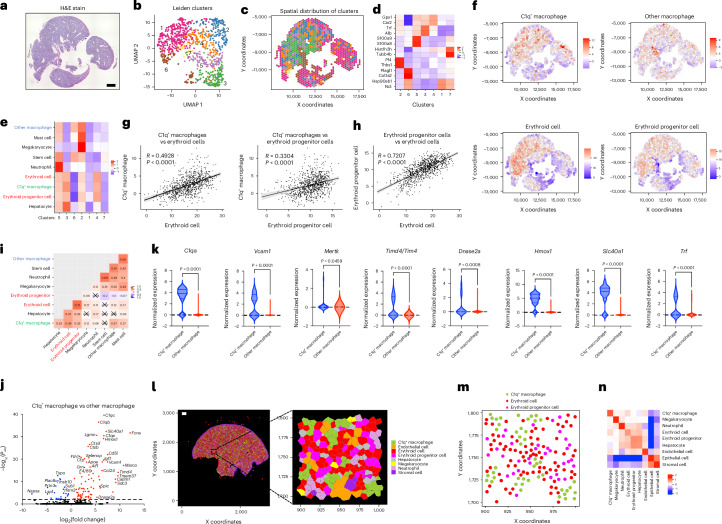
C1q is a hallmark of EBI macrophages in mouse fetal liver. **a**, H&E stain of formalin-fixed, paraffin-embedded E14.5 mouse fetal liver analyzed in the spatial transcriptomic analyses. Scale bar, 300 μm. **b**, Leiden clustering of the Visium spatial transcriptomic data in **a**. **c**, Spatial distributions of all clusters in **b**. **d**, Specific marker genes of all clusters in **b**. **e**, Cell type enrichment heatmap of the indicated cell types in different clusters. **f**, In situ distributions of indicated cell clusters. The scale indicates the enrichment of the indicated cell type within each dot. **g**, Correlation analyses between C1q^+^ macrophages and erythroid cells (left) and C1q^+^ macrophages and erythroid progenitors (right). **h**, Correlation analyses between erythroid cells and erythroid progenitors. For **g** and **h**, each dot represents one Visium capture spot. Statistical analysis was performed using Pearson correlation (two-sided). Shaded bands represent the 95% confidence interval of the linear regression. Pearson correlation coefficient (*R*) and *P* value are shown. **i**, Correlation *R* values between every two cell types. A positive *R*-value indicates a significant positive correlation, a negative *R*-value indicates a significant negative correlation and an *R*-value marked with ‘X’ indicates a non-significant correlation. **j**, Volcano plot showing genes differentially expressed in C1q^+^ macrophage and other macrophages. Data are from single-cell RNA-seq assays (GSM4647231). Red and blue dots demonstrate significantly upregulated or downregulated genes in C1q^+^ macrophages compared to other macrophages, respectively. **k**, Expression of the indicated genes in C1q^+^ macrophages and other macrophages. For **j** and **k**, Welch’s *t*-test (two-tailed) was used. **l**, Xenium spatial transcriptomic in situ cell types mapping in E14.5 mouse fetal liver with indicated cell types. Scale bar, 100 μm. **m**, In situ mapping of C1q^+^ macrophage, erythroid cell and erythroid progenitor cell in **l**, illustrated as colored circles. **n**, A heatmap showing interactions between two cell types from the Xenium data. Source data

**Extended Data Fig. 1:**
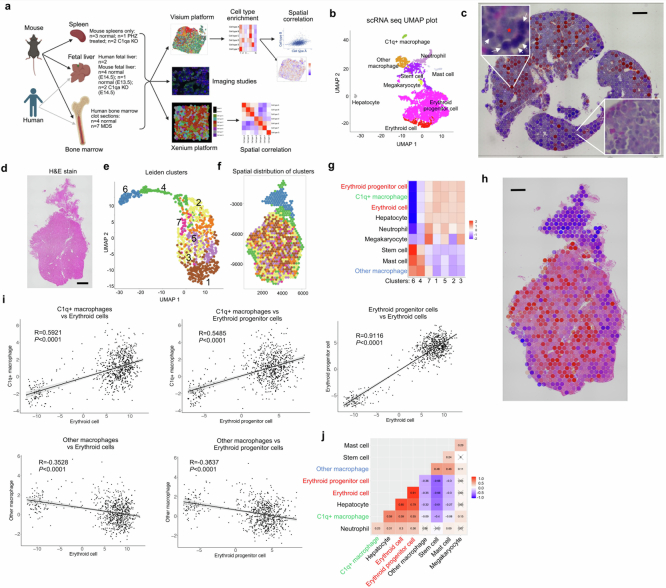
C1q is a hallmark of EBI macrophages in mouse fetal liver. **a**, Schematic strategy of the sequencing studies. **b**, UMAP plot showing the indicated cell populations after reanalyzing single-cell RNA seq data from GSM4647231. **c**, Overlap of H&E stain with Visium spatial distribution clusters of C1q+ macrophages. Representative high-power H&E images of the C1q+ macrophage-enriched red clusters and less-enriched blue clusters are illustrated in the inserted panels. White arrows indicate erythroblasts surrounding a central macrophage (red star). Scale bar: 200 μm. **d**, H&E stain of formalin-fixed paraffin-embedded E14.5 mouse fetal liver analyzed in the following spatial transcriptomic analyses. Scale bar: 300 μm. **e**, Leiden clustering of the Visium spatial transcriptomic data in d. **f**, Spatial distributions of all clusters in e. **g**, Cell type enrichment heatmap of the indicated cell types in different clusters. **h**, Overlap of H&E stain with Visium spatial distribution clusters of C1q+ macrophages. Scale bar: 200 μm. **i**, Correlation analyses between the indicated two cell clusters. Each dot represents one Visium capture spot. Statistical analysis was performed using Pearson correlation (two-sided). Shaded bands represent the 95% confidence interval of the linear regression. Pearson correlation coefficient (R) and p-value are shown. **j**, Correlation R values between every two cell types. A positive R-value indicates a significant positive correlation, a negative R-value indicates a significant negative correlation, and an R-value marked with “X” indicates a non-significant correlation. Source data

To comprehensively investigate the spatial relationships among diverse cell types in mouse and human hematopoietic systems during development in an unbiased approach, we applied a spatial transcriptomics assay based on the 10x Genomics Visium platform (Visium V1) in different hematopoietic organs, including fetal liver, bone marrow and adult spleen (Extended Data Fig. 1a). Although the Visium platform has a limited resolution—each barcoded sequencing unit contains approximately ten to twenty hematopoietic cells (except megakaryocytes, which could occupy a whole unit)—it is ideal for characterizing EBIs, given that each unit would fit a single EBI in which the composition of the cell types can be deciphered. We first analyzed mouse fetal liver, the major organ of fetal erythropoiesis, at E14.5, when erythroblasts are actively proliferating^13,14^ (Fig. 1a). Dimension reduction and clustering analyses identified seven clusters representing major fetal liver cell populations (Fig. 1b). Spatial distribution plotting further reveals distinctive spatial localizations of these clusters in different lobes of the mouse fetal liver, with cluster 1 mainly located in two small lobes and cluster 3 in the largest lobe (Fig. 1c). These unique distributions suggest compartmentalized hematopoiesis during mouse fetal development. Each Visium spot captures compiled reads from all enclosed cells, with cellular identities determined by marker gene expression. In this respect, erythroid markers *Gpx1*, *Car2* and *Trf* are highly expressed in clusters 3 and 4, while megakaryocytic markers (*Pf4*, *Thbs1*) and myeloid markers (*S100a8*, *S100a9*) predominate in clusters 2 and 5, respectively (Fig. 1d).

To resolve the low-resolution issue in Visium, we performed a cell type enrichment assay using Visium cluster markers and a published single-cell RNA sequencing (scRNA-seq) dataset from E14.5 mouse fetal liver^15^. The uniform manifold approximation and projection (UMAP) plot of the scRNA-seq data shows mainly nine cell types (Extended Data Fig. 1b and Fig. 1e). Combined analyses of the scRNA-seq and Visium data reveal that each Visium cluster is a mixture of several cell types. Importantly, these two datasets show consistent marker gene expression patterns, with erythroid cells and erythroid progenitors predominantly found in clusters 3 and 4, megakaryocytes in cluster 2 and myeloid or granulocytic cells in cluster 5 (Fig. 1e and Supplementary Table 1). Notably, erythroid and erythroid progenitor cells in clusters 3 and 4 are highly associated with the gene expression profiles of C1q^+^ macrophages, but not other macrophages. This indicates that these cell types are spatially adjacent. Similarly, megakaryocytes are located close to mast cells and C1q^−^ other types of macrophages (Fig. 1e).

We confirmed the spatial proximity of these cell types using a quantitative correlation analysis between the two cell types after extracting the relative expression scores for each cell type in each cluster. We found a positive correlation between C1q^+^ macrophages and both erythroid cells and erythroid progenitor cells (Fig. 1f,g,i and Extended Data Fig. 1c). This indicates a strong spatial correlation between C1q^+^ macrophages and the erythroid lineage, implying that C1q^+^ macrophages may be particularly inclined to function as EBI macrophages compared to other macrophage types. Notably, we also observed a significantly stronger positive correlation between erythroid cells and their progenitors, highlighting a more intimate physical association among cells within the erythroid lineage (Fig. 1e,f,h,i). We repeated the same Visium transcriptomic study using an E14.5 fetal liver from a different wild-type mouse and obtained the same results (Extended Data Fig. 1d–j). Similar findings were also observed in a wild-type E13.5 mouse fetal liver (Supplementary Fig. 1). Notably, the positive spatial association between C1q^+^ macrophages and erythroid cells at E13.5 was weaker than at E14.5, suggesting that the organization of C1q^+^ macrophage-centered EBIs strengthens as fetal erythropoiesis progresses.

**Extended Data Fig. 2:**
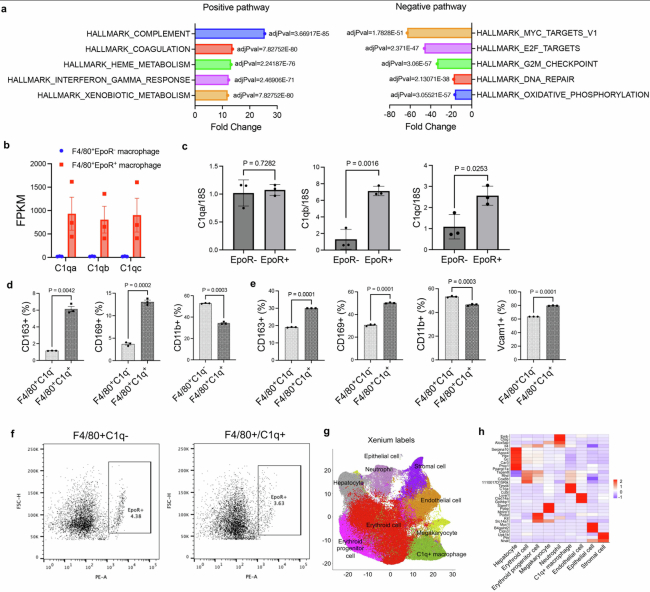
C1q+ macrophages express classic EBI macrophage markers. **a**, The differential signaling pathways between C1q+ macrophages and other macrophages were analyzed in R using the E14.5 fetal liver single cell RNA sequencing dataset GSM4647231. Top 5 significantly upregulated (positive) and downregulated (negative) pathways in C1q+ macrophages compared to other macrophages were shown based on the fold change; n = 1. **b**, Relative mRNA expression levels of indicated C1q genes in the indicated macrophages. Data is from a published report (ref. ^9^). Data represent mean ± SD from n = 3 independent biological replicates in each group. The comparison was evaluated using a Student’s t-test. **c**, Quantitative PCR analysis of C1qa, C1qb, and C1qc expression in the bone marrow F4/80+EpoR- (EpoR-) and F4/80+EpoR+ (EpoR+) macrophages sorted from EpoR-tdTomato-Cre mice. Gene expression was normalized to 18S. Data represent mean ± SD from n = 3 independent biological replicates in each group. The comparisons were evaluated using Student’s t-tests. **d-e**, Quantification of flow cytometry analyses of indicated surface proteins in mouse E14.5 fetal liver (d) or bone marrow (e) F4/80+C1q- and F4/80+C1q+ macrophages. Data represent mean ± SD from n = 3 independent biological replicates in each group. The comparisons were evaluated using Student’s t-tests. **f**, Flow cytometry analysis of EpoR levels of the indicated bone marrow macrophages. The percentage of EpoR+ cells is presented. **g**, UMAP plot derived from Xenium subcellular spatial transcriptomic data showing different cell types in mouse E14.5 fetal liver. **h**, Specific marker genes of all cell types in g. Source data

Next, we performed a pathway enrichment assay comparing the C1q^+^ and other macrophages from the scRNA-seq data^15^. Notably, in addition to the expected complement and coagulation hallmarks, the heme metabolism pathway was also significantly enriched in C1q^+^ macrophages, indicating the roles for C1q^+^ macrophages in supporting erythropoiesis, especially iron supply and recycling (Extended Data Fig. 2a). A volcano plot validates the highly expressed C1q family genes in the C1q^+^ macrophages (Fig. 1j). In addition, genes known to be involved in EBI macrophages in supporting erythropoiesis also show significantly higher expression levels in C1q^+^ macrophages than in other macrophages. These genes include *Vcam1* (ref. ^9^), pivotal in cell adhesion; *MerTK*^16^, *Timd4* (ref. ^17^) and *Dnase2a*^18^, crucial for the engulfment of erythroid extruded nuclei; and *Hmox1* (ref. ^19^), *Slc40a1* (ref. ^20^) and *Trf*, essential for iron metabolism. Furthermore, several transcription factors, including *Spic*^21^, *Nr1h3* and *Maf*^21^, and two growth factors, *Igf1* (ref. ^22^) and *Il18*, reported to have roles in nourishing erythroblast development, were also significantly increased in C1q^+^ macrophages compared with other macrophages (Fig. 1j,k). Previous reports showed that the erythropoietin receptor (EpoR) is expressed in EBI macrophages^9,23^. We found that C1q family genes are highly expressed in F4/80^+^EpoR^+^ EBI macrophages (Extended Data Fig. 2b,c). C1q^+^ macrophages also express high levels of CD169 and CD163 in both fetal liver and bone marrow (Extended Data Fig. 2d,e). The EpoR levels were not significantly different between F4/80^+^C1q^+^ and F4/80^+^C1q^−^ macrophages, suggesting that although C1q^+^ macrophages may overlap with EpoR^+^ macrophages, they are not exclusively defined by EpoR expression (Extended Data Fig. 2f).

**Extended Data Fig. 3:**
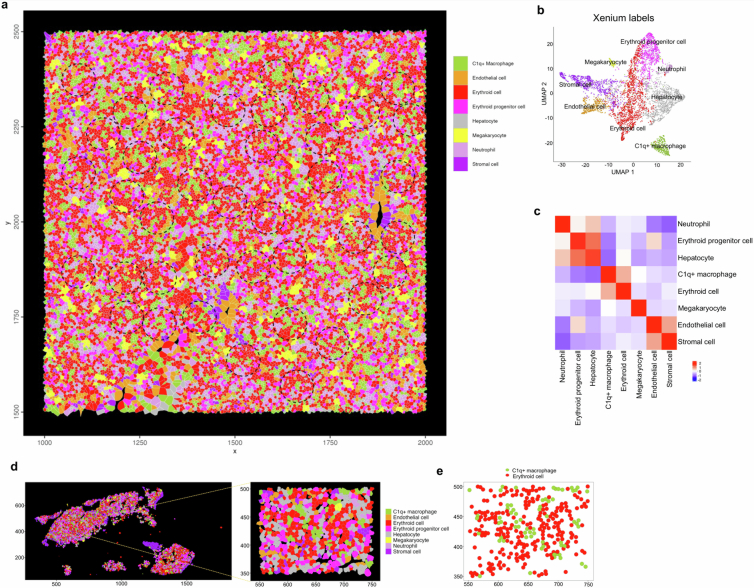
Xenium spatial transcriptomic analyses of mouse fetal liver. **a**, Xenium spatial transcriptomic in situ cell types mapping in E14.5 mouse fetal liver with indicated cell types for the calculation of the distances between neighboring C1q+ macrophage-centered EBIs. The circled region represents an EBI structure. The distance between neighboring EBIs was calculated by measuring the pixel distance between the central macrophages of neighboring EBIs in ImageJ and converting it into physical distance based on the actual size of the sample on the Xenium slide. **b**, UMAP plot derived from Xenium 5 K high-plex platform subcellular spatial transcriptomic showing different cell types in E13.5 wild-type mouse fetal liver. **c**, A heatmap showing interactions between two cell types from the Xenium data in b. **d**, High-plex Xenium spatial transcriptomic in situ cell types mapping in E13.5 mouse fetal liver with indicated cell types. **e**, In situ mapping of C1q+ macrophage and erythroid cell in d, illustrated as colored circles.

To further illustrate the spatial relationship between C1q^+^ macrophages and erythroid cells at single-cell resolution, we used the Xenium platform, which can capture hundreds of RNAs per cell. In addition to probes targeting commonly expressed genes, we designed 98 probes to capture highly expressed genes across different cell lineages, based on scRNA-seq data (Supplementary Table 2). Dimension reduction and marker-based annotation identified nine cell types in E14.5 mouse fetal liver, including C1q^+^ macrophages, erythroid cells and erythroid progenitors (Extended Data Fig. 2g,h). Cell-distance-based interaction analysis confirmed strong C1q^+^ macrophage–erythroid associations, consistent with C1q^+^ macrophages serving as EBI macrophages, alongside strong erythroid–progenitor interactions (Fig. 1l–n). The average distance between neighboring EBIs was 108.35 ± 28.11 µm (mean ± s.d.), with an average EBI diameter of ~80 µm containing 25.1 ± 3.5 (mean ± s.d.) erythroid cells per C1q^+^ EBI (Extended Data Fig. 3a). High-plex Xenium in situ hybridization on E13.5 fetal liver confirmed similar spatial distributions (Extended Data Fig. 3b–e and Supplementary Table 3).

### C1q^+^ marks EBI macrophages in mouse bone marrow

**Fig. 2:**
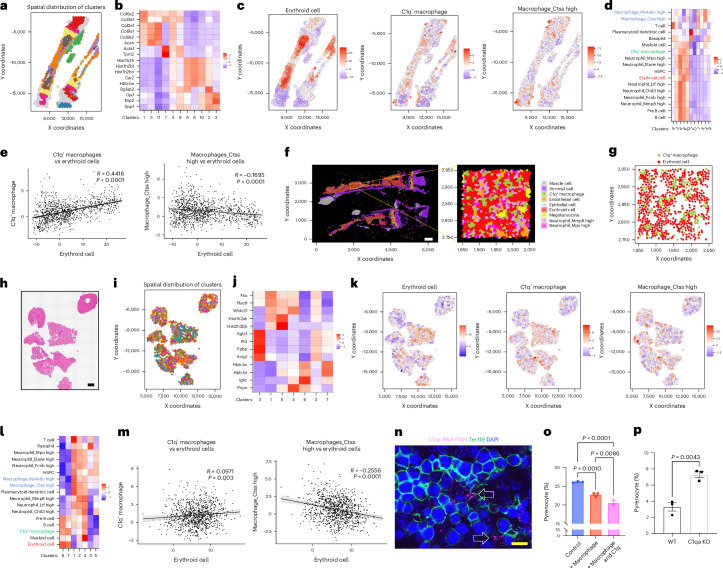
C1q^+^ macrophages are the EBI macrophages in mouse bone marrow. **a**, Spatial distributions of cell clusters from the Visium spatial transcriptomic data of day 8 postnatal mouse bone marrow. **b**, Specific marker genes of the 11 clusters shown in **a**. **c**, In situ mapping of the erythroid cell (left), C1q^+^ macrophage (middle) and macrophage_Ctss high (right). Scale indicates the enrichment of the indicated cell type within each dot. **d**, Cell type enrichment heatmap of the indicated cell types in different clusters. **e**, Correlation between C1q^+^ macrophages and erythroid cells (left), and correlation between other macrophages (macrophage_Ctss high) and erythroid cells (right). Each dot represents one Visium capture spot. Statistical analysis was performed using Pearson correlation (two-sided). Shaded bands represent the 95% confidence interval of the linear regression. Pearson correlation coefficient (*R*) and *P* value are shown. **f**, Xenium spatial transcriptomics showing in situ mapping of indicated cell types in mouse bone marrow. Representative genes and their cellular localization are illustrated as colored dots. Scale bar, 300 μm. **g**, C1q^+^ macrophages and erythroid cells are specifically illustrated as colored circles. **h**, H&E stains of formalin-fixed, paraffin-embedded bone marrow sections prepared from cryosectioned femur from a 20-month-old male mouse. Scale bar, 250 μm. **i**, Spatial distributions of cell clusters from the Visium spatial transcriptomic data shown in **h**. **j**, Specific marker genes of the seven clusters shown in **i**. **k**, In situ mapping of the erythroid cells (left), C1q^+^ macrophages (middle) and macrophage_Ctss high (right). **l**, Cell type enrichment heatmap of the indicated cell types in different clusters. **m**, Correlation between C1q^+^ macrophage and erythroid cell (left), and correlation between other macrophages (macrophage_Ctss high) and erythroid cells (right). Each dot represents one Visium capture spot. Statistical analysis was performed using Pearson correlation (two-sided). Shaded bands represent the 95% confidence interval of the linear regression. Pearson correlation coefficient (*R*) and *P* value are shown. **n**, Representative images of combined C1qa RNA FISH with immunofluorescence stain of Ter119 on E14.5 mouse fetal liver. Arrows indicate C1qa^+^ macrophages. Scale bar, 10 μm. The data represent three independent biological replicates. **o**, Quantification of flow cytometry detected pyrenocytes in the 48 h cultured erythroblasts in the presence or absence of macrophages and/or recombinant C1q. Data represent mean values from *n* = 3 independent biological replicates in each group; error bars, s.d. The comparison was evaluated using a one-way ANOVA. **p**, Quantification of flow cytometry detected pyrenocytes in the bone marrow of the indicated mice at 1 year of age. Data represent mean values from *n* = 3 independent biological replicates in each group; error bars, s.d. The comparison was evaluated using a Student’s *t*-test. WT, wild type; KO, knockout. Source data

**Extended Data Fig. 4:**
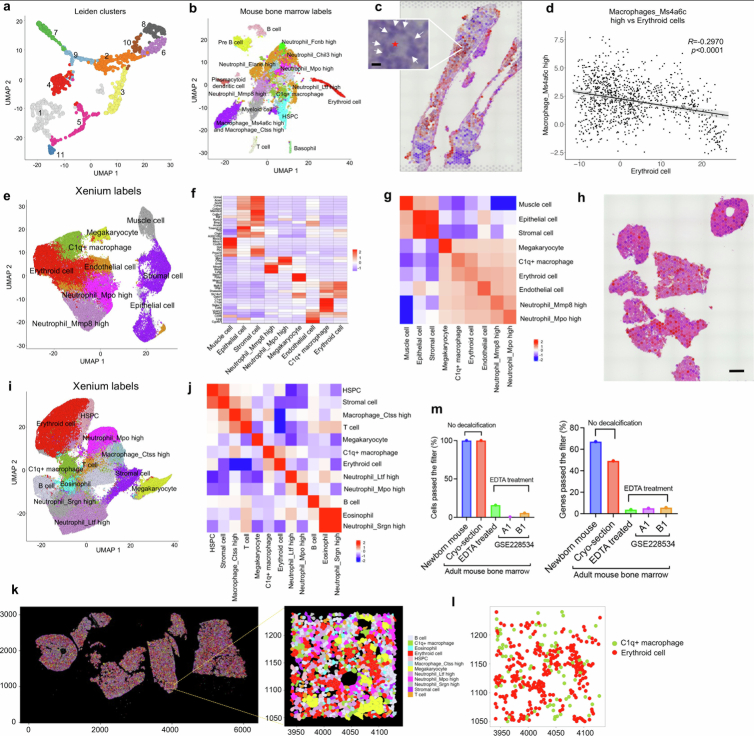
C1q+ macrophages are the EBI macrophages in mouse bone marrow. **a**, Leiden clustering of the Visium spatial transcriptomic data of day 8 postnatal mouse bone marrow. **b**, UMAP plot showing the indicated cell populations after reanalyzing single-cell RNA seq data from the combination of GSM2906396, GSM2906399, and GSM2906400. **c**, Overlap of H&E stain with Visium spatial distribution clusters of C1q+ macrophages. Representative high-power H&E images of the C1q+ macrophage-enriched red clusters are illustrated in the inserted panels. White arrows indicate erythroblasts surrounding a central macrophage (red star). Scale bar: 10 μm. **d**, Correlation analysis between other macrophages (macrophage_Ms4a6c high) and erythroid cells. Each dot represents one Visium capture spot. Statistical analysis was performed using Pearson correlation (two-sided). Shaded bands represent the 95% confidence interval of the linear regression. Pearson correlation coefficient (R) and p-value are shown. **e**, UMAP plot derived from Xenium subcellular spatial transcriptomic data showing different cell types in day 8 postnatal mouse bone marrow. **f**, Specific marker genes of all cell types in e. **g**, A heatmap showing interactions between two cell types from the Xenium data. **h**, Overlap of H&E stain with Visium spatial distribution clusters of C1q+ macrophages. **i**, High-plex Xenium spatial transcriptomic analyses of adult mouse bone marrow. UMAP plot derived from Xenium 5 K high-plex platform subcellular spatial transcriptomic showing different cell types in adult mouse bone marrow. **j**, A heatmap showing interactions between two cell types from the Xenium data in i. **k**, Xenium spatial transcriptomic in situ cell types mapping of adult mouse bone marrow with indicated cell types. **l**, In situ mapping of C1q+ macrophage and erythroid cell in k, illustrated as colored circles. **m**, Quantitative analyses of cells (left) and genes (right) that passed the filters for Visium spatial transcriptomic studies using the indicated decalcification methods. A1 and B1 bone marrow blocks from a published dataset (GSE228534) are also included for comparison. n = 1 in each group. Source data

To determine whether C1q is a hallmark of EBI macrophages in mouse bone marrow, we applied spatial transcriptomics on day 8 postnatal mouse femurs to avoid decalcification-induced RNA damage. We identified 11 clusters, with clusters 6, 8 and 10 expressing erythroid-specific genes such as *Car2* and *Hbb-bs* (Fig. 2a,b and Extended Data Fig. 4a). We then performed a cell type enrichment assay by incorporating cell type signatures derived from a reported scRNA-seq database of mouse bone marrow (Extended Data Fig. 4b)^15^ and the gene expression profiles from the Visium spatial transcriptomic data. The heatmap and spatial distribution plots revealed co-localizations between erythroid cells and C1q^+^ macrophages, but not other macrophages (Fig. 2c,d and Extended Data Fig. 4c). Notably, C1q^+^ macrophages also show strong proximity to myeloid cells and neutrophils (Fig. 2d), consistent with a recent report that mouse bone marrow EBIs support granulopoiesis parallel to terminal erythropoiesis^24^. Further analysis confirmed a positive correlation between C1q^+^ macrophages and erythroid cells, while a negative correlation was observed between the other macrophage types and erythroid cells (Fig. 2e and Extended Data Fig. 4d). We also applied the Xenium platform in the same mouse bone marrow sample by implementing the same set of probes as in the fetal liver. Dimension reduction and Leiden clustering revealed nine cell types, including C1q^+^ macrophages and erythroid cells (Fig. 2f and Extended Data Fig. 4e). Consistent with the Visium results, the spatial distributions of different cell types further demonstrated the existence of EBI structure formed by C1q^+^ macrophage and erythroid cells, as well as macrophage-independent erythroid clusters (Fig. 2f,g and Extended Data Fig. 4f,g).

To determine the spatial transcriptomic profiles of adult bone marrow, we modified a reported protocol using cryosectioning without decalcification^25^. Differential thermal expansion between bone and marrow enabled clean separation while preserving marrow integrity. This was particularly convenient in older adult mice, whose bone fragility further facilitated isolation. We performed the same Visium spatial transcriptomic analyses of the cryosectioned marrow and found a consistent positive correlation between C1q^+^ macrophages and erythroid cells (Fig. 2h–m and Extended Data Fig. 4h). Notably, the correlation becomes weaker than in the newborn bone marrow, indicating an aging-associated change in mouse EBI structure. The same spatial relationships were confirmed by a high-plex Xenium analysis (Extended Data Fig. 4i–l). Previous reports applied ETDA-based decalcification methods to analyze adult mineralized bone tissues for spatial transcriptomic studies^26–28^. We compared available EDTA methods with our cryosectioning protocol and found that although all passed initial quality control, cryosectioned tissue exhibited superior quality (Extended Data Fig. 4m). Overall, these data support the conclusion that C1q is a hallmark of the EBI central macrophages in mouse hematopoietic tissues.

### C1q is involved in the engulfment of pyrenocytes

**Extended Data Fig. 5:**
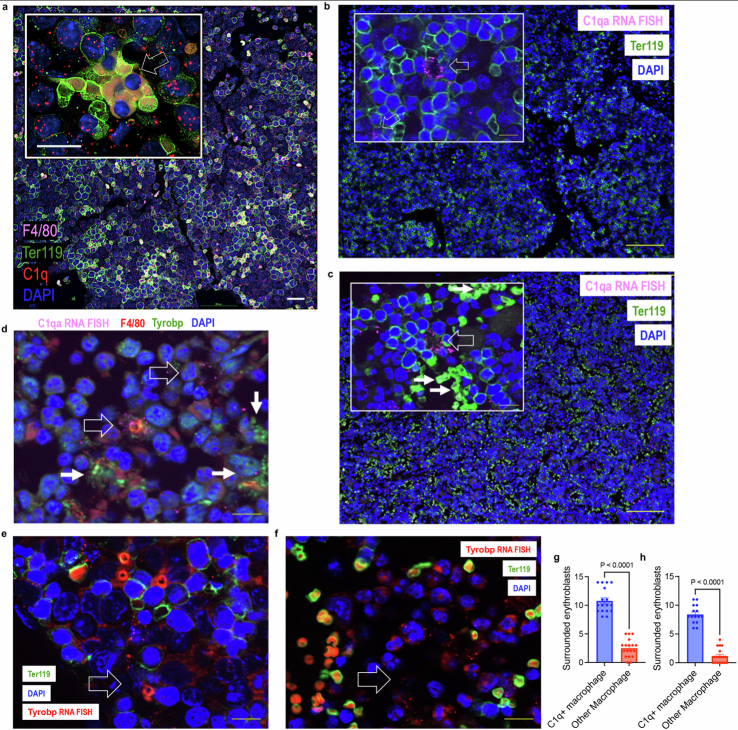
C1q+ macrophage and erythroid cells form EBIs in mouse fetal liver and bone marrow. **a**, Representative images of immunofluorescence stain of F4/80, Ter119, and C1q on E14.5 mouse fetal liver. The inserted figures are magnified parts. The arrow indicates a macrophage engulfing a pyrenocyte. Scale bar: 25 µm. Scale bar in magnified part: 10 µm. **b**, Representative images of combined C1qa RNA FISH with immunofluorescence stain of Ter119 on E14.5 mouse fetal liver. The inserted figures are magnified parts. The arrow indicates a C1qa+ macrophage. Scale bar: 100 µm. Scale bar in magnified part: 10 µm. **c**, Representative images of combined C1qa RNA FISH with immunofluorescence stain of Ter119 on adult mouse bone marrow. The inserted figures are magnified parts. The open arrow indicates a C1qa+ macrophage. The solid arrows indicate reticulocytes. Scale bar: 100 µm. Scale bar in magnified part: 10 µm. **d**, Representative images of combined C1qa RNA FISH with immunofluorescence stain of F4/80 and Tyrobp on adult mouse bone marrow. Open and solid arrows indicate C1qa+ macrophages and other macrophages (Tyrobp high), respectively. Scale bar: 10 µm. **e-f**. Representative images of combined Tyrobp RNA FISH with immunofluorescence stain of Ter119 on E14.5 mouse fetal liver (e) or adult mouse bone marrow (f). The arrows indicate Tyrobp+ macrophages. Scale bars: 10 µm. **g-h**, Quantification of surrounding erythroblast numbers of the indicated macrophages in mouse E14.5 fetal liver (g, C1q+ macrophages: n = 17; Other macrophages: n = 18) and adult bone marrow (h, C1q+ macrophages: n = 15; Other macrophages: n = 18). Data represent mean ± SD. The comparisons were evaluated using Student’s t-tests. Source data

To validate spatial transcriptomic findings, we performed multiplexed immunofluorescence on E14.5 mouse fetal liver. As expected, F4/80 highlighted scattered EBI macrophages surrounded by erythroblasts, with occasional pyrenocytes (extruded nuclei) being engulfed by the central macrophage (Extended Data Fig. 5a). Notably, many erythroblasts formed macrophage-independent clusters, consistent with spatial transcriptomic data. Unlike F4/80, C1q showed a diffuse dotted distribution throughout the fetal liver, reflecting its extracellular secretion^29^. This further underscores the value of spatial transcriptomics in identifying the original cell sources of specific gene expression. To validate C1q^+^ macrophages as the sources of C1q, ViewRNA fluorescent staining of E14.5 mouse fetal liver detected C1qa RNA in macrophages surrounded by Ter119^+^ erythroblasts (Fig. 2n and Extended Data Fig. 5b), with the same structures identified in adult bone marrow (Extended Data Fig. 5c). Approximately 86.67 ± 18.86% (mean ± s.d.) of C1q^+^ EBI macrophages are directly attached by >5 erythroblasts. C1qa^+^ macrophages also co-express F4/80 (Extended Data Fig. 5d). By contrast, Tyrobp-high other macrophages in both fetal liver and adult bone marrow were rarely associated with surrounding erythroblasts (Extended Data Fig. 5e–h).

**Extended Data Fig. 6:**
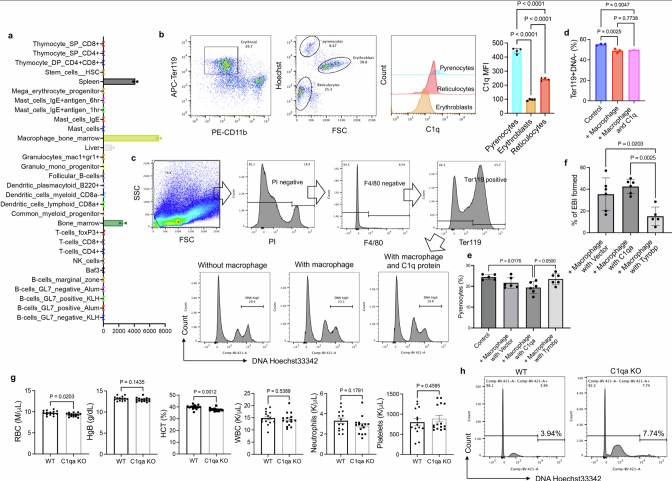
C1q is highly expressed in macrophages and promotes macrophage-mediated pyrenocyte engulfment. **a**, Relative mRNA expression levels of C1qa in the indicated tissues or cell types in mice. The data is from biogps.org. **b**, Wild-type (WT) mice were treated with 40 mg/kg phenylhydrazine (PHZ) twice per day for two days. Flow cytometry analysis of C1q levels in the bone marrow reticulocyte (FSC^low^DNA^−^), erythroblast (FSC^high^DNA^mid^), and pyrenocyte (FSC^low^DNA^high^) after PHZ treatment. The gating strategy is illustrated on the left. Data represent mean ± SD from n = 4 independent biological replicates in each group. The comparison was evaluated using a One-Way ANOVA. **c**, Bone marrow lineage-negative cells were co-cultured with bone marrow-derived macrophages (BMDMs) in the Epo-containing medium for 48 h. 25 µg/mL C1q was added at the beginning of co-culture. Representative histograms show DNA signals in F4/80^−^Ter119^+^ cells from the indicated groups. The gating strategy is illustrated in the top panels. **d**, Quantitative analysis of reticulocyte (Ter119^+^DNA^−^) percentages in c. Data represent mean ± SD from n = 3 independent biological replicates in each group. The comparison was evaluated using a One-Way ANOVA. **e**, Same as c, except that the BMDMs were transduced with mCherry-tagged C1qa or Tryobp, and no C1q protein was added. n = 6 in each group except in the control group (n = 5). Data represent mean ± SD. The comparison was evaluated using a One-Way ANOVA. **f**, Same as e, the percentage of EBI-forming macrophages was calculated as the number of mCherry^+^ macrophages surrounded by ≥5 mCherry negative cells divided by the total number of mCherry^+^ macrophages. n = 6 in each group except in the + Macrophage with Tyrobp group (n = 5). Data represent mean ± SD. The comparison was evaluated using a One-Way ANOVA. **g**, Total BM cells from 2-month-old C1qa knockout (KO) or WT littermate control mice were transplanted into lethally irradiated WT recipient mice (CD45.1+). CBCs were performed two months after the transplant. WT, n = 14; C1qa KO, n = 15. The comparisons were evaluated using Student’s t-tests. **h**, Representative histogram showing DNA signals in CD45-CD11b-Ly6C/6G-Ter119+ cells from the bone marrow of indicated mice at 1 year of age. Source data

C1q belongs to the C1 complex of the classic complement activation pathway. It has multiple functions in addition to complement activation in various cell types, particularly in macrophages, in which it is highly and specifically expressed (Extended Data Fig. 6a). C1q has been shown to facilitate macrophage phagocytosis by binding to phosphatidylserine^30^, which is known to be exposed on the surface of pyrenocytes after erythroid enucleation^31^. Indeed, we found that the C1q protein level detected by flow cytometry was particularly high in pyrenocytes compared to erythroblasts and reticulocytes in mouse bone marrow under stress (Extended Data Fig. 6b). We next co-cultured mouse erythroblasts in erythropoietin (Epo) medium with bone marrow-derived macrophages (BMDMs), which led to a significant reduction in pyrenocytes. Given that not all the BMDMs are C1q^+^, we further included recombinant C1q protein into the medium, which resulted in a further decrease in pyrenocytes (Fig. 2o and Extended Data Fig. 6c). Reticulocyte count, on the other hand, was not affected (Extended Data Fig. 6d). We next confirmed these data by overexpressing C1qa or Tyrobp in the BMDMs using vectors that co-express an mCherry reporter. We found reduced pyrenocytes in the erythroblasts co-culture system with C1qa-expressing BMDMs, but not those overexpressing Tyrobp (Extended Data Fig. 6e). BMDMs overexpressing Tyrobp also showed significantly decreased EBI structures surrounding these mCherry^+^ cells (Extended Data Fig. 6f), consistent with our spatial transcriptomic and imaging findings. To reveal the role of C1q in vivo, we transplanted total bone marrow cells from C1qa knockout mice into lethally irradiated recipient mice. We found that the recipient mice exhibited a mild reduction in red blood cell count at a young age (Extended Data Fig. 6g). Importantly, we found a significantly increased amount of pyrenocytes in the bone marrow of C1qa knockout mice, which is minimal in wild-type mice (Fig. 2p and Extended Data Fig. 6h). These findings further support that C1q is involved in the clearance of pyrenocytes. Interestingly, C1qa deficiency in the mouse fetal liver did not overtly alter the spatial correlations of various cell types (Supplementary Figs. 2 and 3), suggesting that C1q is dispensable for the structural assembly of EBIs.

### Human EBIs are macrophage-independent erythroid clusters

**Fig. 3:**
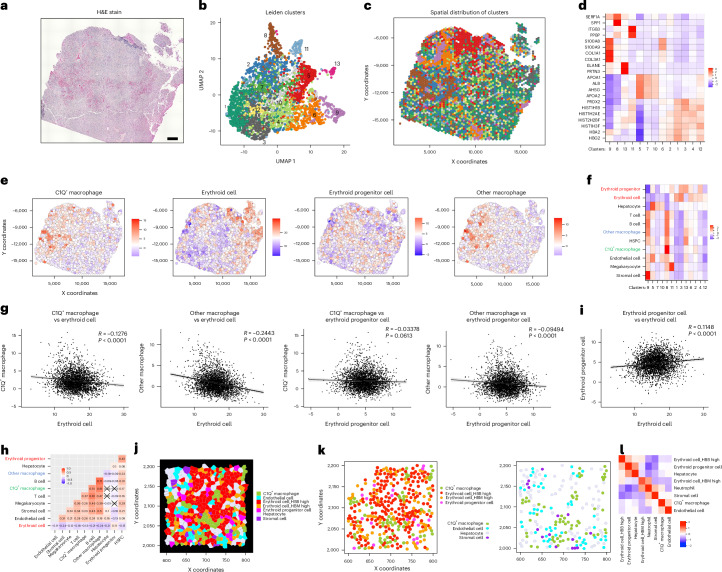
Macrophage-independent EBIs in human fetal liver. **a**, H&E stains of formalin-fixed, paraffin-embedded human fetal liver from a 16 week gestational age embryo analyzed in the spatial transcriptomic analyses. **b**, Leiden clustering of the Visium spatial transcriptomic data shown in **a**. **c**, Spatial distributions of all clusters shown in **b**. **d**, Specific marker genes of all clusters shown in **b**. **e**, In situ distributions of indicated cell types. Scale indicates the enrichment of the indicated cell type within each dot. **f**, Cell type enrichment heatmap of the indicated cell types in different clusters. **g**, Pair-wise correlation analyses of indicated macrophages with erythroid cells or erythroid progenitor cells. Each dot represents one Visium capture spot. Statistical analysis was performed using Pearson correlation (two-sided). Shaded bands represent the 95% confidence interval of the linear regression. Pearson correlation coefficient (*R*) and *P* value are shown. **h**, Correlation *R* values between every two cell types. A positive *R*-value indicates a significant positive correlation, a negative *R*-value indicates a significant negative correlation and an *R*-value marked with ‘X’ indicates a non-significant correlation. **i**, Pair-wise correlation analysis of erythroid progenitor cells and erythroid cells. **j**, Xenium spatial transcriptomic in situ cell types mapping in a selected region of the same sample as in **a**. **k**, In situ mapping of indicated cells in **j**, illustrated as colored circles. **l**, Heatmap showing interactions between two cell types from the Xenium spatial transcriptomic data. Source data

**Extended Data Fig. 7:**
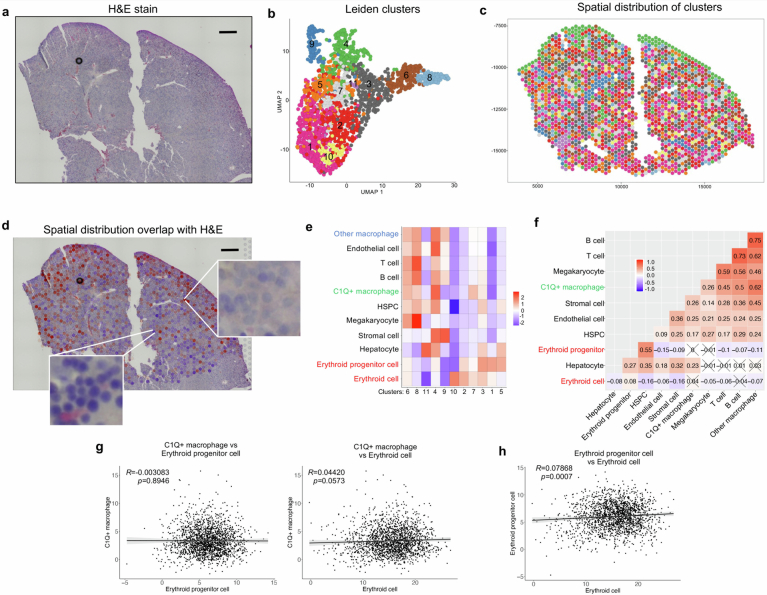
Visium spatial transcriptomic assays of a 12-week gestational age human embryo. **a**, H&E stains of formalin-fixed paraffin-embedded human fetal liver from a 12-week gestational age embryo analyzed in the spatial transcriptomic analyses. Scale bar: 300 μm **b**, Leiden clustering of the Visium spatial transcriptomic data in a. **c**, Spatial distributions of all clusters in b. **d**, Overlap of H&E stain with Visium spatial distribution clusters of C1q+ macrophages. Representative high-power H&E images of the C1q+ macrophage-enriched red clusters and less-enriched blue clusters are illustrated in the insert panels. Scale bar: 300 μm. **e**, Cell type enrichment heatmap of the indicated cell types in different clusters. **f**, Correlation R values between every two cell types. A positive R-value indicates a significant positive correlation, a negative R-value indicates a significant negative correlation, and an R-value marked with “X” indicates a non-significant correlation. **g-h**, Correlation analyses between indicated different cell types. Each dot represents one Visium capture spot. Statistical analysis was performed using Pearson correlation (two-sided). Shaded bands represent the 95% confidence interval of the linear regression. Pearson correlation coefficient (R) and p-value are shown. Source data

To systematically investigate human EBI composition in vivo, we performed spatial transcriptomic assays on fetal liver from a 16 week gestational age embryo under conditions matched to mouse fetal liver analyses (Fig. 3a). Visium profiling identified 13 cell clusters (Fig. 3b), with a more heterogeneous spatial landscape than observed in mice (Fig. 3c). Notably, clusters expressing erythroid-specific genes (*HBA2*, *HBG2*) were spatially segregated from macrophage-specific clusters (*SERF1A*, *SPP1*) (Fig. 3d). Integration with a published scRNA-seq dataset^32^ revealed no spatial association between erythroid cells and C1Q^+^ or other macrophages (Fig. 3e,f and Supplementary Fig. 4a,b), confirmed by quantitative correlation analyses (Fig. 3g,h). Instead, only erythroid and erythroid progenitor cells showed a positive spatial correlation (Fig. 3h,i), whereas hematopoietic stem and progenitor cells (HSPCs) correlated with most cell types (Fig. 3f,h). Xenium spatial analysis similarly demonstrated proximity between erythroid and erythroid progenitor cells (Fig. 3j,k, Supplementary Fig. 4c,d and Supplementary Table 4). C1Q^+^ macrophage-adjacent erythroid cells were observed, but not exclusively. Other cell types were also proximal (Fig. 3j,k), as further supported by unbiased correlation analysis (Fig. 3l). Collectively, these data indicate that erythroid lineage cells cluster preferentially with erythroid progenitors, independent of macrophage proximity. Macrophage-independent EBIs varied in size, with an average diameter comparable to that of mice. Within an 80 µm boundary, each human fetal liver EBI contained an average of 50.45 ± 2.35 (mean ± s.d.) erythroblasts. Visium analysis of a 12 week gestational age fetal liver confirmed the absence of macrophage–erythroid spatial proximity (Extended Data Fig. 7a–h), although erythroid–erythroid progenitor spatial correlation was weaker than at 16 weeks, suggesting a more dispersed erythroid distribution at earlier embryonic stages (Extended Data Fig. 7h).

**Fig. 4:**
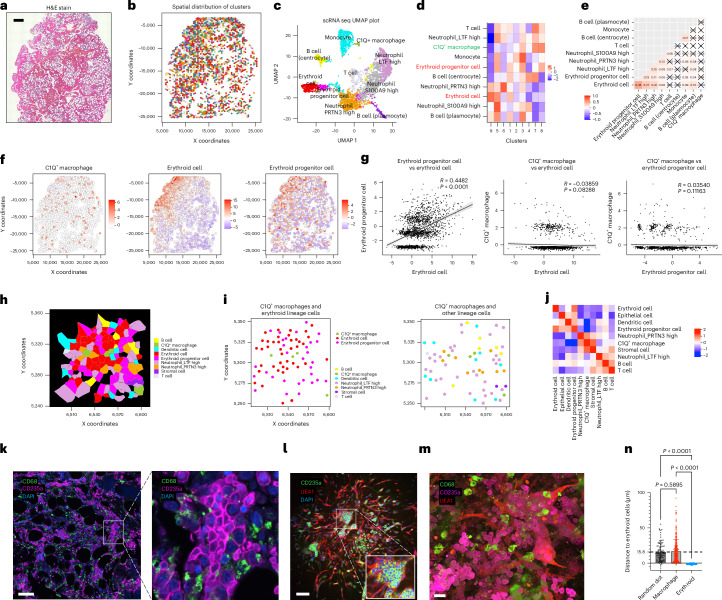
Macrophage-independent EBIs in adult human bone marrow. **a**, H&E stains of a bone marrow clot section from a 50-year-old adult without hematologic diseases. Scale bar, 150 μm. **b**, Spatial distributions of cell clusters derived from Visium transcriptomic assay of bone marrow clot section from **a**. **c**, Re-analysis of scRNA-seq data from GSM3943045. LTF, lactotransferrin. **d**, Cell type enrichment heatmap of the indicated cell types in different clusters. **e**, Correlation *R* values between every two cell types. A positive *R*-value indicates a significant positive correlation, a negative *R*-value indicates a significant negative correlation and an *R*-value marked with ‘X’ indicates a non-significant correlation. **f**, In situ distributions of indicated cell types. Scale indicates the enrichment of the indicated cell type within each dot. **g**, Correlation analyses between erythroid progenitor cells and erythroid cells (left), C1Q^+^ macrophages and erythroid cells (middle) and C1Q^+^ macrophages and erythroid progenitor cells (right). Each dot represents one Visium capture spot. Statistical analysis was performed using Pearson correlation (two-sided). Shaded bands represent the 95% confidence interval of the linear regression. Pearson correlation coefficient (*R*) and *P* value are shown. **h**, Xenium spatial transcriptomics showing in situ mapping of all cell types in a selected region of adult human bone marrow clot section shown in **a**. **i**, In situ mapping of C1Q^+^ macrophage, erythroid cell and erythroid progenitor cell (left), as well as C1Q^+^ macrophage and other cell types except erythroid cell and erythroid progenitor cell (right) in the same region shown in **h**. **j**, Heatmap showing interactions between two cell types from the Xenium spatial transcriptomic data of the whole bone marrow clot section shown in **a**. **k**, Representative confocal immunofluorescent imaging of bone marrow core biopsy from a 52-year-old individual without hematologic diseases. The image on the right is the magnification of a focal spot. Magenta, CD235a^+^ erythroid cells; blue, DAPI; green, CD68 monocytic/macrophage marker. Scale bars, 100 μm. **l**, Representative confocal whole-mount imaging of iPS cell-derived human bone marrow organoids. Whole-mount prepared bone marrow organoids were stained with indicated antibodies, including CD235a for erythroid cells and UEA1 for endothelial cells. Scale bar, 150 μm. **m**, Same as **l**, except that CD235a, UEA1 and CD68 were co-stained. Scale bar, 100 μm. **n**, Quantitative analyses of the distances between the macrophages, random dots or erythroblasts and their closest erythroblast on the whole-mount immunofluorescence imaging using Imaris microscopy image analysis software in **m**. Random dot, *n* = 89; macrophage, *n* = 305; erythroid, *n* = 918. Data represent mean values evaluated using a one-way ANOVA; error bars, s.d. Source data

**Extended Data Fig. 8:**
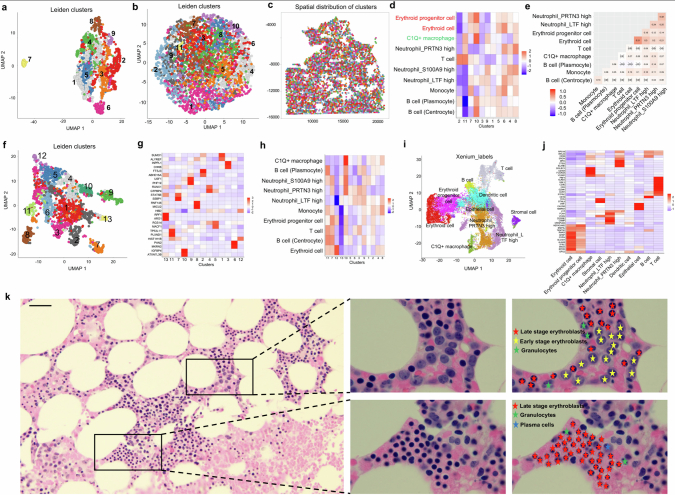
Macrophage-independent EBIs in adult human bone marrow. **a**, Leiden clustering of the Visium spatial transcriptomic data in the human bone marrow sample from a 50-year-old individual without hematologic diseases. **b**, Leiden clustering of the Visium spatial transcriptomic data from the same bone marrow sample in Fig. 4a but in a different tissue section. **c**, Spatial distributions of all clusters in b. **d**, A heatmap showing cell type enrichment in different clusters shown in b. **e**, Correlation R values between every two cell types. A positive R-value indicates a significant positive correlation, a negative R-value indicates a significant negative correlation, and an R-value marked with “X” indicates a non-significant correlation. **f**, Leiden clustering of combined Visium spatial transcriptomic data in human bone marrow samples from 3 additional middle-aged individuals (40 - 60 years old) without hematologic diseases. **g**, Specific marker genes of all clusters in f. **h**, A heatmap showing cell type enrichment in different clusters shown in f. **i**, UMAP plot derived from Xenium subcellular spatial transcriptomic data showing different cell types in normal human bone marrow. **j**, Specific marker genes of all cell types in i. **k**, H&E staining of the bone marrow core biopsy from an individual without hematologic diseases. The pictures in the middle panels highlight the macrophage-independent EBIs. Cells are annotated as indicated on the right panels. Scale bar: 200 μm.

Decalcification-induced RNA degradation poses a major obstacle for spatial transcriptomic studies of human bone marrow clinical samples. To circumvent this issue, we used bone marrow clot sections, routinely prepared in clinical settings for morphological, molecular and immunohistochemical evaluation, which do not require decalcification. To this end, we performed the Visium spatial transcriptomic assay from the bone marrow clot section of a 50-year-old individual without hematologic diseases (Fig. 4a), which revealed nine clusters (Extended Data Fig. 8a). Compared to the human fetal liver, the spatial localization of the hematopoietic clusters in human bone marrow exhibits even greater heterogeneity (Fig. 4b). We then applied two published normal human bone marrow scRNA-seq datasets with matched ages as reference cell signatures for cell type enrichment analyses (Fig. 4c)^33,34^. Compared to the mouse hematopoietic tissues, human adult bone marrow contains a reduced number of macrophages, which are mainly C1Q^+^ (Fig. 4c and Supplementary Table 5).

Combined spatial and scRNA-seq analyses revealed distinct distributions of C1Q^+^ macrophages versus erythroid and erythroid progenitor cells, while erythroid and erythroid progenitor cells co-distributed closely. This was confirmed by correlation analyses showing no macrophage–erythroid association but strong erythroid–erythroid progenitor spatial correlation (Fig. 4d–g). These findings were reproduced in an independent section of the same sample (Extended Data Fig. 8b–e) and validated in bone marrow clot sections from three additional healthy middle-aged individuals (Extended Data Fig. 8f–h). Xenium data corroborated close erythroid lineage association (Fig. 4h and Extended Data Fig. 8i,j), with an average macrophage-independent EBI size of 38.9 ± 3.5 (mean ± s.d.) cells per 80 µm diameter. C1Q^+^ macrophages were instead surrounded by diverse cell lineages, with preferential association with stromal cells and neutrophils (Fig. 4h–j). Collectively, these data demonstrate that human EBIs are structurally distinct from murine EBIs. We note that although macrophage-independent EBIs predominate, mixed-lineage islands containing macrophages and stromal cells also exist in human bone marrow.

**Extended Data Fig. 9:**
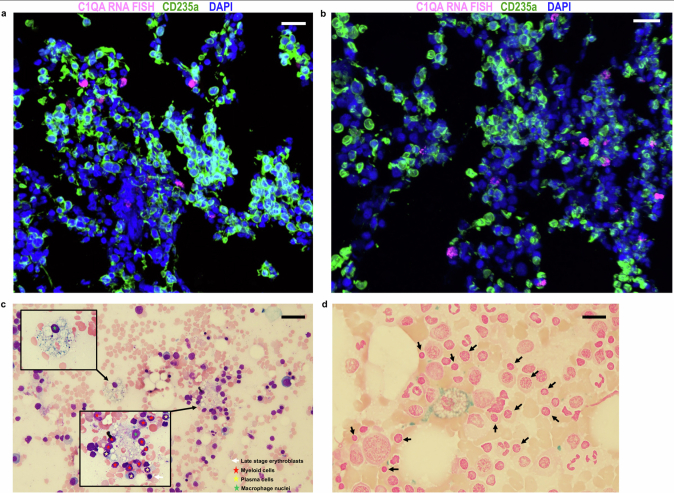
Human erythroid islands do not contain macrophages. **a-b**, Representative images of combined C1QA RNA FISH with immunofluorescence stain of CD235a on an adult human bone marrow without hematologic diseases in an area enriched with macrophage-independent erythroid islands (a) and an area that is less enriched (b, note: many mature red blood cells that lack DAPI stains were present). Scale bar: 50 µm. **c**, A representative Wright-Giemsa stain of bone marrow aspirate smear from the individual in a-b. Cells are annotated as indicated on the bottom right. Scale bar: 60 μm. Inserted figures highlight the macrophages and their surrounding cells. **d**, Prussian blue iron stains of bone marrow smear from the individual in a-b. The green-stained iron-enriched macrophage is highlighted. Black arrows indicate erythroblasts at different stages of differentiation. Scale bar: 40 μm. For a-d, data is representative of 6 independent normal human bone marrow core biopsies analyzed.

To further validate macrophage-independent EBIs in humans, we analyzed bone marrow core biopsies from five healthy middle-aged individuals. A representative hematoxylin and eosin (H&E) stain revealed erythroid-dominated EBIs lacking central macrophages (Extended Data Fig. 8k), and confocal imaging confirmed that EBIs comprised predominantly CD235a^+^ erythroid cells. CD68 staining, a monocyte/macrophage marker targeting lysosomal-associated membrane proteins, showed granular staining distributed throughout the marrow but peripheral to, rather than within, EBIs (Fig. 4k). C1QA RNA fluorescence combined with CD235a immunofluorescence confirmed that most erythroid progenitors form macrophage-independent clusters, with C1QA^+^ macrophages randomly distributed and frequently surrounded by CD235a^−^ cells (Extended Data Fig. 9a,b and Supplementary Movies 1 and 2). Wright–Giemsa and Prussian blue iron stains of bone marrow aspirates further showed macrophages typically associated with diverse hematopoietic lineages or appearing in isolation (Extended Data Fig. 9c,d).

These findings were replicated using an induced pluripotent stem cell (iPS cell)-derived bone marrow organoid model^35–38^. Similar to the primary human bone marrow, CD235a^+^ erythroid cells form macrophage-independent clusters (Fig. 4l). Whole-mount imaging analysis of the bone marrow organoid further revealed scattered CD68^+^ monocytes and macrophages across different regions, often near the vasculature. Notably, most CD68^+^ cells are not surrounded by erythroid cells. Instead, developing erythroid cells form clusters independently throughout the bone marrow organoid (Fig. 4m). Specifically, the mean distances from random spheres to the nearest erythroblasts and from macrophages to their nearest erythroblasts are both approximately 16 µm in the whole-mount imaging (Fig. 4n). By contrast, the distance between each erythroblast and its nearest erythroblast is close to zero, indicating tight clustering among erythroid cells (Fig. 4n). These findings underscore that macrophages are not closely associated with erythroblasts and may not have a pivotal role in generating the EBI structure in human bone marrow.

### EBIs under stress in mice and humans

**Fig. 5:**
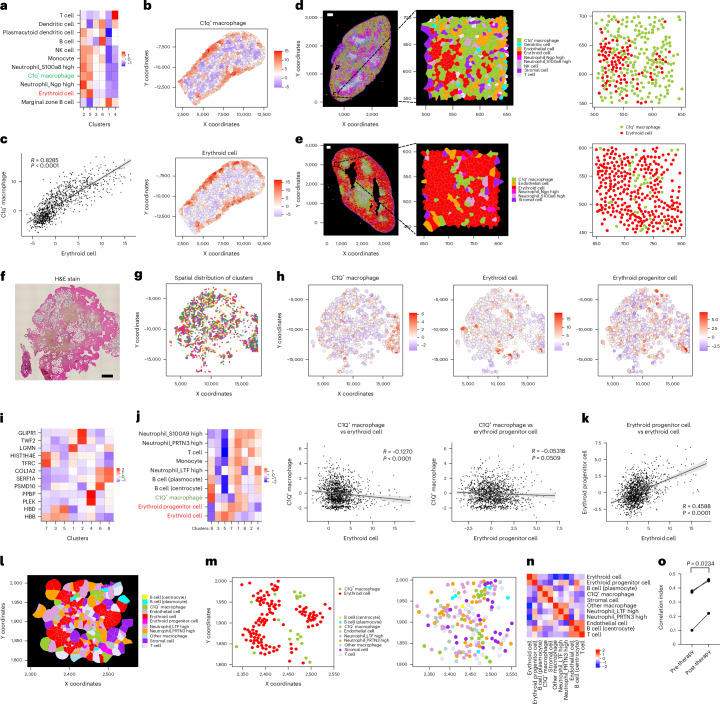
EBIs under stress in mice and humans. **a**, Heatmap showing cell type enrichment in different clusters of a Visium spatial transcriptomic assay performed on the spleen from a 2-month-old wild-type mouse. **b**, In situ distributions of indicated cell clusters. **c**, Correlation between C1q^+^ macrophages and erythroid cells from the Visium spatial transcriptomic analysis. **d**,**e**, Xenium spatial transcriptomics showing in situ mapping of all cell types (left two panels) and selective C1q^+^ macrophages and erythroid cells (right) in the spleen from wild-type mice (**d**) and PHZ-treated mice (**e**). Scale bars, 300 μm. **f**, H&E staining of the bone marrow clot section from a patient with MDS after treatment. Scale bar, 250 μm. **g**, Spatial distributions of cell lineage clusters of the Visium spatial transcriptomic data from the bone marrow clot section in **f**. **h**, In situ distributions of indicated cell clusters. Scale indicates the enrichment of the indicated cell type within each dot. **i**, Specific marker genes of all clusters shown in **g**. **j**, Heatmap showing cell type enrichment results. Specific correlation analyses between C1Q^+^ macrophages and erythroid cells and C1Q^+^ macrophages and erythroid progenitor cells are presented in the right two panels. **k**, Correlation analyses between erythroid cells and erythroid progenitor cells. For **j** and **k**, each dot represents one Visium capture spot. Statistical analysis was performed using Pearson correlation (two-sided). Shaded bands represent the 95% confidence interval of the linear regression. Pearson correlation coefficient (*R*) and *P* value are shown. **l**, Xenium spatial transcriptomics showing in situ mapping of all cell types on the clot section from the same patient with MDS as shown in **f**. **m**, In situ mapping of C1Q^+^ macrophage and erythroid cell (left) and C1Q^+^ macrophage and other cell types (right). **n**, The heatmap assay showing interactions between each pair of cell types from the Xenium data. **o**, Statistical analyses of correlation index *R* values between erythroid cells and erythroid progenitor cells in patients with MDS before and after therapy (*n* = 3 biologically independent patients). The comparison was evaluated using a paired *t*-test. NK cell, natural killer cell. Source data

The spleen is a critical extramedullary hematopoietic organ in mice, particularly under stress. Spatial transcriptomic assays identified C1q^+^ macrophages and erythroid clusters in the red pulp and lymphoid clusters in the white pulp (Supplementary Fig. 5a–d and Fig. 5a,b). Correlation analysis revealed a stronger C1q^+^ macrophage–erythroid spatial association in the spleen than in fetal liver or bone marrow (Fig. 5c), underscoring the importance of splenic EBI macrophages, a population notably larger in the spleen than in other tissues. These findings were confirmed by Xenium analysis (Fig. 5d and Supplementary Fig. 5e–g). Following phenylhydrazine (PHZ)-induced hemolytic anemia, significant splenomegaly and extramedullary erythropoiesis were observed. Xenium studies showed expanded C1q^+^ macrophage-centered EBIs with markedly increased erythroid cells (Fig. 5e and Supplementary Fig. 5h,i) and a higher average erythroid cell count per EBI (mean ± s.d., 52.4 ± 2.6 per 80 µm diameter), probably reflecting reduced erythroid cell size. Although C1q^+^ macrophage-centered EBI numbers expanded, their frequency normalized to total erythroid and macrophage cells remained unchanged (Supplementary Fig. 5j). By contrast, macrophage-independent erythroid interactions increased significantly even after normalization (Supplementary Fig. 5k), suggesting an enhanced functional role for macrophage-independent EBIs during hemolytic stress. As in fetal liver, C1qa deficiency did not alter spatial correlations among cell types in the spleen (Supplementary Fig. 6).

**Extended Data Fig. 10:**
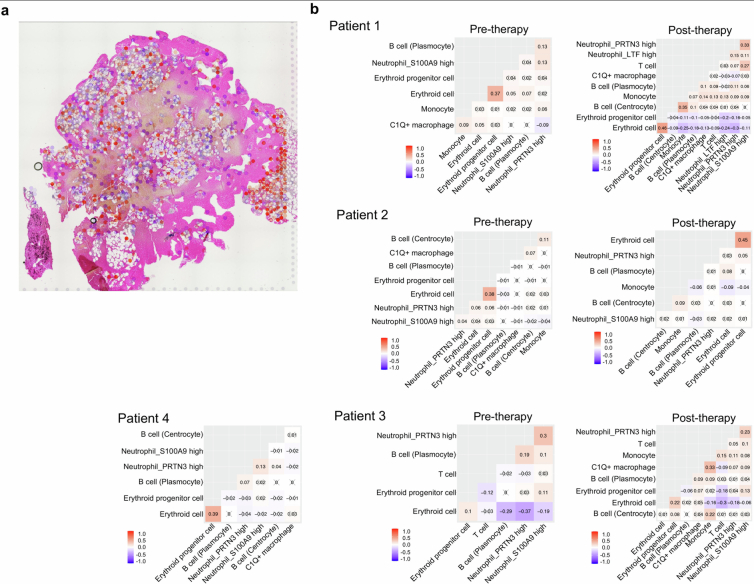
Spatial correlation studies of different cell types in patients with MDS. **a**, Overlap of H&E stain with Visium spatial distribution clusters of C1Q+ macrophages in Fig. 5h. **b**, Correlation R values between every two cell types of the Visium spatial transcriptomic assays from 7 bone marrow clot sections of MDS patients (including the specimen from a, patient 1 post-therapy). 6 of these samples were from 3 patients under pre-therapy and post-therapy conditions. A positive R-value indicates a significant positive correlation, a negative R-value indicates a significant negative correlation, and an R-value marked with “X” indicates a non-significant correlation.

The spleen has a minimal role in stress erythropoiesis in humans, as the bone marrow adequately meets increased erythropoietic demands under stress^39^, such as erythroid regeneration following therapies for hematologic diseases. To this end, we analyzed spatial transcriptomic profiles from the bone marrow clot sections of a patient with anemia due to myelodysplastic syndromes (MDS) after therapies with hypomethylating agents (Fig. 5f). As in healthy individuals, post-therapy MDS bone marrow showed heterogeneous cell distribution (Fig. 5g), no significant spatial association between C1Q^+^ macrophages and erythroid or progenitor cells (Fig. 5h–j and Extended Data Fig. 10a) and strong erythroid–erythroid progenitor spatial proximity (Fig. 5k), which were confirmed by Xenium analysis and unbiased correlation studies (Fig. 5l–n). Visium assays on bone marrow clots from six additional MDS cases, including three pre-therapy and post-therapy pairs, revealed that erythroid–erythroid progenitor spatial correlation was consistently higher post-therapy than pre-therapy (Extended Data Fig. 10b, Supplementary Fig. 7 and Fig. 5o), suggesting that reduced erythroid cell connectivity is a dysplastic feature of MDS that is partially restored with treatment.

### ICAM4 mediates human macrophage-independent EBI formation

**Fig. 6:**
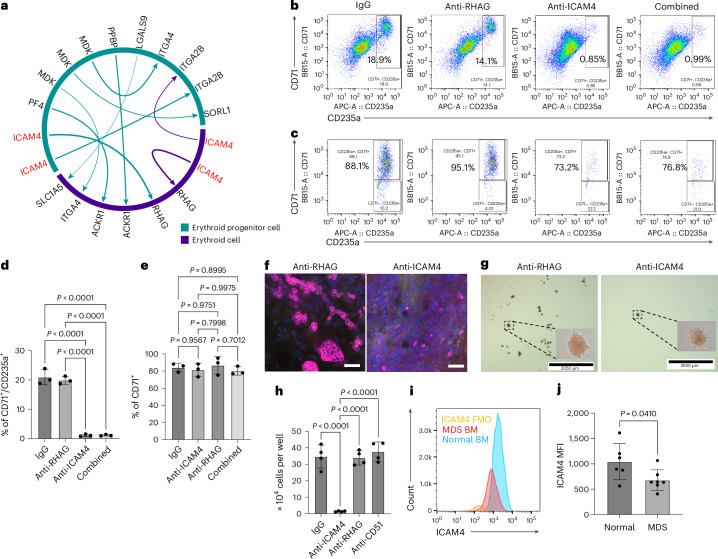
ICAM4 mediates the formation of macrophage-independent EBIs in humans. **a**, Circos plot showing top ten ligand–receptor pairs mediating erythroid cell–erythroid progenitor cell interactions derived from scRNA-seq data GSM3943045 and GSM3396161 from human adult bone marrow. **b**, Flow cytometry analyses of CD71 and CD235a expression levels in human iPS cell-derived bone marrow organoids treated with anti-ICAM4, anti-RHAG or combined antibodies. **c**, Same as **b** except that CD235a^+^ cells were gated for the quantification of CD71^+^ cells. **d**,**e**, Quantitative analyses of **b** (**d**) and **c** (**e**), respectively. Data represent mean values from *n* = 3 independent biological replicates in each group; error bars, s.d. The comparisons were evaluated using one-way ANOVA tests. **f**, Representative pictures of human iPS cell-derived bone marrow organoids stained with CD235a (magenta) and DAPI after anti-ICAM4 or anti-RHAG antibody treatment. Scale bar, 50 μm. The data represent three independent biological replicates. **g**, Representative pictures of erythroid colonies in HSPC colony assays cultured in erythropoietin MethoCult medium, which were treated with the indicated antibodies. **h**, Quantitative analyses of **g**. Data represent mean values from *n* = 4 independent biological replicates in each group; error bars, s.d. The comparison was evaluated using a one-way ANOVA. **i**, Flow cytometry analysis of ICAM4 levels in the bone marrow CD71^+^/CD235a^+^ erythroid precursors in MDS and normal (lymphoma-staging negative) patients. **j**, Quantification of **i**. Normal, *n* = 6; MDS, *n* = 7. Data represent mean values evaluated using a Student’s *t*-test; error bars, s.d. MFI, mean fluorescence intensity. Source data

Studies over the past decade have revealed many receptor–ligand pairs involved in mouse macrophage-centered EBI formation^2,40^. In our spatial data, these cell-surface molecules are predominantly detected in C1q^+^ macrophages (Fig. 1j,k). To identify receptor–ligand pairs mediating macrophage-independent EBI formation in humans, we analyzed a human bone marrow scRNA-seq dataset and found ICAM4–RHAG and ICAM4–ITGA2B as prominent interactions among erythroid and erythroid progenitor cells, highlighted in a Circos plot (Fig. 6a and Supplementary Fig. 8a). The equivalent analysis in mouse fetal liver identified known macrophage–erythroid pairs, including Vcam1–Itga4, consistent with macrophage-centered EBI organization (Supplementary Fig. 8b).

ICAM4 is highly expressed in erythroid cells (Supplementary Fig. 8c). ICAM4 level increases during the early stages of human terminal erythropoiesis (CD235a^+^CD71^+^), followed by a moderate decline in the late stage (CD235a^+^CD71^med^) (Supplementary Fig. 9). These findings suggest that ICAM4 may facilitate the formation and cohesion of erythroid islands during early differentiation, while its downregulation at later stages may promote the release of mature erythroid cells from the niche. To test the function of ICAM4, we applied the iPS cell-derived human bone marrow organoid model and treated the organoids with antibodies against ICAM4. Flow cytometry assays revealed that anti-ICAM4, but not anti-RHAG or control IgG, significantly reduced the number of CD71^+^/CD235a^+^ erythroid cells (Fig. 6b,d) without affecting their differentiation (Fig. 6c,e). A confocal imaging assay further showed that blocking ICAM4 significantly reduced the number and size of macrophage-independent EBIs (>5 cells per cluster) (Fig. 6f and Supplementary Fig. 10a).

ICAM4 was reported to interact with macrophage α_v_-integrin in mouse macrophage-centered EBIs^41^. To exclude the remote possibility that the disrupted EBIs were a result of macrophage–erythroid interactions, we performed an in vitro colony-forming unit assay using a medium containing only Epo, which differentiates the human CD34^+^ HSPCs exclusively into erythroid lineages. Consistent with the organoid data, we found a significant reduction in erythroid colonies when HSPCs cultured in Epo medium were treated with anti-ICAM4. By contrast, cells treated with IgG, anti-RHAG or anti-α_v_-integrin showed no changes in erythroid island formation (Fig. 6g,h). These findings support that ICAM4 is critical for the formation of macrophage-independent EBIs in humans. Interestingly, the level of ICAM4 was significantly reduced in the erythroblasts from the bone marrow of patients with MDS compared to those from healthy individuals (Fig. 6i,j and Supplementary Fig. 10b), which further suggests that the compromised integrity of macrophage-independent EBIs by reduced ICAM4 expression could be a dysplastic feature of MDS.

## Discussion

Our study reveals fundamental species differences in EBI anatomy and molecular mechanisms. In mice, canonical macrophage-centered EBIs persist throughout development, with C1q serving as a hallmark of the central macrophage. C1q, a component of the classical complement pathway, facilitates macrophage phagocytosis by binding phosphatidylserine exposed on pyrenocyte surfaces following erythroid enucleation. Notably, erythrocytes express C1q receptors (CR1/CD35) that block complement opsonization^42^. Whether pyrenocytes exhibit reduced receptor expression warrants future investigation. In addition to phagocytosis, C1q regulates MerTK expression on macrophages to promote nuclear clearance^43^ and limits inflammasome activity during apoptotic cell uptake^44^, both of which are critical for maintaining bone marrow homeostasis. Collectively, these functions align with known EBI macrophage roles in mice, supporting C1q as a key marker of macrophage heterogeneity.

In contrast to mice, human hematopoietic tissues are predominantly composed of macrophage-independent erythroid clusters. Although macrophage-centered EBIs are critical for mouse erythropoiesis, human erythroid maturation exhibits far greater spatial autonomy. Conventional macrophage-centered EBIs do occur in humans but are rare and stochastic. We found that ICAM4 is critical for the integrity of human EBIs. ICAM4 is an erythroid-specific glycoprotein originally identified as the LW blood group antigen and subsequently classified among the intercellular adhesion molecules^45^. ICAM4 interacts with different integrins, including CD11a/CD18 (LFA-1)^45,46^, CD11b/CD18 (Mac-1)^46^, CD11c/CD18 (ref. ^47^) and α_v_-integrin^48^. However, these surface proteins are predominantly expressed in leukocytes. Before this study, the mechanisms by which ICAM4 mediates spatial organization among human erythroid progenitors were unknown. We demonstrate that ICAM4 is essential for human EBI integrity and erythroid proliferation. Parallel ligand–receptor analysis in mouse fetal liver identified canonical macrophage–erythroid interaction pairs, consistent with macrophage-centered murine organization. Together, these findings suggest that species-specific differences in intercellular adhesion programs underlie the divergent erythroid niche architectures in mice and humans, potentially reflecting upstream differences in transcriptional regulation by master erythroid regulators such as GATA1, KLF1 or TAL1, which require further investigation.

Notably, macrophage-independent erythroid clusters are also prevalent in mouse hematopoietic tissues. Spatial transcriptomic data reveal that erythroid–erythroid spatial correlation indices exceed those between erythroid cells and C1q^+^ macrophages, suggesting that erythroid clustering is a conserved feature of the erythropoietic niche. Evolutionarily, macrophage-centered EBIs appear to have diminished in humans, becoming rare and stochastic. This architectural divergence may partly reflect differences in the extracellular matrix: in mice, fibronectin and laminin support erythroid adhesion and terminal differentiation^2^, potentially stabilizing macrophage-centered EBIs, whereas human bone marrow, characterized by greater adiposity and reduced cellularity^39,49,50^, may impose different spatial constraints that reduce reliance on macrophage anchoring. Together, species-specific variation in extracellular matrix composition and adhesion molecule programs probably shapes the divergent erythroid clustering strategies observed across species, warranting further investigation.

Although human macrophages are not anatomically integrated into EBIs, they may perform analogous functions in a paracrine manner, including pyrenocyte phagocytosis and nutrient supply to erythroid cells. C1Q^+^ macrophages in human bone marrow may also compensate for EBI defects in LW-null individuals. The predominance of macrophage-independent erythroid clustering in both species raises important questions about pyrenocyte clearance, such as whether other phagocytes are involved, and whether alternative clearance mechanisms exist beyond macrophage phagocytosis. This study provides a framework for addressing these questions.

## Methods

### Mouse sample collection

All animal studies followed the Guidelines for the Care and Use of Laboratory Animals and were approved by the Institutional Animal Care and Use Committees at Northwestern University (protocol number IS00033835). Wild-type (C57BL/6) mice used in Visium and Xenium spatial transcriptomic assays were purchased from the Jackson Laboratory. C1qa knockout mice were purchased from the Jackson Laboratory (cat. no. 031675). EpoR-tdTomato-Cre mice were kindly provided by X. An from the New York Blood Center. The following timing protocol was used for the collection of embryonic stage and postnatal stage samples: one male and one female mouse per cage were mated in the afternoon of day 1 and separated in the morning of day 2. Subsequently, all female mice were individually housed in new cages and marked as pregnant at 0.5 days after mating.

For fetal liver sample collection, post-mating pregnant mice were killed, and the fetal livers of a specific embryonic stage were collected. For postnatal sample collection, mice on postnatal day 8 were used for bone marrow collection. For spleen collection, normal spleens were collected from wild-type mice at 2 months old. For spleens from PHZ-treated mice, 40 mg kg^−1^ PHZ or vehicle (1× PBS) was injected intraperitoneally twice daily for 2 days. Then, PHZ-treated and control mice were killed, and spleens were collected. The selected tissues for spatial transcriptomics in this study are representative of at least ten independent biological replicates per condition and reflect normal physiological states or defined experimental conditions.

All freshly collected samples were directly fixed in a 10% neutral buffered formalin solution (Sigma-Aldrich) and kept at room temperature (20 °C to 22 °C) for 3 days. Mouse sample embedding and sectioning were performed at the Mouse Histology and Phenotyping Laboratory facility, Northwestern University.

### Human sample collection

Human bone marrow aspirate samples, clot sections and core biopsy blocks were obtained from leftover diagnostic specimens at Northwestern Memorial Hospital. No written consents from patients were required for this study, given that the samples were part of the routine diagnostic materials. The study protocol was approved by the Institutional Review Board (IRB) of Northwestern University (IRB ID STU00217116). Human sample embedding and sectioning were performed at the Pathology Core Facility, Northwestern University.

### Visium spatial transcriptomics

Visium whole transcriptome spatial RNA sequencing platforms (Visium V1) for mice and humans were purchased from 10x Genomics. Formalin-fixed, paraffin-embedded (FFPE) tissue blocks were used for the spatial analyses. To confirm the RNA quality of each FFPE block, one to two curls (10 μm thickness each) of the block were tested using a Qiagen RNeasy FFPE kit (Qiagen, 73504) according to the manufacturer’s protocol. Extracted RNAs were examined using an Agilent Bioanalyzer RNA pico chip to confirm DV200 > 30%. Simultaneously, the tissue morphology was examined using H&E stain to identify the region of interest.

For each FFPE sample, 5 µm sections were placed on Visium slides (four capture areas of 6.5 mm × 6.5 mm each; smaller tissues were co-placed in a single area). Slides were incubated at 42 °C for 3 h, followed by overnight room temperature incubation, then stored in a desiccated chamber until deparaffinization. Deparaffinization, decrosslinking, probe hybridization and ligation were performed using the 10x Genomics FFPE Visium spatial gene expression kit (v2 probes; PN-1000188; protocols CG000409 and CG000407). Probes were released, captured and extended on the Visium slide, and sequencing libraries were prepared per the manufacturer’s protocol. Libraries were sequenced on a NovaSeq 6000 S2 flow cell (100 cycles; 28 nt read 1, 90 nt read 2) at the NUSeq Core, Northwestern University Feinberg School of Medicine.

### Xenium spatial transcriptomics

Xenium transcriptome spatial RNA sequencing platforms for mice and humans were purchased from 10x Genomics. As with the Visium procedure, 5 μm-thick sections were placed on the Xenium slide capture area, followed by 3 h of 42 °C incubation and overnight drying. Slides were stored at room temperature in a desiccation chamber until proceeding to deparaffinization.

Slide processing and Xenium analyzer loading were carried out using Xenium slide and sample prep reagent kit (PN1000460), Xenium decoding consumables (PN-1000487) and Xenium decoding reagent (PN-1000461). Slides were first deparaffinized and de-crosslinked according to the manufacturer’s protocol (CG000580). Then, custom probes with pre-designed panel genes were hybridized to the sample slide overnight, followed by probe ligation and amplification according to the manufacturer’s protocol (CG000582). Autofluorescence quenching and nuclei staining were performed before imaging and decoding signals on the Xenium analyzer. The Xenium slide processing and Xenium analyzer loading were performed at Northwestern University NUSeq core facility.

### scRNA-seq data analyses

All scRNA-seq datasets were obtained from the Gene Expression Omnibus (GEO) database or the European Molecular Biology Laboratory-European Bioinformatics Institute (EMBL-EBI) database. These databases include GSE176063 and GSE172127 (E14.5 mouse fetal liver), GSE108097 (mouse bone marrow and spleen), GSE134355 and GSE120446 (human bone marrow) and E-MTAB-7407 (human fetal liver). The datasets were analyzed using Giotto Suite (v.4.0.5) or Seurat (v.5.0.3) in R (v.4.3.3). For the Giotto Suite and Seurat analyses, the workflows were reported on the following websites: https://giottosuite.com/articles/singlecell_prostate_standard.html and https://satijalab.org/seurat/articles/pbmc3k_tutorial, respectively. In brief, the Giotto object was created after reading barcodes, features (genes) and matrix files. Then, the Giotto object was filtered, using expression threshold = 1, features detected in minimal cells = 50 and minimal detected features per cell = 500, followed by normalization with the scale factor = 6,000. The cells were removed when the percentage of the standard mitochondria gene was more than 15%. Feature and cell statistics were calculated. Dimension reduction was performed by calculating highly variable features and running a principal component analysis. The nearest neighbor network was created, and UMAP was run with the parameter dimensions to use = 1:10. The cells were clustered using a nearest neighbor network and the Leiden community detection algorithm with a resolution of 0.2 to 0.5. For the combination analysis of multiple samples, a harmony integration algorithm was used. Cell marker genes were found and extracted with the scran package. Cell type annotation was done by combining marker genes, web databases including the mouse cell atlas https://bis.zju.edu.cn/MCA, the human cell landscape https://db.cngb.org/HCL and the original published reports. After cell type annotation, cell metadata were extracted for downstream pathway and differential gene expression analysis. Ligand and receptor information in the cell metadata was used to analyze the potential cell–cell interactions.

### Visium spatial gene expression data analyses

For the Visium data, the original fastq datasets were analyzed using Space Ranger (10x Genomics; https://www.10xgenomics.com/support/software/space-ranger/latest/tutorials/count-ffpe-tutorial). The raw feature matrix and matched spatial image files were exported for further analysis with Giotto Suite (v.4.0.5) in R (v.4.3.3). The workflow for Visium transcriptomic data analysis has previously been reported^51^. In brief, the Giotto Visium object was created by reading barcodes, features (genes), matrix and image files, aligning the metadata and images, and transforming tissue barcodes into cell IDs. Then, the Giotto Visium object was filtered, using expression threshold = 1, features detected in minimal cells = 50 and minimal detected features per cell = 1,000, followed by normalization with the scale factor = 6,000. Similarly, feature and cell (spot) statistics were calculated. Dimension reduction was performed by calculating highly variable features and running a principal component analysis. Then, *t*-distributed stochastic neighbor embedding and UMAP were run with the parameter dimensions to use = 1:10. A nearest neighbor network was created, and cells were clustered based on the nearest neighbor network and the Leiden community detection algorithm with a resolution of 0.2 to 0.5. Different clusters were mapped in situ to show their spatial distributions. Marker genes in each cluster were found and extracted with the scran package. Given that each spot contains the mixed transcriptome of several cells and each cluster is equivalent to a bulk RNA-seq dataset, cell type enrichment was performed using the PAGE method, in which 20 marker genes per cell type were derived from the cell metadata of the matched scRNA-seq dataset, and the relative expression scores (also called *z*-scores) of each cell type were calculated. *Z*-scores of all cell types were extracted, and correlations between every two kinds of cells were calculated. The spatial distributions of each cell type were mapped in situ.

### Workflow of Xenium spatial gene expression

For Xenium data, the original fastq datasets were analyzed by Space Ranger (10x Genomics; https://www.10xgenomics.com/support/software/space-ranger/latest/tutorials/count-ffpe-tutorial). Cell feature matrix, cell boundary, nucleus boundary, transcript files and spatial morphology image files were exported for further analysis with Giotto Suite (v.4.0.5) in R (v.4.3.3). The workflow of the Xenium transcriptomic data analysis was previously reported^51^. In brief, the Giotto Xenium object was created after loading the probe, matrix and image files and then integrating the metadata, cell boundary and nucleus boundary images. Then, the Giotto Xenium object was filtered using expression threshold = 1, features detected in minimal cells = 3 and minimal detected features per cell = 5, followed by normalization with the scale factor = 5,000. Feature and cell statistics were calculated. Dimension reduction was performed by calculating highly variable features and running a principal component analysis. Then, *t*-distributed stochastic neighbor embedding and UMAP were run with the parameter dimensions to use = 1:10. A nearest neighbor network was created, and cells were clustered based on the nearest neighbor network and the Leiden community detection algorithm with a resolution of 0.2 to 0.5. Different clusters were mapped in situ to show their spatial distributions. Marker genes in each cluster were found and extracted with the scran package. Finally, cell type annotation was performed by combining marker genes, web databases including the mouse cell atlas https://bis.zju.edu.cn/MCA, human cell landscape https://db.cngb.org/HCL and cell metadata of the matched scRNA-seq dataset. The spatial distributions of each cell type were mapped in situ. The spatial Delaunay network was calculated with maximum distance = 400. Cell proximity enrichment with 1,000 simulations was performed to show potential cell–cell interactions.

### Combined RNA fluorescence in situ hybridization and immunofluorescence staining

Combined RNA fluorescence in situ hybridization and immunofluorescence was performed as per the manufacturer’s instructions (Thermo Fisher Scientific). FFPE blocks were sectioned under RNase-free conditions at the Mouse Histology and Phenotyping Laboratory, Northwestern University, then deparaffinized with HISTO-CLEAR II (National Diagnostics) and heat-pretreated. Following hydrophobic barrier application, samples underwent sequential protease digestion, fixation, probe hybridization, amplification and labeling using mouse C1qa (VB6-14028-VT) and Tyrobp (VB1-19392-VT) ViewRNA Tissue Probe Sets (Thermo Fisher Scientific). Slides were then blocked with Odyssey Blocking Buffer (LI-COR, 30 min, room temperature), incubated with primary antibody (anti-TER-119, 14-5921-82, Thermo Fisher Scientific) overnight at 4 °C, washed four times with 1× PBS/0.1% Tween-20 and incubated with Alexa Fluor 488-conjugated goat anti-rat IgG secondary antibody (1 h, room temperature). After DAPI counterstaining and mounting, images were acquired on a ZEISS microscope.

### Bone marrow transplantation

C1qa-heterogeneous mice were purchased from the Jackson Laboratory and crossed to obtain C1qa knockout mice. Bone marrow transplantation was performed as previously reported^52–54^. In brief, total bone marrow cells from C1qa knockout and age-matched wild-type mice were collected, RBC-lysed and transplanted into lethally irradiated recipients. Then, 2 months post transplantation, peripheral blood was collected for complete blood count analysis, and bone marrow was collected for further study.

### Co-culture of BMDMs and erythroblasts

Macrophages derived from mouse bone marrow were isolated and induced as described in a previous study^55^. In brief, wild-type mice were killed, and total bone marrow cells were isolated from their femurs. Then, the cells were cultured in DMEM/F-12 supplemented with 10% FBS (GeminiBio), 1% penicillin–streptomycin and 100 ng ml^−1^ macrophage colony-stimulating factor (M-CSF; StemCell Technologies) for 7 days. At the same time, lineage-negative cells from the bone marrow of wild-type mice were isolated and purified as described in previous studies^13,56,57^. Then, the lineage-negative cells were co-cultured with BMDMs in a six-well plate. For each well, 1.5 × 10^6^ macrophages and 4.5 × 10^6^ lineage-negative cells were co-cultured in 2 ml fresh Epo-containing medium for 48 h. Pure human C1qa protein was purchased from Abcam (cat. no. ab282858), and 25 µg ml^−1^ C1qa protein and vehicle solution were added at the beginning of co-culture. After co-culture, the cells were collected and analyzed by flow cytometry.

### Cryosection of mouse femur without decalcification

The femur was fixed in 4% paraformaldehyde at room temperature for 72 h, then embedded in 8% gelatin (8 g of gelatin in PBS) and stored at −80 °C for 24 h before cryosectioning following a published report^25^. Cryosectioning was performed at −25 °C, with the femur sectioned into 100 µm-thick slices and collected onto coated microscope slides. These slides were equilibrated to room temperature. During equilibration, differences in the coefficients of expansion between the bone and bone marrow allowed easy separation of the bone from the marrow while preserving the structural integrity of the marrow. The bone marrow was then embedded in paraffin and sectioned onto spatial transcriptomic-dedicated slides for transcriptome analysis.

### Culture of human iPS cells

Human iPS cell line SCTi003-A (cat. no. 200-0510) derived from peripheral blood mononuclear cells from a 48-year-old healthy female donor was purchased from StemCell Technologies. SCTi003-A is karyotypically stable, shows trilineage differentiation potential and expresses markers of the undifferentiated state following reprogramming using a non-integrating reprogramming technology. The iPS cells were maintained in mTeSR Plus medium (StemCell Technologies, 85857) in six-well plates coated with 10% (v/v) hESC-Qualified Matrix (Corning, CLS354277) diluted in DMEM/F-12 medium (Gibco, 10565018). The iPS cells were passaged using ReLeSR (StemCell Technologies, 100-0483) and routinely tested for mycoplasma contamination and karyotype for quality control.

### Bone marrow organoid differentiation

The bone marrow differentiation protocol was derived from the method described previously^35–37^. Specifically, iPS cells were dissociated using ReLeSR when colonies reached approximately 100 μm in diameter during the differentiation phase. The iPS cell aggregates were incubated overnight in mTeSR Plus medium enhanced with RevitaCell in six-well Costar Ultra-Low Attachment plates (Corning, CLS356230; cat. no. 3471). Following this incubation, cells were gathered by gravitation in a 15 ml Falcon tube (Fisher Scientific, 11507411) and resuspended in Phase I medium. This medium consisted of APEL2 (StemCell Technologies, 05275) enriched with bone morphogenic protein 4 (BMP4; Thermo Fisher Scientific, PHC9531), fibroblast growth factor 2 (FGF2; StemCell Technologies, 78134.1) and vascular endothelial growth factor-A (VEGF-165; StemCell Technologies, 78159.1) at 50 ng ml^−1^. The cells were then plated in six-well ultra-low attachment plates and incubated under hypoxic conditions (1% O_2_, 5% CO_2_) for 3 days (days 0–3). After this period, cell aggregates were collected by gravitation and suspended in Phase II medium for an additional 48 h (days 3–5). The Phase II medium included APEL2 supplemented with BMP4, FGF2 and VEGFA at 50 ng ml^−1^, along with human Stem Cell Factor (hSCF; StemCell Technologies, 78062) and Fms-like tyrosine kinase-3 Ligand (Flt3; StemCell Technologies, 78009) at 25 ng ml^−1^. On day 5, cells were again collected by gravity for hydrogel embedding. The hydrogels, composed of 60% collagen (types I and IV mixed at a 1:1 ratio, purchased from Advanced Biomatrix, 5022 and 5007) and 40% Matrigel (Corning, CCLS356230), were prepared on ice according to the manufacturer’s instructions. Next, a 0.5 ml cell-free base layer was laid and allowed to polymerize for 2 h, followed by another 0.5 ml layer containing the cell aggregates, also left to polymerize for 2 h at 37 °C and 5% CO_2_. The fully polymerized gels with cell aggregates were then supplemented with Phase III media. This media included VEGFA at 50 ng ml^−1^, VEGFC, FGF2, BMP4, hSCF, Flt3, Epo (StemCell Technologies, 78007), thrombopoietin (StemCell Technologies, 78210) and granulocytic colony-stimulating factor (StemCell Technologies, 78012) at 25 ng ml^−1^, along with IL-3 (StemCell Technologies, 78194) and IL-6 (StemCell Technologies, 78050) at 10 ng ml^−1^. The media was refreshed every 72 h. On day 12, the organoids were released from the 3D culture matrix and transferred to 96-well ultra-low attachment plates (Thermo Fisher, 174929) in Phase IV medium (APEL2 with 2% FBS, supplemented with VEGFA, VEGFC, FGF2, hSCF and Flt3 at 25 ng ml^−1^, and thrombopoietin, Epo, IL-3 and IL-6 at 10 ng ml^−1^). Phase IV medium was replaced every 72 h at a 50:50 ratio. The organoids are fully matured from day 21 and ready for experiments.

### Bone marrow organoid immunostaining and imaging

Organoids were fixed in 4% paraformaldehyde (30 min, room temperature), permeabilized with 0.25% Triton X-100/PBS (1 h) and blocked with 5% goat serum/0.1% Tween-20/PBS (1 h, orbital shaker). Primary antibodies—anti-CD68 (Abcam, ab783; 1:200), anti-CD235a (Invitrogen, PA5-141179; 1:300) and biotin-UEA1 (Vector Lab, B0-1065-2; 1:300)—were applied overnight at 4 °C on an orbital shaker, followed by three washes with 0.1% Tween-20/PBS. Fluorophore-conjugated secondary antibodies and streptavidin were incubated overnight at 4 °C, washed three times, then once with PBS. Organoids were cleared in 60% glycerol/2.5 M fructose (1 h) and transferred to glass-bottom dishes for imaging.

Whole-mount confocal imaging was performed on a Nikon AXR system using a ×20 water-immersion objective (CFI Apo LWD Lambda S 20XC WI), capturing 100 µm in depth at 0.8 µm Z-steps. Z-stacks were 3D-reconstructed in Imaris 10.0 (Oxford Instruments). To quantify erythroblast–macrophage spatial associations, 90 randomly placed 15 µm-diameter spheres were generated per organoid, and nearest neighbor distances between erythroblasts, macrophages and random spheres were calculated across three independent organoids.

### Flow cytometry analysis of human bone marrow organoids

Organoids were collected by sedimentation, washed twice with PBS and dissociated in 20 mg ml^−1^ collagenase type II (Sigma-Aldrich, C6885) in HEPES buffer (Sigma-Aldrich, H0887) by sequential 5-min incubation at 37 °C, trituration and 15 min incubation. Dissociated cells were resuspended in 5 ml PBS, filtered through a 40 µm strainer, centrifuged (300*g*, 5 min) and resuspended in 100 µl PBS for immunostaining with APC-conjugated anti-CD235a (BioLegend, HI264) and FITC-conjugated anti-CD71 (BioLegend, CY1G4) for 15 min at room temperature. After washing, cells from ten pooled organoids per condition were analyzed by flow cytometry (BD FACSymphony A3) with single-color and fluorescence-minus-one controls.

### Flow cytometry analysis of human bone marrow aspirate samples

Fresh human bone marrow aspirate samples were obtained from leftover diagnostic specimens at Northwestern Memorial Hospital under IRB approval (IRB ID STU00217116). Samples were diluted 1:20 in MACS buffer (PBS containing 2 mM EDTA and 0.5% BSA). A total of 100 µl of the diluted bone marrow was stained with the following antibodies: FITC-conjugated anti-CD71 (BioLegend, clone CY1G4), APC-conjugated anti-CD235a (BioLegend, clone HI264) and BV421-conjugated anti-ICAM4 (BD, clone 729632). Fluorescence-minus-one controls were prepared using BV421-conjugated mouse IgG2b, κ isotype control antibody (BD, clone 27-35), in combination with FITC-conjugated anti-CD71 and APC-conjugated anti-CD235a antibodies. For each sample, at least 5,000 CD71^+^CD235a^+^ events were recorded using a BD FACSymphony A3 flow cytometer. MFI of ICAM4 expression was quantified using FlowJo (v.10.0).

### Multiplexed immunofluorescence staining

FFPE sections were dewaxed and rehydrated at the Mouse Histology and Phenotyping Laboratory, Northwestern University. Antigen retrieval was performed in boiling 1× citric buffer (pH 6.0, Sigma-Aldrich) for 10 min. After cooling and three PBS washes, slides were blocked with Odyssey Blocking Buffer (LI-COR, 30 min, room temperature), incubated with primary antibodies overnight at 4 °C, washed four times with PBS, then incubated with secondary antibodies for 1 h at room temperature. Nuclei were counterstained with Hoechst 33342 (2 µg ml^−1^; Invitrogen, 15 min), washed, coverslipped and imaged on a ZEISS microscope. Primary antibodies included anti-TER-119 (14-5921-82, Thermo Fisher Scientific) and anti-C1QC/C1QG (BS-11337R, Thermo Fisher Scientific). Secondary antibodies were mouse anti-rat IgG2b (50-4815-82) and goat anti-rabbit IgG Alexa Fluor 488 (A11008), both from Thermo Fisher Scientific.

### MethoCult Assay with antibody treatment

Human CD34^+^ HSPCs (StemCell Technologies, 70008.1) were seeded at 1 × 10^3^ per well in six-well plates using EPO-only MethoCult medium (StemCell Technologies, 04330) supplemented with IgG isotype control (Invitrogen, 02-6102), anti-ICAM4 (PA5-112917), anti-RHAG (PA5-51097) or anti-CD51 (MA5-32195) at 12.5 µg ml^−1^. After 14 days at 37 °C and 5% CO_2_, plates were imaged using inverted wide-field microscopy (Evos M5000). Cells were collected by dissolving the MethoCult gel in MACs buffer (0.5% BSA, 2 mM EDTA in PBS) and centrifugation (500*g*, 15 min), counted using an automated cell counter (TC20, Bio-Rad) and immunostained for flow cytometry analysis.

### Statistics and reproducibility

For the comparison of two groups, Student’s *t*-test or Welch’s *t*-test (two-tailed) was used. For comparing more than two groups, a one-way ANOVA was used. For the correlation analysis of two groups of cells, Pearson correlation coefficients were computed, and 95% confidence intervals and two-tailed *P* values were determined. Individual data points are shown in all figures. Data distribution was assumed to be normal, but this was not formally tested. All statistical analyses were performed using R or GraphPad Prism 10.6. No statistical method was used to predetermine sample size. No data were excluded from the analyses. Sample assignment to experimental groups was based on genotype, age or disease status rather than randomization, as the study design involved predefined biological comparisons between distinct groups; no additional randomization was applied to data collection or experimental conditions.

### Reporting summary

Further information on research design is available in the Nature Portfolio Reporting Summary linked to this article.

## Online content

Any methods, additional references, Nature Portfolio reporting summaries, source data, extended data, supplementary information, acknowledgements, peer review information; details of author contributions and competing interests; and statements of data and code availability are available at 10.1038/s41588-026-02671-2.

## Supplementary information

Supplementary Information Supplementary Figs. 1–10.

Reporting Summary

Peer Review File

Supplementary Tables 1–5 Supplementary Tables 1–5.

Supplementary Movie 1 Supplementary Movie 1.

Supplementary Movie 2 Supplementary Movie 2.

Supplementary Figure Source Data Supplementary figure source data.

## Source data

Source Data Figs. 1–6 and Extended Data Figs. 1, 2 and 4–7 Source data for all main figures and extended data figures.

## Data Availability

The scRNA-seq datasets used in this study, including GSE176063 and GSE172127 (E14.5 mouse fetal liver), GSE108097 (mouse bone marrow and spleen), and GSE134355 and GSE120446 (human bone marrow), were obtained from the NCBI GEO database. E-MTAB-7407 (human fetal liver) was obtained from the European Bioinformatics Institute database. All Visium spatial transcriptomic datasets and Xenium spatial transcriptomic datasets were deposited in the NCBI GEO database (GSE271077 and GSE271150 are mouse and human Visium spatial transcriptomic datasets; GSE271693 and GSE271824 are mouse and human Xenium subcellular spatial transcriptomic datasets). Additionally, Extended Data Fig. 6a and Supplementary Fig. 8c were derived from biogps.org and can be found at http://biogps.org/?full#goto=genereport&id=12259 and http://biogps.org/?full#goto=genereport&id=3386, respectively. Source data are provided with this paper.

## Code availability

All R codes, including detailed parameter settings for single RNA-seq datasets, Visium spatial transcriptomic datasets and Xenium spatial transcriptomic datasets, can be found on Zenodo (10.5281/zenodo.20414337)^58^.
