# Supplementary material for: Spatial transcriptomic analyses highlight distinct erythroid niches in mice and humans
Source: Nat Genet. 2026 Jul 2;58(7):1620–31. doi: 10.1038/s41588-026-02671-2 (PMC13364664; doi:10.1038/s41588-026-02671-2)
Supplement: Supplementary file 1 — Supplementary Figs. 1–10. [file 41588_2026_2671_MOESM1_ESM.pdf]

nature genetics

Supplementary information https://doi.org/10.1038/s41588-026-02671-2

Spatial transcriptomic analyses highlight distinct erythroid niches in mice and humans

In the format provided by the authors and unedited

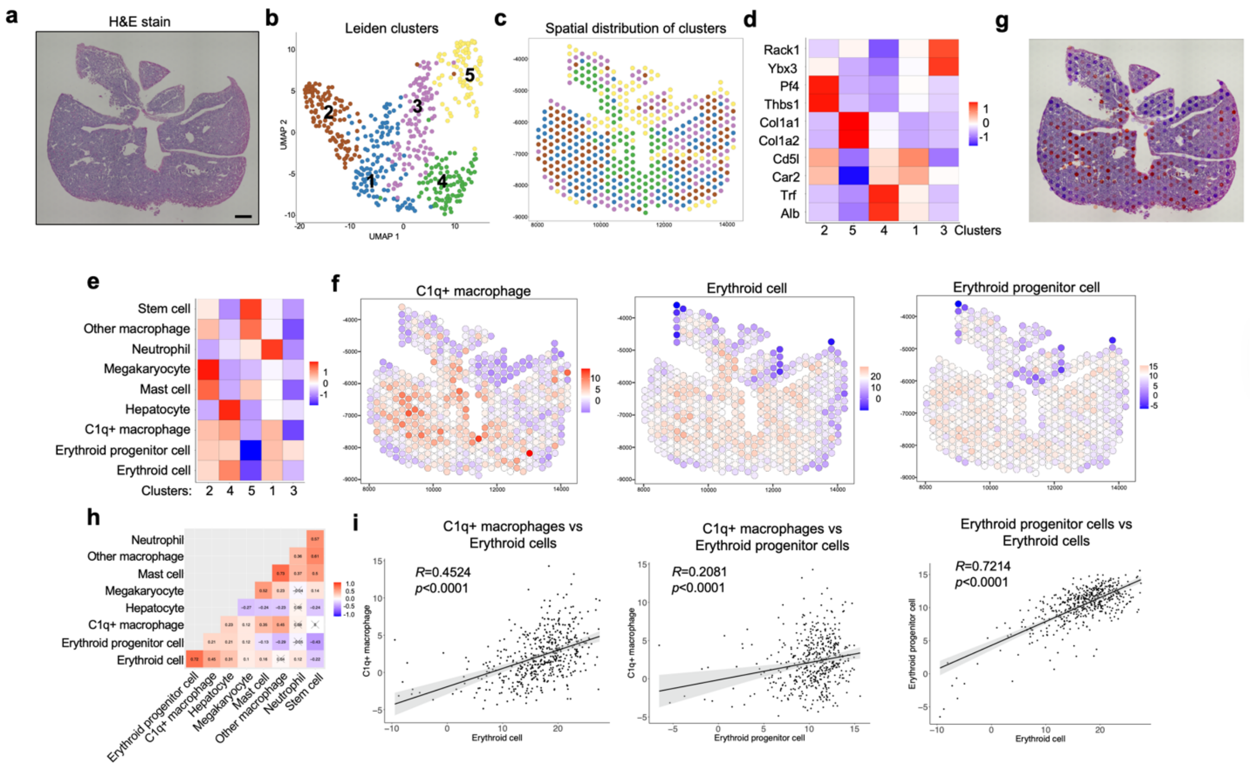

Supplementary Fig. 1. C1q is a hallmark of EBI macrophages in mouse E13.5 fetal liver. a, H&E stain of formalin-fixed paraffin-embedded E13.5 mouse fetal liver analyzed in the following spatial transcriptomic analyses. Scale bar: 300 μm. **b**, Leiden clustering of the Visium spatial transcriptomic data in a. **c**, Spatial distributions of all clusters in b. **d**, Specific marker genes of all clusters in b. **e**, Cell type enrichment heatmap of the indicated cell types in different clusters. **f**, In situ distributions of indicated cell clusters. The scale indicates the enrichment of the indicated cell type within each dot. **g**, Overlap of H&E stain with Visium spatial distribution clusters of C1q+ macrophages. **h**, Correlation R values between every two cell types. A positive R-value indicates a significant positive correlation, a negative R-value indicates a significant negative correlation, and an R-value marked with “X” indicates a non-significant correlation. **i**, Correlation analyses between the indicated two cell clusters. Each dot represents one Visium capture spot. Statistical analysis was performed using Pearson correlation (two-sided). Shaded bands represent the 95% confidence interval of the linear regression. Pearson correlation coefficient (R) and p-value are shown.

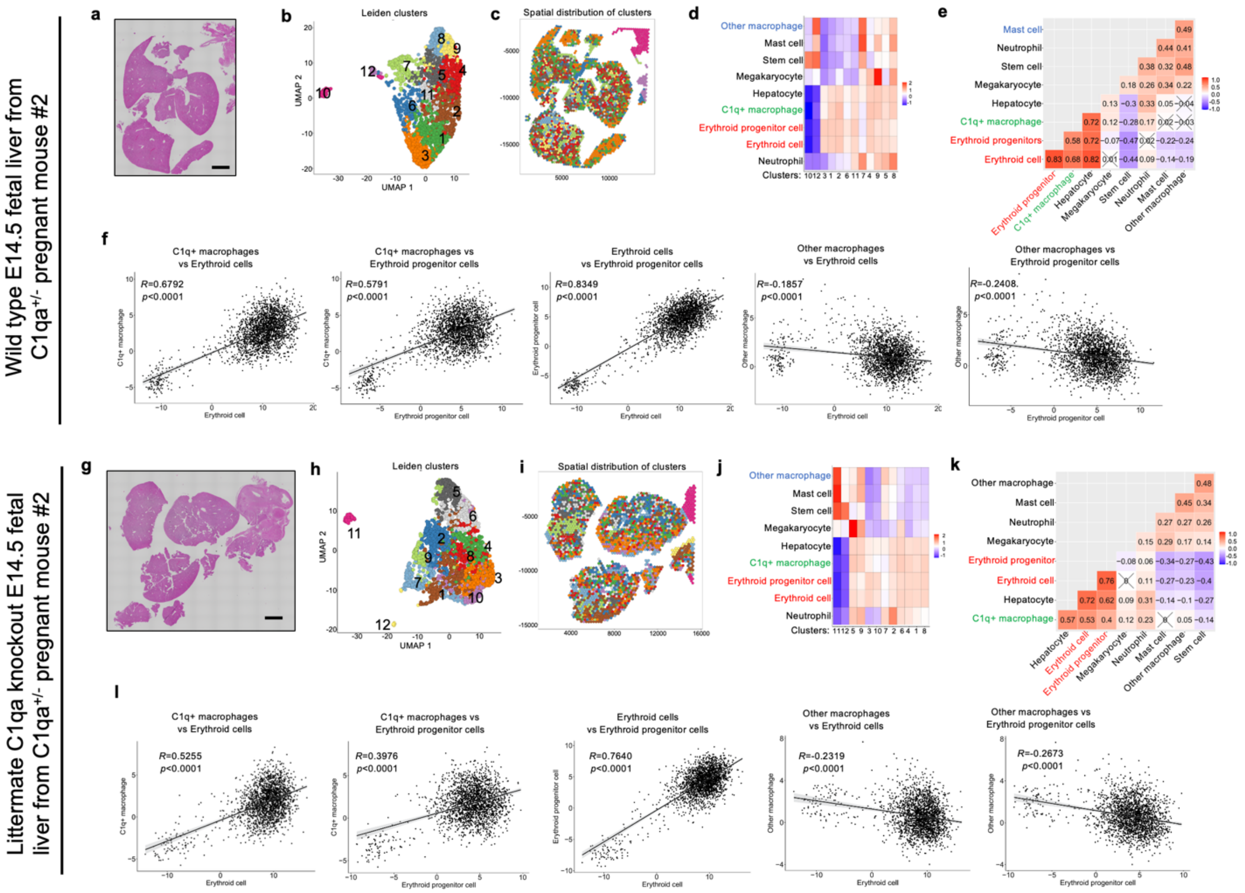

Supplementary Fig. 2. Visium spatial transcriptomic assays of E14.5 wild-type and C1qa knockout littermate mouse fetal livers from a C1qa heterozygous pregnant mouse (#2). a, H&E stain of indicated formalin-fixed paraffin-embedded E14.5 mouse fetal liver analyzed in the spatial transcriptomic analyses. Scale bar: 300 μm **b**, Leiden clustering of the Visium spatial transcriptomic data in a. **c**, Spatial distributions of all clusters in b. **d**, Cell type enrichment heatmap of the indicated cell types in different clusters. **e**, Correlation R values between every two cell types. A positive R-value indicates a significant positive correlation, a negative R-value indicates a significant negative correlation, and an R-value marked with "X" indicates a non-significant correlation. **f**, Correlation analyses between indicated different cell types. **g-l**, Same as a-f, respectively, except that the assays were done using the littermate C1qa knockout fetal liver. For f and l, each dot represents one Visium capture spot. Statistical analysis was performed using Pearson correlation (two-sided). Shaded bands represent the 95% confidence interval of the linear regression. Pearson correlation coefficient (R) and p-value are shown.

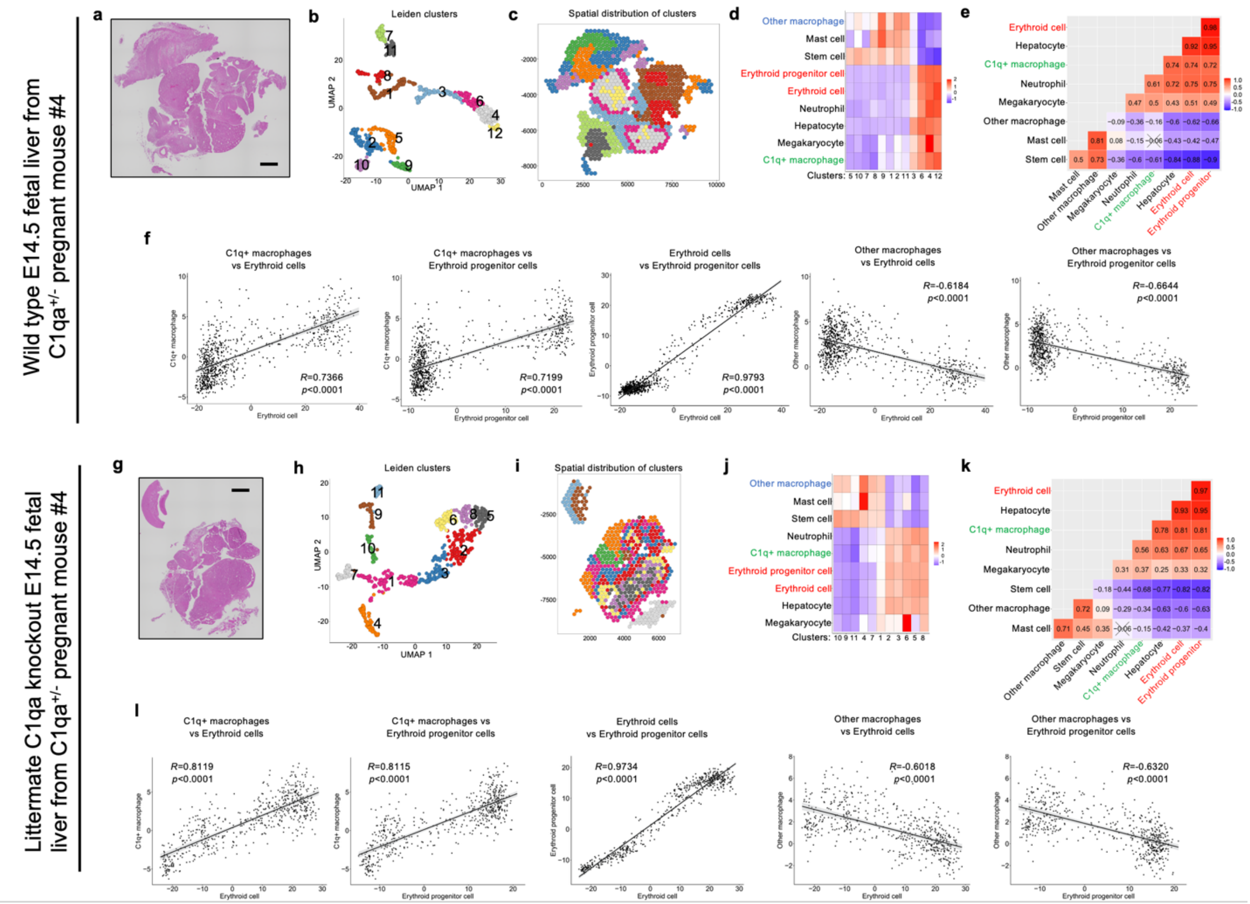

Supplementary Fig. 3. Visium spatial transcriptomic assays of E14.5 wild-type and C1qa knockout littermate mouse fetal livers from a C1qa heterozygous pregnant mouse (#4). a, H&E stain of indicated formalin-fixed paraffin-embedded E14.5 mouse fetal liver analyzed in the spatial transcriptomic analyses. Scale bar: 300 μm. **b**, Leiden clustering of the Visium spatial transcriptomic data in a. **c**, Spatial distributions of all clusters in b. **d**, Cell type enrichment heatmap of the indicated cell types in different clusters. **e**, Correlation R values between every two cell types. A positive R-value indicates a significant positive correlation, a negative R-value indicates a significant negative correlation, and an R-value marked with “X” indicates a non-significant correlation. **f**, Correlation analyses between indicated different cell types. **g-l**, Same as a-f, respectively, except that the assays were done using the littermate C1qa knockout fetal liver. For f and l, each dot represents one Visium capture spot. Statistical analysis was performed using Pearson correlation (two-sided). Shaded bands represent the 95% confidence interval of the linear regression. Pearson correlation coefficient (R) and p-value are shown.

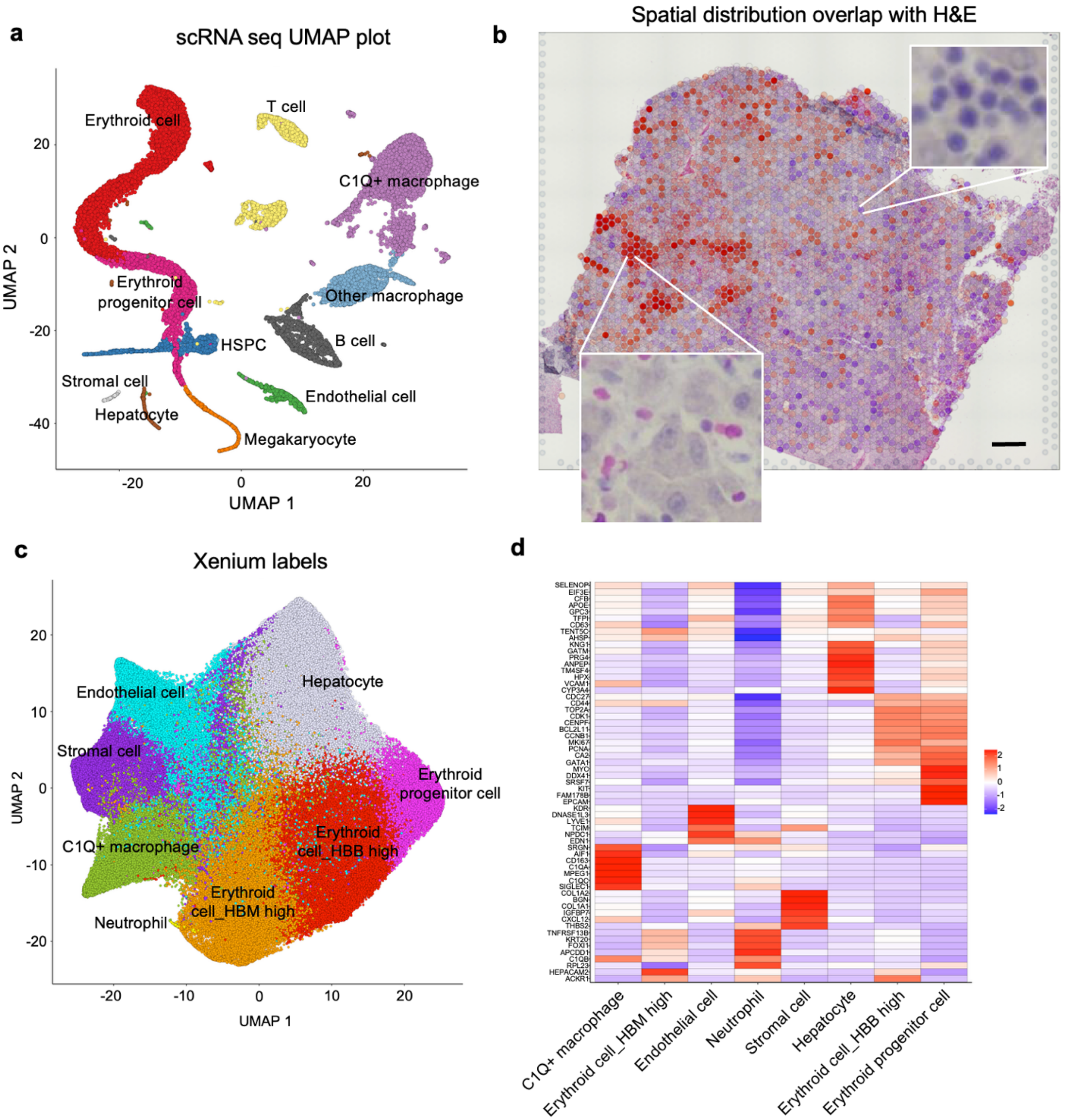

Supplementary Fig. 4. Macrophage-independent EBIs in human fetal liver. **a**, UMAP plot showing the indicated cell populations after reanalyzing single-cell RNA seq data of human 16-week gestational age fetal liver from E-MTAB-7407. **b**, Overlap of H&E stain with Visium spatial distribution clusters of C1q+ macrophages. Representative high-power H&E images of the C1q+ macrophage-enriched red clusters and less-enriched blue clusters are illustrated in the insert panels. Scale bar: 400 μm. **c**, UMAP plot derived from Xenium subcellular spatial transcriptomic data showing different cell types in human 16-week gestational age fetal liver. **d**, Specific marker genes of all cell types in c.

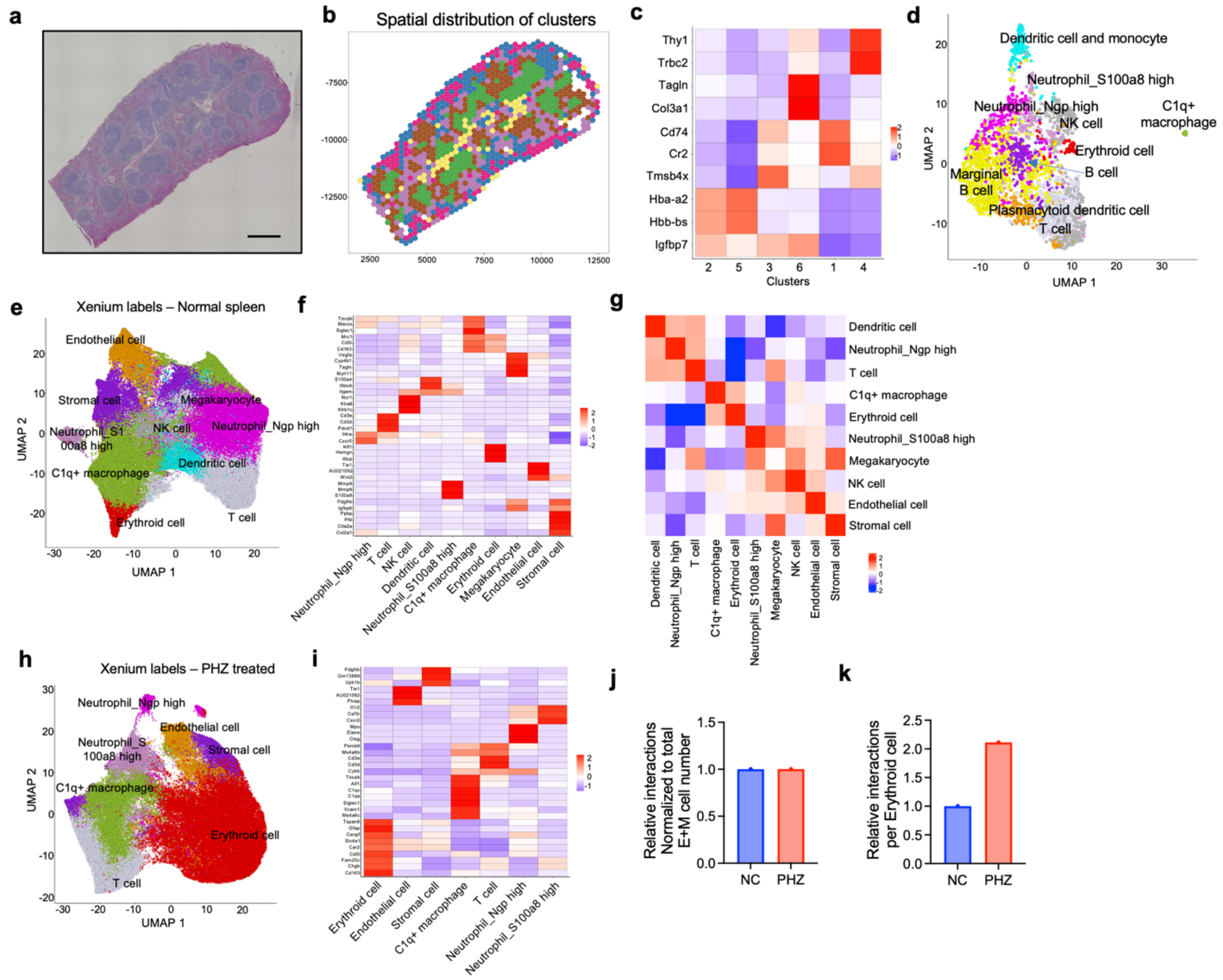

Supplementary Fig. 5. EBIs under stress in mice. a, H&E staining of formalin-fixed paraffin-embedded spleen from a 2-month-old wild-type mouse analyzed in the spatial transcriptomic analyses. Scale bar: 400 μm **b**, Spatial distributions of cell clusters derived from Visium transcriptomic assay of mouse spleen from a. **c**, Specific marker genes of all clusters in b. **d**, UMAP plot showing the indicated cell populations after reanalyzing single-cell RNA seq data from GSM2906471. **e**, UMAP plot derived from Xenium subcellular spatial transcriptomic data showing different cell types in spleen from a 2-month-old wild-type mouse. **f**, Specific marker genes of all cell types in e. **g**, A heatmap showing interactions between two cell types from the Xenium data shown in e. **h**, UMAP plot derived from Xenium subcellular spatial transcriptomic data showing different cell types in spleen from a 2-month-old wild-type mouse treated with PHZ. **i**, Specific marker genes of all cell types in h. **j**, Relative C1q+ macrophage interactions with erythroid cells after normalization to total erythroid and macrophage cell numbers in the presence or absence (NC) of PHZ. n = 1 in each group. **k**, Relative erythroid self-interactions after normalization to total erythroid and macrophage cell numbers in the presence or absence (NC) of PHZ. n = 1 in each group.

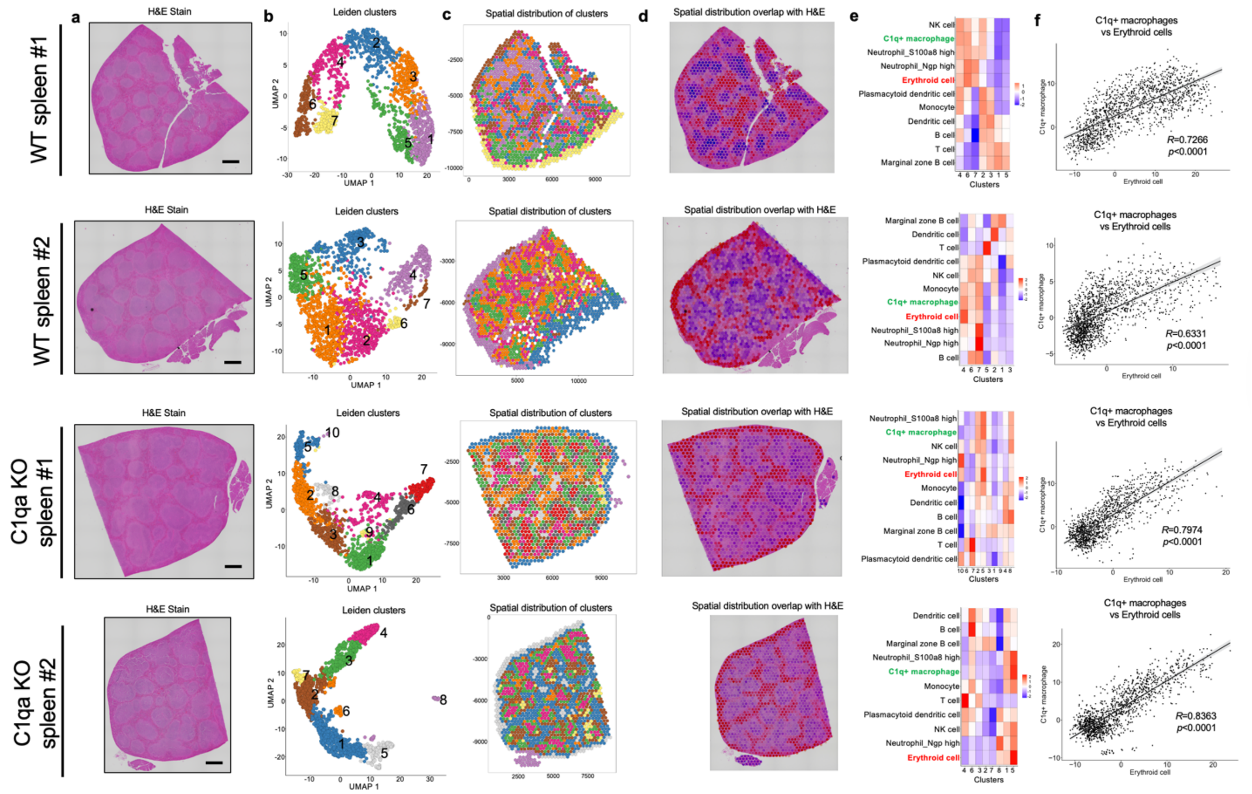

Supplementary Fig. 6. C1qa knockout does not affect the spatial distribution of C1q+ macrophage-center EBIs in the mouse spleen. From left to right columns a-f from the indicated mice: **a**, H&E stains of formalin-fixed paraffin-embedded spleens from 2-month-old mice analyzed in the spatial transcriptomic analyses. Scale bars: 300 μm **b**, Leiden clustering of the Visium spatial transcriptomic data in a. **c**, Spatial distributions of all clusters in b. **d**, Overlap of H&E stain with Visium spatial distribution clusters of C1q+ macrophages. **e**, Cell type enrichment heatmap of the indicated cell types in different clusters. **f**, Correlation analyses between indicated different cell types. Each dot represents one Visium capture spot. Statistical analysis was performed using Pearson correlation (two-sided). Shaded bands represent the 95% confidence interval of the linear regression. Pearson correlation coefficient (R) and p-value are shown.

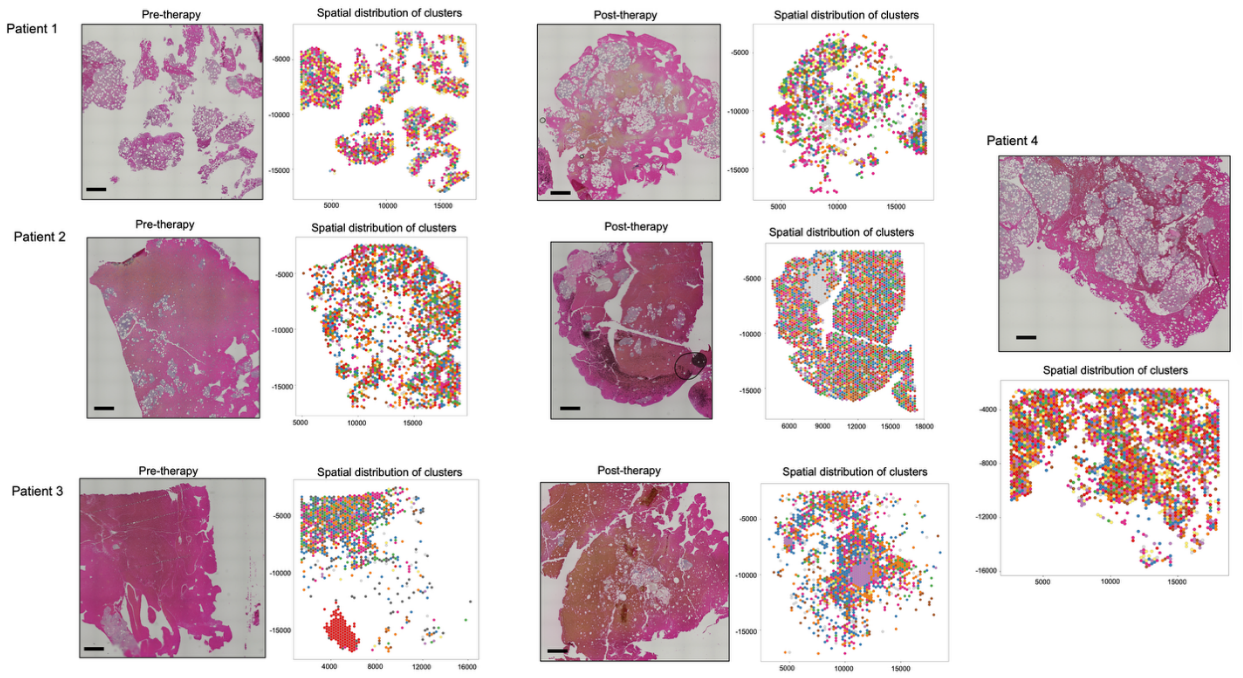

Supplementary Fig. 7. H&E stains and spatial distribution of clusters of bone marrow clot sections from patients with MDS. Specimens correspond to those in the Extended data Fig. 16b. Scale bars: 300 μm.

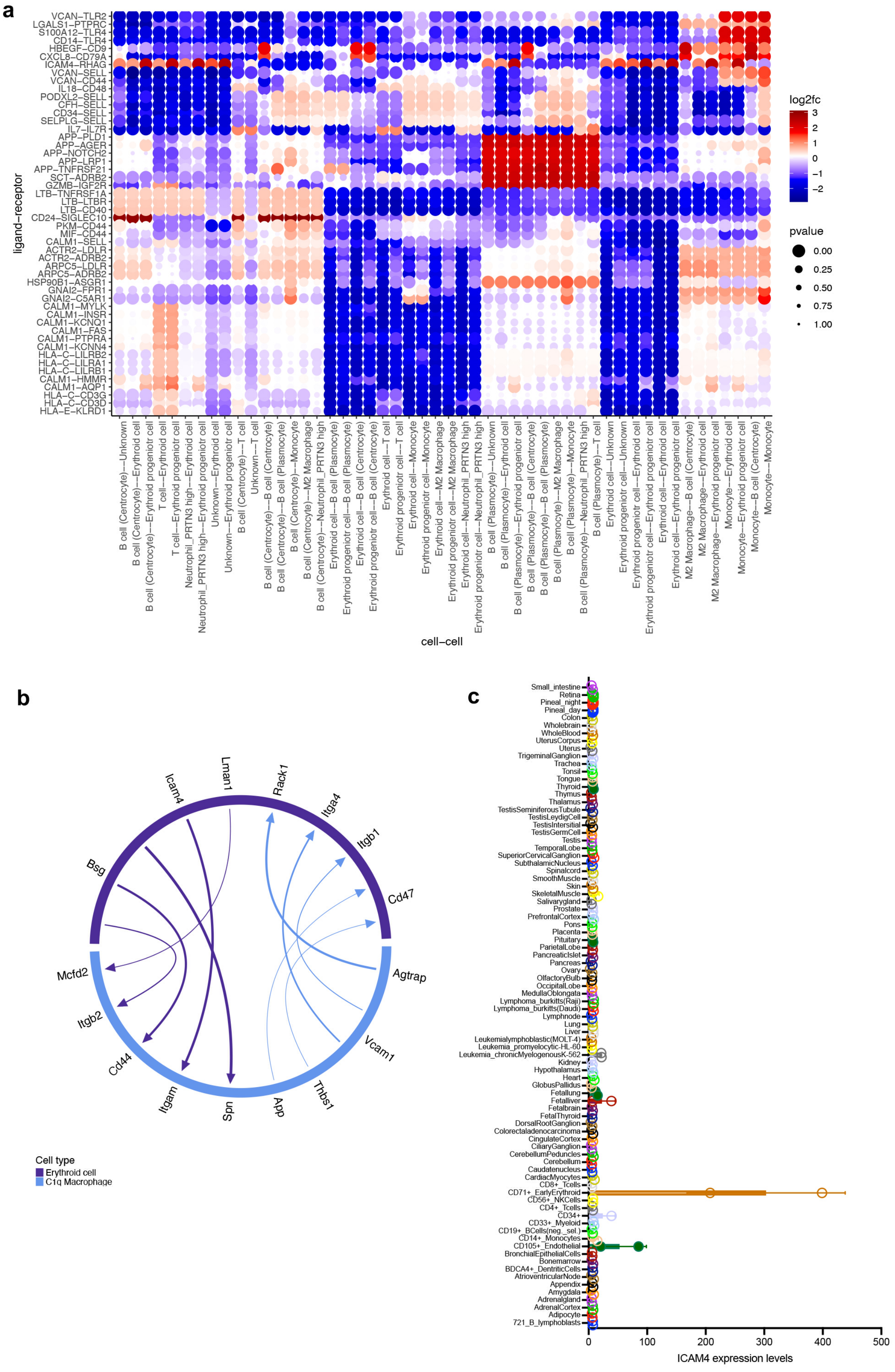
a
VCAN–TLR2
LGALS1–PTPRC
S100A12–TLR4
CD14–TLR4
HBEGF–CD9
CXCL8–CD79A
ICAM4–RHAG
VCAN–SELL
VCAN–CD44
IL18–CD48
PODXL2–SELL
CFH–SELL
CD34–SELL
SELPLG–SELL
IL7–IL7R
APP–PLD1
APP–AGER
APP–NOTCH2
APP–LRP1
APP–TNFRSF21
SCT–ADRB2
GZMB–IGF2R
LTB–TNFRSF1A
LTB–LTBR
LTB–CD40
CD24–SIGLEC10
PKM–CD44
MIF–CD44
CALM1–SELL
ACTR2–LDLR
ACTR2–ADRB2
ARPC5–LDLR
ARPC5–ADRB2
HSP90B1–ASGR1
GNAI2–FPR1
GNAI2–C5AR1
CALM1–MYLK
CALM1–INSR
CALM1–KCNQ1
CALM1–FAS
CALM1–PTPRA
CALM1–KCNN4
HLA–C–LILRB2
HLA–C–LILRA1
HLA–C–LILRB1
CALM1–HMMR
CALM1–AQP1
HLA–C–CD3G
HLA–C–CD3D
HLA–E–KLRD1
ligand–receptor
log2fc
3
2
1
0
−1
−2
pvalue
0.00
0.25
0.50
0.75
1.00
B cell (Centrocyte)---Unknown
B cell (Centrocyte)---Erythroid cell
B cell (Centrocyte)---Erythroid progeniotr cell
T cell---Erythroid cell
T cell---Erythroid progeniotr cell
Neutrophil_PRTN3 high---Erythroid cell
Neutrophil_PRTN3 high---Erythroid progeniotr cell
Unknown---Erythroid cell
Unknown---Erythroid progeniotr cell
Unknown---T cell
B cell (Centrocyte)---T cell
B cell (Centrocyte)---B cell (Centrocyte)
B cell (Centrocyte)---B cell (Plasmocyte)
B cell (Centrocyte)---Monocyte
B cell (Centrocyte)---M2 Macrophage
B cell (Centrocyte)---Neutrophil_PRTN3 high
Erythroid cell---B cell (Plasmocyte)
Erythroid progeniotr cell---B cell (Plasmocyte)
Erythroid cell---B cell (Centrocyte)
Erythroid progeniotr cell---B cell (Centrocyte)
Erythroid cell---T cell
Erythroid progeniotr cell---T cell
Erythroid cell---Monocyte
Erythroid progeniotr cell---Monocyte
Erythroid cell---M2 Macrophage
Erythroid progeniotr cell---M2 Macrophage
Erythroid cell---Neutrophil_PRTN3 high
Erythroid progeniotr cell---Neutrophil_PRTN3 high
B cell (Plasmocyte)---Unknown
B cell (Plasmocyte)---Erythroid cell
B cell (Plasmocyte)---Erythroid progeniotr cell
B cell (Plasmocyte)---B cell (Centrocyte)
B cell (Plasmocyte)---B cell (Plasmocyte)
B cell (Plasmocyte)---M2 Macrophage
B cell (Plasmocyte)---Monocyte
B cell (Plasmocyte)---Neutrophil_PRTN3 high
B cell (Plasmocyte)---T cell
Erythroid cell---Unknown
Erythroid progeniotr cell---Unknown
Erythroid progeniotr cell---Erythroid cell
Erythroid progeniotr cell---Erythroid progeniotr cell
Erythroid cell---Erythroid cell
Erythroid cell---Erythroid progeniotr cell
M2 Macrophage---B cell (Centrocyte)
M2 Macrophage---Erythroid cell
M2 Macrophage---Erythroid progeniotr cell
Monocyte---Erythroid cell
Monocyte---Erythroid progeniotr cell
Monocyte---B cell (Centrocyte)
Monocyte---Monocyte
cell–cell
b
Lman1
Icam4
Bsg
Rack1
Itga4
Itgb1
Cd47
Agtrap
Vcam1
Thbs1
App
Spn
Itgam
Cd44
Itgb2
Mcfd2
Cell type
Erythroid cell
C1q Macrophage
c
Small_intestine
Retina
Pineal_night
Pineal_day
Colon
Wholebrain
WholeBlood
UterusCorpus
Uterus
TrigeminalGanglion
Trachea
Tonsil
Tongue
Thyroid
Thymus
Thalamus
TestisSeminiferousTubule
TestisLeydigCell
TestisIntersitial
TestisGermCell
Testis
TemporalLobe
SuperiorCervicalGanglion
SubthalamicNucleus
Spinalcord
SmoothMuscle
Skin
SkeletalMuscle
Salivarygland
Prostate
PrefrontalCortex
Pons
Placenta
Pituitary
ParietalLobe
PancreaticIslet
Pancreas
Ovary
OlfactoryBulb
OccipitalLobe
MedullaOblongata
Lymphoma_burkitts(Raji)
Lymphoma_burkitts(Daudi)
Lymphnode
Lung
Liver
Leukemialymphoblastic(MOLT-4)
Leukemia_promyelocytic-HL-60
Leukemia_chronicMyelogenousK-562
Kidney
Hypothalamus
Heart
GlobusPallidus
Fetallung
Fetalliver
Fetalbrain
FetalThyroid
DorsalRootGanglion
Colorectaladenocarcinoma
CingulateCortex
CiliaryGanglion
CerebellumPeduncles
Cerebellum
Caudatenucleus
CardiacMyocytes
CD8+_Tcells
CD71+_EarlyErythroid
CD56+_NKCells
CD4+_Tcells
CD34+
CD33+_Myeloid
CD19+_BCells(neg._sel.)
CD14+_Monocytes
CD105+_Endothelial
BronchialEpithelialCells
Bonemarrow
BDCA4+_DentriticCells
AtrioventricularNode
Appendix
Amygdala
Adrenalgland
AdrenalCortex
Adipocyte
721_B_lymphoblasts
0
100
200
300
400
500
ICAM4 expression levels

Supplementary Fig. 8. ICAM4 mediates the formation of macrophage-independent EBIs in humans. **a**, A plot heatmap showing the top 50 ligand-receptor pairs mediating cell-cell interactions derived from single-cell RNA-seq data from GSM3943045 and GSM3396161 in human adult bone marrow. A permutation test was used. **b**, Circos plot showing top ligand-receptor pairs mediating erythroid cell-C1q+ macrophage interactions derived from single-cell RNA-seq data GSE176063 and GSE172127. **c**, Relative mRNA expression levels of ICAM4 in the indicated tissues or cell types in humans. The data is derived from biogps.org. n = 2 in each group.

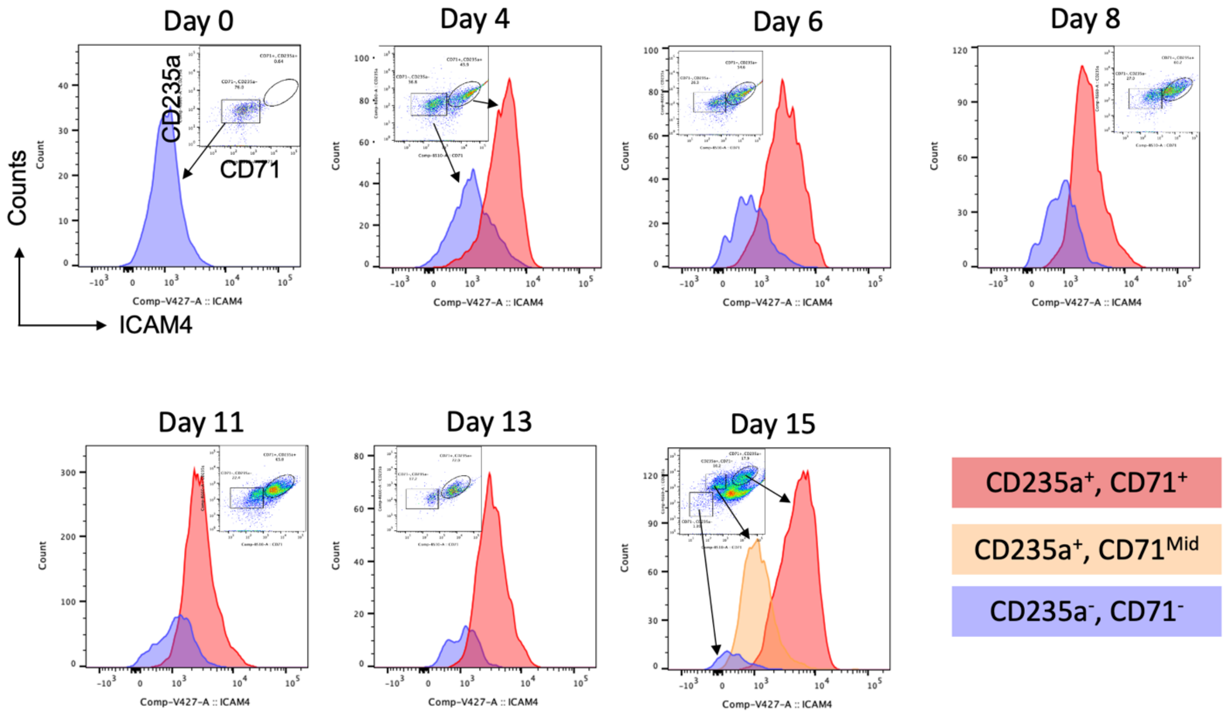

Supplementary Fig. 9. ICAM4 level changes during human terminal erythropoiesis. Flow cytometry analyses of ICAM4 expression during ex vivo human erythropoiesis. CD34+ HSPCs from healthy donor bone marrow were cultured in Epo-containing medium. ICAM4 expression levels of the gated populations were quantified at the indicated time points during culture. The inserted plots demonstrate how each gated population was selected.

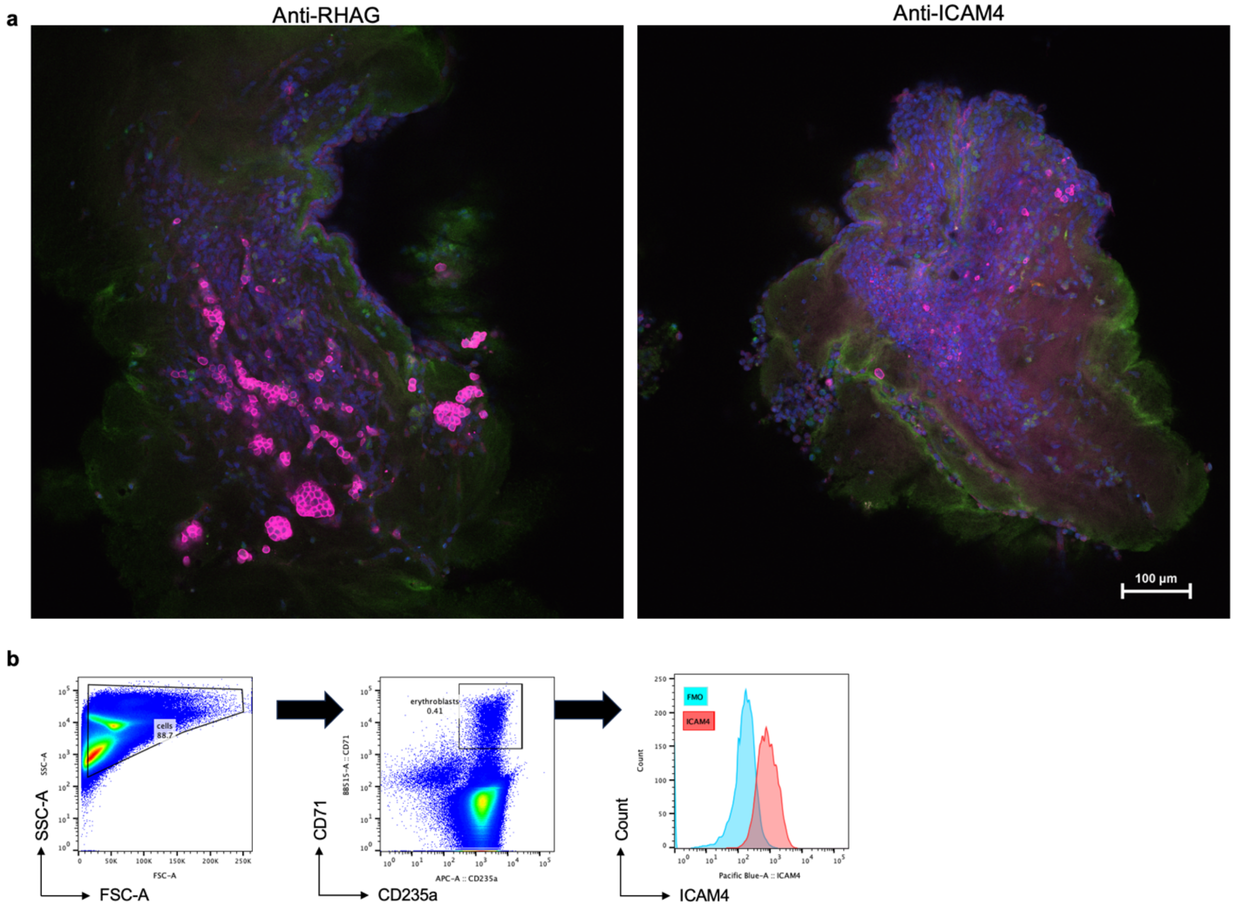

Supplementary Fig. 10. ICAM4 is involved in the formation of macrophage-independent EBIs in humans. a, Whole-mount images of human iPSC-derived bone marrow organoids stained with CD235a (magenta) and DAPI after anti-ICAM4 or anti-RHAG antibody treatment. Scale bar: 100 µm. The data represent 3 independent biological replicates. **b**, Gating strategy for the detection of ICAM4 levels in human bone marrow erythroid precursors.
