# Supplementary material for: Spatial transcriptomic analyses highlight distinct erythroid niches in mice and humans
Source: Nat Genet. 2026 Jul 2;58(7):1620–31. doi: 10.1038/s41588-026-02671-2 (PMC13364664; doi:10.1038/s41588-026-02671-2)
Supplement: Supplementary file 2 — Reporting Summary [file 41588_2026_2671_MOESM2_ESM.pdf]

Corresponding author(s): Ji

Last updated by author(s): 05/18/2026

Reporting Summary

Nature Portfolio wishes to improve the reproducibility of the work that we publish. This form provides structure for consistency and transparency in reporting. For further information on Nature Portfolio policies, see our Editorial Policies and the Editorial Policy Checklist.

Statistics

For all statistical analyses, confirm that the following items are present in the figure legend, table legend, main text, or Methods section.

n/a	Confirmed	
☐	☒	The exact sample size (n) for each experimental group/condition, given as a discrete number and unit of measurement
☐	☒	A statement on whether measurements were taken from distinct samples or whether the same sample was measured repeatedly
☐	☒	The statistical test(s) used AND whether they are one- or two-sided<br>*Only common tests should be described solely by name; describe more complex techniques in the Methods section.*
☐	☒	A description of all covariates tested
☒	☐	A description of any assumptions or corrections, such as tests of normality and adjustment for multiple comparisons
☐	☒	A full description of the statistical parameters including central tendency (e.g. means) or other basic estimates (e.g. regression coefficient) AND variation (e.g. standard deviation) or associated estimates of uncertainty (e.g. confidence intervals)
☒	☐	For null hypothesis testing, the test statistic (e.g. F, t, r) with confidence intervals, effect sizes, degrees of freedom and P value noted<br>*Give P values as exact values whenever suitable.*
☒	☐	For Bayesian analysis, information on the choice of priors and Markov chain Monte Carlo settings
☒	☐	For hierarchical and complex designs, identification of the appropriate level for tests and full reporting of outcomes
☒	☐	Estimates of effect sizes (e.g. Cohen's d, Pearson's r), indicating how they were calculated

Our web collection on statistics for biologists contains articles on many of the points above.

Software and code

Policy information about availability of computer code

Data collection	For single cell RNA sequencing data analysis, all datasets were analyzed using Giotto Suite (version 4.0.5) or Seurat (version 5.0.3) under R version 4.3.3. For the Giotto Suite and Seurat analyses, the workflows were reported on the following websites https://drieslab.github.io/Giotto_website/articles/singlecell_prostate_standard.html. https://satijalab.org/seurat/articles/pbmc3k_tutorial, respectively.<br>For the Visium data, the original fastq datasets were analyzed using Space Ranger (10X Genomics, https://www.10xgenomics.com/support/software/space-ranger/latest/tutorials/count-ffpe-tutorial). The raw feature matrix and matched spatial image files were exported for further analysis with Giotto Suite (version 4.0.5) under R version 4.3.3. The workflow of the Visium transcriptomic data analysis was previously reported.<br>For Xenium data, the original fastq datasets were analyzed by Space Ranger (10X Genomics, https://www.10xgenomics.com/support/software/space-ranger/latest/tutorials/count-ffpe-tutorial). Cell feature matrix, cell boundary, nucleus boundary, transcript files, and spatial morphology image files were exported for further analysis with Giotto Suite (version 4.0.5) under R version 4.3.3. The workflow of xenium transcriptomic data analysis was previously reported.
Data analysis	All R codes, including detailed parameters for single RNA-seq datasets, visium spatial transcriptomic datasets, and xenium spatial transcriptomic datasets, will be released after this paper is published and can be found at https://github.com/xhNorthwestern/Spatial-biology-of-hematopoiesis.

For manuscripts utilizing custom algorithms or software that are central to the research but not yet described in published literature, software must be made available to editors and reviewers. We strongly encourage code deposition in a community repository (e.g. GitHub). See the Nature Portfolio guidelines for submitting code & software for further information.

Data

Policy information about availability of data

All manuscripts must include a data availability statement. This statement should provide the following information, where applicable:

- Accession codes, unique identifiers, or web links for publicly available datasets
- A description of any restrictions on data availability
- For clinical datasets or third party data, please ensure that the statement adheres to our policy

All single-cell RNA-seq datasets used in this study were described in the methods part. All Visium spatial transcriptomic datasets and Xenium spatial transcriptomic datasets were deposited in the NCBI's Gene Expression Omnibus database (GSE271077 and GSE271150 are mouse and human visium spatial transcriptomic datasets; GSE271693 and GSE271824 are mouse and human xenium subcellular spatial transcriptomic datasets).

Research involving human participants, their data, or biological material

Policy information about studies with human participants or human data. See also policy information about sex, gender (identity/presentation), and sexual orientation and race, ethnicity and racism.

Reporting on sex and gender	*Use the terms sex (biological attribute) and gender (shaped by social and cultural circumstances) carefully in order to avoid confusing both terms. Indicate if findings apply to only one sex or gender; describe whether sex and gender were considered in study design; whether sex and/or gender was determined based on self-reporting or assigned and methods used.<br>Provide in the source data disaggregated sex and gender data, where this information has been collected, and if consent has been obtained for sharing of individual-level data; provide overall numbers in this Reporting Summary. Please state if this information has not been collected.<br>Report sex- and gender-based analyses where performed, justify reasons for lack of sex- and gender-based analysis.*
Reporting on race, ethnicity, or other socially relevant groupings	*Please specify the socially constructed or socially relevant categorization variable(s) used in your manuscript and explain why they were used. Please note that such variables should not be used as proxies for other socially constructed/relevant variables (for example, race or ethnicity should not be used as a proxy for socioeconomic status).<br>Provide clear definitions of the relevant terms used, how they were provided (by the participants/respondents, the researchers, or third parties), and the method(s) used to classify people into the different categories (e.g. self-report, census or administrative data, social media data, etc.)<br>Please provide details about how you controlled for confounding variables in your analyses.*
Population characteristics	*Describe the covariate-relevant population characteristics of the human research participants (e.g. age, genotypic information, past and current diagnosis and treatment categories). If you filled out the behavioural & social sciences study design questions and have nothing to add here, write "See above."*
Recruitment	*Describe how participants were recruited. Outline any potential self-selection bias or other biases that may be present and how these are likely to impact results.*
Ethics oversight	*Identify the organization(s) that approved the study protocol.*

Note that full information on the approval of the study protocol must also be provided in the manuscript.

Field-specific reporting

Please select the one below that is the best fit for your research. If you are not sure, read the appropriate sections before making your selection.

☒ Life sciences ☐ Behavioural & social sciences ☐ Ecological, evolutionary & environmental sciences

For a reference copy of the document with all sections, see nature.com/documents/nr-reporting-summary-flat.pdf

Life sciences study design

All studies must disclose on these points even when the disclosure is negative.

Sample size	No formal statistical power calculation was performed to predetermine sample sizes. For spatial transcriptomic experiments, sample sizes were determined based on tissue availability and are consistent with published spatial transcriptomic studies of hematopoietic tissues. For mouse experiments, a minimum of 3 biological replicates per condition was used, which is standard practice for exploratory spatial transcriptomic and single-cell analyses. For human samples, sample sizes were constrained by clinical tissue availability; a minimum of 2 biological replicates was used for spatial transcriptomic assays, with additional validation performed across independent cohorts where possible. For in vitro and in vivo functional experiments (e.g., colony-forming unit assays, transplantation experiments), a minimum of 3 biologically independent replicates per group was used, which is sufficient to detect biologically meaningful differences based on prior published studies using similar experimental systems.
Data exclusions	No data were excluded from analysis.
Replication	For mouse samples, there are 7 biological replicates for fetal liver, 6 biological replicates for spleens for Visium assays. There are 3 biological

Replication	replicates for Xenium assays. For human samples, there are 7 biological duplicates on Visium assay and 2 biological duplicates on Xenium assay. All attempts at replication were successful.
Randomization	Not applicable. This study is not a randomized trial.
Blinding	Not relevant to this study, since there was no grouping performed prior to analysis.

Reporting for specific materials, systems and methods

We require information from authors about some types of materials, experimental systems and methods used in many studies. Here, indicate whether each material, system or method listed is relevant to your study. If you are not sure if a list item applies to your research, read the appropriate section before selecting a response.

Materials & experimental systems

n/a	Involved in the study
☐	☒ Antibodies
☐	☒ Eukaryotic cell lines
☒	☐ Palaeontology and archaeology
☐	☒ Animals and other organisms
☐	☒ Clinical data
☒	☐ Dual use research of concern
☒	☐ Plants

Methods

n/a	Involved in the study
☒	☐ ChIP-seq
☐	☒ Flow cytometry
☒	☐ MRI-based neuroimaging

Antibodies

Antibodies used	FITC-conjugated anti-CD71 (BioLegend, clone CY1G4), APC-conjugated anti-CD235a (BioLegend, clone HI264), and BV421-conjugated anti-ICAM-4 (BD, clone 729632) were used in this study.<br>Primary antibodies, including TER-119 Monoclonal Antibody (14-5921-82) and C1QC/C1QG Polyclonal Antibody (BS-11337R), were purchased from Thermo Fisher Scientific. Secondary antibodies, including Mouse anti-Rat IgG2b Secondary Antibody (50-4815-82) and Goat anti-Rabbit IgG (H+L) Cross-Adsorbed Secondary Antibody (A11008), were also purchased from Thermo Fisher Scientific. IgG isotype control (Invitrogen, Cat# 02-6102), anti-human ICAM4 (Invitrogen, Cat# PA5-112917), anti-human RHAG (Invitrogen, Cat# PA5-51097), and anti-human CD51 (Invitrogen, Cat#MA5-32195) were used.
Validation	The validation of each antibody was performed by the manufacturer and related information can be found on manufacturer's website with the catalog information.

Eukaryotic cell lines

Policy information about cell lines and Sex and Gender in Research

Cell line source(s)	Human iPSC line SCTi003-A (Catalog #200-0510) derived from peripheral blood mononuclear cells (PBMCs) from a 48-year-old healthy female donor was purchased from STEMCELL Technologies.
Authentication	Demographic, health, and genetic characteristics of the SCTi003-A donor are compiled based on self-reported information and whole-exome sequencing. Sex was determined by karyotype. Ancestry and HLA haplotype were calculated from whole-genome and whole-exome sequencing combined data. Blood type (ABO/Rh blood group) was determined by next-generation sequencing. All the detailed information can be found on STEMCELL Technologies website with the catalog number.
Mycoplasma contamination	The iPSCs were routinely tested for mycoplasma contaminations negative and stable karyotype for quality control.
Commonly misidentified lines (See ICLAC register)	N/A

Animals and other research organisms

Policy information about studies involving animals; ARRIVE guidelines recommended for reporting animal research, and Sex and Gender in Research

Laboratory animals	Wild type (C57BL/6) mice used in Visium and Xenium spatial transcriptomic assays were purchased from the Jackson Laboratory. C1qa heterogeneous mice were purchased from the Jackson Laboratory, and they were crossed to obtain C1qa knockout mice.
Wild animals	N/A
Reporting on sex	Both male and female mice were used in this study.
Field-collected samples	The following timing protocol was followed for the collection of embryonic (E) stage and postnatal stage samples: One male and one

Field-collected samples	female mouse per cage were mated in the afternoon of day 1 and separated in the morning of day 2. Subsequently, all female mice were individually housed in new cages and marked as pregnant at 0.5 days post-mating.<br>For E14.5 fetal liver sample collection, post-mating 14.5 days pregnant mice were sacrificed and the fetal livers were collected. For postnatal sample collection, postnatal day 8 mice were used for bone marrow collection since the decalcification procedure in adult bone marrow degrades RNAs. For spleen collection, normal spleen was collected from wild-type mice at 3 months old. For spleen from phenylhydrazine (PHZ) treated mice, 40mg/kg PHZ or vehicle (1xPBS) were intraperitoneally injected into wild-type mice twice a day for two days. Then, PHZ-treated and control mice were sacrificed for spleen collection.<br>All the freshly collected samples were directly fixed using a 10% neutral buffered Formalin solution (Sigma-Aldrich, USA) and kept at room temperature for three days. Mouse sample embedding and sectioning were performed at Northwestern University's Mouse Histology and Phenotyping Laboratory (MHPL) facility.<br>Bone marrow transplantation using C1qa knockout mice was performed as previously reported. Briefly, total bone marrow cells were harvested from C1qa knockout mice and aged-matched wild-type mice, followed by treatment with RBC lysis buffer to delete red blood cells. The cells were then transplanted into lethally irradiated recipient mice. Two months later, peripheral blood was collected to monitor the complete blood count, and bone marrow was harvested for further analysis.
Ethics oversight	All animal studies followed the Guidelines for the Care and Use of Laboratory Animals and were approved by the Institutional Animal Care and Use Committees at Northwestern University.

Note that full information on the approval of the study protocol must also be provided in the manuscript.

Clinical data

Policy information about clinical studies

All manuscripts should comply with the ICMJE guidelines for publication of clinical research and a completed CONSORT checklist must be included with all submissions.

Clinical trial registration	N/A
Study protocol	The study protocol was approved by Northwestern University's Institutional Review Board (IRB ID: STU00217116).
Data collection	Human bone marrow aspirate samples, clot sections, and core biopsy blocks were retrospectively obtained from the Department of Pathology at Northwestern Memorial Hospital (Chicago, IL) under IRB approval (STU00217116). Samples were collected from healthy donors and patients with myelodysplastic syndromes (MDS) between 2020 and 2024. Samples were used for spatial transcriptomic, immunofluorescence, and morphological analyses.
Outcomes	This is a research study; no pre-defined primary or secondary clinical outcomes were designated. The primary scientific outcome measures were spatial transcriptomic profiling (Visium and Xenium platforms) and immunofluorescence characterization of erythroblastic island architecture in human bone marrow, including assessment of erythroid cluster composition and macrophage spatial associations in healthy donors (n=5 core biopsies; additional clot sections) and MDS patients (n=7), with paired pre- and post-therapy samples available for 3 MDS patients.

Plants

Seed stocks	*Report on the source of all seed stocks or other plant material used. If applicable, state the seed stock centre and catalogue number. If plant specimens were collected from the field, describe the collection location, date and sampling procedures.*
Novel plant genotypes	*Describe the methods by which all novel plant genotypes were produced. This includes those generated by transgenic approaches, gene editing, chemical/radiation-based mutagenesis and hybridization. For transgenic lines, describe the transformation method, the number of independent lines analyzed and the generation upon which experiments were performed. For gene-edited lines, describe the editor used, the endogenous sequence targeted for editing, the targeting guide RNA sequence (if applicable) and how the editor was applied.*
Authentication	*Describe any authentication procedures for each seed stock used or novel genotype generated. Describe any experiments used to assess the effect of a mutation and, where applicable, how potential secondary effects (e.g. second site T-DNA insertions, mosiacism, off-target gene editing) were examined.*

Flow Cytometry

Plots

Confirm that:

☒ The axis labels state the marker and fluorochrome used (e.g. CD4-FITC).

☒ The axis scales are clearly visible. Include numbers along axes only for bottom left plot of group (a 'group' is an analysis of identical markers).

☒ All plots are contour plots with outliers or pseudocolor plots.

☒ A numerical value for number of cells or percentage (with statistics) is provided.

Methodology

Sample preparation	For flow cytometry preparation, organoids were dissociated using collagenase type II (Sigma-Aldrich, cat. #C6885) dissolved in HEPES buffer (Sigma-Aldrich, cat. #H0887) to achieve a 20 mg/mL solution. The organoids were collected by sedimentation

into a 15-mL Falcon tube, rinsed twice with PBS, and then treated with the collagenase type II solution. Dissociation involved a 5-minute incubation at 37°C, followed by trituration and an additional 15-minute incubation. The dissociated organoids were then resuspended in 5 mL of PBS and filtered through a 40 µm cell strainer to obtain a single-cell suspension.
For flow analysis of human fresh bone marrow samples, fresh human bone marrow aspirate samples were obtained from leftover diagnostic specimens at Northwestern Memorial Hospital under IRB approval (IRB ID: STU00217116). Samples were diluted 1:20 in MACS buffer (PBS containing 2 mM EDTA and 0.5% BSA). A total of 100 µL of the diluted bone marrow was stained.

Instrument	BD FACSymphony A3
Software	FlowJo version 10.0
Cell population abundance	No sorting was performed.
Gating strategy	Each experiment utilized single-color stained controls and unstained controls to define positive and negative signals.

☒ Tick this box to confirm that a figure exemplifying the gating strategy is provided in the Supplementary Information.
