# Supplementary material for: Spatial transcriptomic analyses highlight distinct erythroid niches in mice and humans
Source: Nat Genet. 2026 Jul 2;58(7):1620–31. doi: 10.1038/s41588-026-02671-2 (PMC13364664; doi:10.1038/s41588-026-02671-2)
Supplement: Supplementary file 3 — Peer Review File [file 41588_2026_2671_MOESM3_ESM.pdf]

nature portfolio

Peer Review File

Spatial transcriptomic analyses highlight distinct erythroid niches in mice and humans

Corresponding Author: Dr Peng Ji

A version of this paper was originally rejected for publication by Nature Genetics, however that decision was reconsidered after appeal by the authors.

Version 0:

Decision Letter:

22nd Oct 2024

Dear Dr Ji,

Your Article entitled "Spatial transcriptomic mapping reveals distinct erythroid niches between mice and humans" has now been seen by 2 referees, whose comments are attached. In the light of their advice we have decided that we cannot offer to publish your manuscript in Nature Genetics.

While the referees find your work of some interest, they raise serious concerns about the strength of the novel conclusions that can be drawn at this stage.

We feel that these reservations are sufficiently important as to preclude publication of this study in Nature Genetics.

I am sorry that we cannot be more positive on this occasion but hope that you will find our referees' comments helpful when preparing your paper for submission elsewhere.

Sincerely,
Chiara

Chiara Anania, PhD
Associate Editor
Nature Genetics
https://orcid.org/0000-0003-1549-4157

Referee expertise:

Referee #1: erythropoiesis, epigenetics

Referee #2: quantitative spatial biology, bone marrow

Referee #3:

Reviewers' Comments:

Reviewer #1 (Remarks to the Author):

The erythroblastic island (EBI) is the established niche for erythroid differentiation in both mouse and human tissue: erythroid precursors at all stages of differentiation are attached to a central macrophage. Han et al use spatial transcriptomics to investigate the EBI. They make two principal claims: first, that in mouse, the central macrophage of the EBI

is characterized by expression of the complement factor C1q. Second, that the EBI in human tissue does not have a central macrophage, and instead is 'centered' by erythroid progenitors. The manuscript does not provide convincing evidence for either of these claims. Further, it ignores (without comment or explanation) decades of careful work from multiple authors that contradict their claims. Finally, the authors identify ICAM4 as a mediator of the human EBI interactions. However, ICAM4 is already well known to be required for EBI interactions. Unlike the model proposed by the authors, which ignore the central macrophage, previous work identified interactions between erythroblast ICAM4 and macrophage alpha V (doi:10.1016/j.tracli.2010.05.009).

The specific characteristics of the EBI macrophages are not fully known. This is a difficult area of research, since there are many macrophage subtypes that are dynamically changing, and macrophage routinely ingests some of the attached maturing erythroblasts and all extruded nuclei during the final enucleation step. It is therefore difficult to separate with confidence the transcriptomes of macrophages from those of attached erythroblasts. Here this problem is further compounded by the very low resolution of the spatial transcriptomic platform (10x Genomics Visium), where the sequencing unit integrates the transcriptomes of about 10 cells. The authors use published single-cell RNA-seq datasets to try to deduce which cell types are present in each of their spatial units. However, they provide no validation that their deconvolution is successful. Any conclusions of such an approach are therefore indirect, and would require independent validation. Their first finding, that C1q marks the central macrophage, is based on positive correlation between C1q and erythroid genes. However, C1q is also expressed in erythroblasts and red cells, and so it is possible that C1q is in fact of erythroid, not macrophage, origin. There is minimal validation of the macrophage origin for C1q in the manuscript (one fluorescent image without controls). Of note, the authors use phenylhydrazine to generate hemolytic anemia in their mice. They find the expected increase in erythroid cels but there is no associated increase in the C1q+ signal, making it unlikely that it truly or uniquely marks the EBI macrophage.

The authors proceed to investigate human EBIs. They fail to find a positive correlation between macrophage marker genes and erythroid genes in their spatial transcriptomic studies of human tissue. They do find positive correlation between cells that they term 'erythroid progenitors' and cells termed 'erythroid cells' (they do not provide a precise definition for these cell types- the marker genes used to define these terms are very limited). It is highly likely that erythroid progenitors and erythroid cells would be spatially close to each other, since erythroid cells are the progeny of erythroid progenitors, and are not particularly motile. Further, it is established that erythroid progenitors and later erythroid precursors all co-habit the same EBI units. The positive correlation between erythroid progenitors and erythroid cells is therefore expected. The claim that this supports a novel structure where erythroid progenitors are the center of the EBI and where macrophages are absent are unjustified, and the authors do not provide any independent evidence to support it. Of note, autoregulatory interactions between mature erythroblasts and erythroid progenitors within the EBI niche are already well documented (e.g. Nature volume 401, pages489–493 (1999).

The authors should consider that:
1. Only a fraction of all tissue macrophages are in the EBI. It is not clear that those in the EBI are transcriptionally distinct from those that are not. This would make finding a positive correlation between erythroid cells and macrophages very difficult.
2. The macrophage has long cytoplasmic extensions that mediate its interactions with erythroblasts. These interactions would fail to be detected in the visium platform.
3. Functional subsets of macrophages might further complicate interpretation of the low-resolution transcriptomic data from the visium platform.

Reviewer #2 (Remarks to the Author):

The manuscript by Han and colleagues represents an important step towards better understanding of the spatial organization of erythropoiesis in hematopoietic tissues, through the application of spatial transcriptomics approaches. By mapping erythroblastic islands (EBIs) in both mice and humans across different tissues and developmental stages, the study offers new insights into EBI structure and function. The authors report a distinct spatial pattern for human EBIs compared to the well-characterized mouse system, in which they have identified C1q expression as a hallmark of central macrophages. Despite its relevance, there are some concerns about the study in its current form.

Major concerns:
• The study relies on the use of bone marrow samples lacking hard bone tissue such 8 days post-natal murine bones or human bone marrow clot biopsies (Fig. 2a,b and Fig. 4a), to avoid the previously described decalcification induced RNA degradation limitation. However, recent studies have emerged illustrating the feasibility of applying spatial transcriptomics to decalcified bone samples (PMID: 37255850). Also, protocols have been developed that use EDTA-based decalcification, allowing high-quality RNA to be preserved for spatial gene expression analysis in bone tissues (PMID: 38767376). The inclusion of the bone matrix itself can provide important insights into the bone marrow microenvironment but would also better preserve bone marrow architecture for spatial analyses. Have the authors tries those protocols and could they

consider them to improve the presented tissue quality?

• Authors identify C1q as a novel marker to identify the central macrophages in EBIs and to distinguish them from “other macrophages” in mouse tissue. Previous studies (including 31101625), have identified other putative markers to identify the central macrophage including EpoR, CD169, etc. Could the authors check confirm that their C1q does not overlap with the previously reported EpoR+ macrophages at the transcriptional and protein level? IF images of C1q, EpoR, F4/80 and CD169 would be highly recommend for this.

• It would be crucial to better characterize the “other macrophage” cluster population. Could the authors quantitatively access their profile and differences from the C1q central macrophage at the transcriptional and protein level using IF?

• The C1q protein is expected to have extracellular and intracellular localization (cytoplasm). Could the authors comment on the C1q staining in Figure 1n, as the central C1q+ green structure appears to have no nucleus (DAPI) and doesn’t seem to have a macrophage ell morphology. Is this cytoplasmic or extracellular staining? In line with this, it would be beneficial if the authors could show multiple different C1q+ macrophage centered EBIs by IF imaging.

• On the same panel (Figure 1n), a cell seems to have membrane Ter119 (magenta) and polarized C1q (green) staining on its cell membrane (2 cells left from the central C1q+ objects). Could the authors explain that observation?

• The exact number of biological replicates analyzed for spatial transcriptomics is unclear in almost all figures. For instance, did the authors analyze only one section for the murine fetal liver in Figure 1 (Visium and Xenium)? If not, it would be very helpful to display their fully analyzed multiple sections in supplemental to enable assessment of sample and biological variability.

• The authors state ”A strong interaction between C1q+ macrophage and erythroid cells is evident after calculating potential interactions among different cell types based on the distance of cells derived from their spatial information, which further indicates C1q+ macrophage as the EBI macrophage” in (Fig. 1l, m). Similar statements have been made for the adult murine bone marrow (Fig. 2c, d and Fig. 2h-m). Could this be attributed to relative abundance of the C1q+ macrophages in the fetal liver tissue rather than actual correlation?

• The authors emphasize on the role C1q macrophages as a hallmark of the EBI central macrophages in mouse hematopoietic tissues throughout development. However, the study lacks functional validation of their role in vivo. It would be beneficial to include in vivo murine model with a selective knockout of C1q macrophages to investigate the effects on the EBI’s formation and erythropoiesis.

• Figure 2f: despite their efforts, the tissue integrity of the presented sample is very low which inevitably affects spatial quantification. Authors should consider removing that part or repeating with a better quality sample, maybe by following some of the recently published protocols as mentioned above (PMID: 37255850, PMID: 38767376).

• Figure 2h: those BM fragments do not look like intact bone marrow, which again unfortunately affects unbiased spatial quantification. This affects authors’ conclusions on page 8. Could the authors show some IF images of EBIs in adult mouse bone marrow to support their findings?

• Figure 3: the authors state that “Erythroid cells surrounding C1Q+ macrophages could also be observed. However, this structure was not exclusive, as other cell types were also present in proximity”. Given that the C1q+ centered EBIs are also visible, it could be that more cell types are integrated in the human setting like endothelial cells. Could the authors exclude that possibility based on their data?

• Does the spatial profiling in Figure 1a reflect a single MDS patient? If so, would it be possible for authors to increase the n number here, as a significant variability is expected among human material even within the same disease entity (particularly since MDS is quite heterogeneous). If not, could authors show the additional patient profiles in supplemental?

• The authors state that “Overall, these findings illustrate that human EBIs exhibit unique structures that are macrophage-independent and erythroid-centered”. As mentioned above, if the existence of a multi-cellular EBI cannot be excluded, authors could rephrase their conclusion.

• The authors state that “Xenium spatial studies showed continued C1q+ macrophage-centered EBIs as 3 well as markedly increased erythroid cells and their interactions (Extended Data Fig. 5h, i, Fig. 5e).”. This statement is rather qualitative and the C1q+ centered EBIs are not immediately obvious, despite the evident expansion of erythroid cells. Could the authors perform quantification in multiple sections?

• The manuscript suggests that ICAM4 plays a critical role in the formation of erythroid-centered EBIs in humans based on human bone marrow scRNA-seq dataset and functional validation on an iPSC-derived human bone marrow organoid model. To further strengthen this claim, it would be of interest to stain for the presence of ICAM4 protein on the healthy human bone marrow sample and its potential absence from the MDS human bone marrow samples.

• Given that the macrophage-erythroid cell interactions are not involved in the EBI formation in humans (through the ICAM4-integrin av axis), could the authors validate the relevant receptor-ligand pairs (shown in figure 6a) in situ and validate the corresponding cell proximity by IF imaging?

Minor concerns:
• Figure 1g/h: Can authors explain how they can have negative values on the Y axis?
• Could the authors comment on why the identified so few hepatocytes on mouse fetal liver (Extended Figure 1b)?
• Reference style is not consistent, as some references are lacking the journal's name and links (i.e. #26, 27).
• Figure 1n: what is the distance between neighboring EBIs and how many EBIs can be found in a specific tissue volume?
• Page 7: A day 8 postnatal mouse cannot be considered as "adult". Adult mouse are often 12 weeks old. Please consider revising the corresponding text.
• Several Figure panels suffer from low rendering quality which makes them a bit difficult to read.
• On page 10 authors state "In addition, Xenium 9 spatial transcriptomic data also showed a close association of cells from the erythroid lineage 10 (Fig. 4h, Extended Data Fig. 4e, f)." Could the authors show the same analysis as in 4a and 4f?
• Could the authors also stain for C1q+ in the human bone marrow?
• Figure 5d: cluster annotations are missing. The C1q+ centered EBI is not immediately visible and spatial localization seems random? Could authors statistically test this possibility?
• Figure 6d: Could authors also show a larger area (zoom out) and several zoom in areas (in the supplemental).

**Although we cannot publish your paper, it may be appropriate for another journal in the Nature Portfolio. If you wish to explore the journals and transfer your manuscript please use our manuscript transfer portal. You will not have to re-supply manuscript metadata and files, unless you wish to make modifications. For more information, please see our manuscript transfer FAQ page.

Version 1:

Decision Letter:

IMPORTANT: Please note the reference number: NG-A66466R-Z Ji. This number must be quoted whenever you communicate with us regarding this paper.

12th May 2025

Dear Dr. Ji,

Thank you for your message asking us to reconsider our decision on your manuscript "Spatial transcriptomic mapping reveals distinct erythroid niches between mice and humans". I have now discussed the points of your letter with my colleagues, and we think that you have some valid points. We therefore invite you to revise your manuscript along the lines that you propose.

When preparing a revision, please ensure that it fully complies with our editorial requirements for format and style; details can be found in the Guide to Authors on our website (http://www.nature.com/ng/).

Please be sure that your manuscript is accompanied by a separate letter detailing the changes you have made and your response to the points raised. At this stage we will need you to upload:
1) a copy of the manuscript in MS Word .docx format.
2) The Editorial Policy Checklist:
https://www.nature.com/documents/nr-editorial-policy-checklist.pdf
3) The Reporting Summary:
https://www.nature.com/documents/nr-reporting-summary.pdf
(Here you can read about the role of the Reporting Summary in reproducible science:
https://www.nature.com/news/announcement-towards-greater-reproducibility-for-life-sciences-research-in-nature-1.22062)

Please use the link below to be taken directly to the site and view and revise your manuscript:

Link Redacted

With kind wishes,
Chiara

Chiara Anania, PhD
Associate Editor
Nature Genetics
https://orcid.org/0000-0003-1549-4157

Version 2:

Decision Letter:

10th Jul 2025

Dear Dr Ji,

Your Article, "Spatial transcriptomic mapping reveals distinct erythroid niches between mice and humans" has now been seen by 3 referees. You will see from their comments copied below that while they find your work of considerable potential interest, they have raised quite substantial concerns that must be addressed. In light of these comments, we cannot accept the manuscript for publication, but would be very interested in considering a revised version that addresses these serious concerns.

We hope you will find the referees' comments useful as you decide how to proceed. If you wish to submit a substantially revised manuscript, please bear in mind that we will be reluctant to approach the referees again in the absence of major revisions.

To guide the scope of the revisions, the editors discuss the referee reports in detail within the team, including with the chief editor, with a view to identifying key priorities that should be addressed in revision and sometimes overruling referee requests that are deemed beyond the scope of the current study. In this case, we think that Reviewer #1 has valid points and we kindly ask you to address their concerns and more strongly support your conclusions regarding the novelty of the EBI structures. We also feel that the remaining requests raised by Reviewer #2 and #3 are fair and also need to be addressed in full. We hope that you will find the prioritised set of referee points to be useful when revising your study. Please do not hesitate to get in touch if you would like to discuss these issues further.

If you choose to revise your manuscript taking into account all reviewer and editor comments, please highlight all changes in the manuscript text file. At this stage we will need you to upload a copy of the manuscript in MS Word .docx or similar editable format.

We are committed to providing a fair and constructive peer-review process. Do not hesitate to contact us if there are specific requests from the reviewers that you believe are technically impossible or unlikely to yield a meaningful outcome.

If revising your manuscript:

*1) Include a “Response to referees” document detailing, point-by-point, how you addressed each referee comment. If no action was taken to address a point, you must provide a compelling argument. This response will be sent back to the referees along with the revised manuscript.

*2) If you have not done so already please begin to revise your manuscript so that it conforms to our Article format instructions, available here.
Refer also to any guidelines provided in this letter.

*3) Include a revised version of any required Reporting Summary: https://www.nature.com/documents/nr-reporting-summary.pdf
It will be available to referees (and, potentially, statisticians) to aid in their evaluation if the manuscript goes back for peer review.
A revised checklist is essential for re-review of the paper.

Please be aware of our guidelines on digital image standards.

EXTENDED DATA FIGURES

When re-submitting your manuscript, please ensure that any supplementary figures and tables that are crucial to the

manuscript's conclusions are converted into Extended Data figures and tables to increase visibility of these data. Extended Data figures and tables are online-only (present in the online PDF and full-text HTML versions of the paper), peer-reviewed display items that provide essential background to the article but are not included in the main article due to space constraints. A maximum of ten Extended Data display items (figures and tables) is permitted.

You may use the link below to submit your revised manuscript and related files:

Link Redacted

Note: This URL links to your confidential home page and associated information about manuscripts you may have submitted, or that you are reviewing for us. If you wish to forward this email to co-authors, please delete the link to your homepage.

If you wish to submit a suitably revised manuscript we would hope to receive it within 6 months. If you cannot send it within this time, please let us know. We will be happy to consider your revision so long as nothing similar has been accepted for publication at Nature Genetics or published elsewhere. Should your manuscript be substantially delayed without notifying us in advance and your article is eventually published, the received date would be that of the revised, not the original, version.

Please do not hesitate to contact me if you have any questions or would like to discuss the required revisions further.

Nature Genetics is committed to improving transparency in authorship. As part of our efforts in this direction, we are now requesting that all authors identified as 'corresponding author' on published papers create and link their Open Researcher and Contributor Identifier (ORCID) with their account on the Manuscript Tracking System (MTS), prior to acceptance. ORCID helps the scientific community achieve unambiguous attribution of all scholarly contributions. You can create and link your ORCID from the home page of the MTS by clicking on 'Modify my Springer Nature account'. For more information please visit please visit www.springernature.com/orcid.

Thank you for the opportunity to review your work.

Sincerely,
Chiara

Chiara Anania, PhD
Associate Editor
Nature Genetics
https://orcid.org/0000-0003-1549-4157

Referee expertise:

Referee #1:

Referee #2:

Referee #3: Single Cell Multi-omics/ Human Hematopoiesis

Reviewers' Comments:

Reviewer #1 (Remarks to the Author):

The authors make two claims:
1. that in mice, the EBI macrophage expresses C1q+. The authors now provide convincing evidence for this. It remains unclear however whether the C1q marker is unique to EBI macrophages, or, like many other macrophage markers, is present in both EBI and non-EBI macrophages (e.g., it's been previously shown to be present in macrophages in tumor microenvironments). The authors also fail to comment on the well-established expression of the C1q receptor by erythrocytes.

2. The authors claim that 'human EBIs exhibited a distinct architecture characterized by an erythroid-centered arrangement in the fetal liver and bone marrow' (quote from the authors' abstract). The principal argument made by the authors in their rebuttal to the critique of this claim is that the reviewer is unable to entertain novel concepts. This reviewer can assure the authors that the reason for the critique is the absence in their manuscript of rigorous and well controlled data to support their claim.

There is no data in the manuscript that shows a 'distinct' (the author's adjective) structure, detailing how erythroid progenitors and precursors are in fact arranged in the proposed new EBI. There is no data making use of conventional imaging modalities like light/fluorscence microscopy or electron microscopy, all of which have been used to identify and investigate the classical EBI. The only image provided is an H&E image in Extended Data Figure 9k, where:
• no specific cellular structure (EBI) is delineated,

• it is not clear which cell is supposed to be the central erythroid progenitor of the supposed new EBI,
• and cells are unlabeled and cannot be attributed with confidence to any lineage.

Importantly, a claim of an 'erythroid-centered arrangement' cannot be proved by spatial transcriptomic data, which is correlative. Many different types of cellular structures might give rise to the same correlations. Spatial transcriptomics therefore cannot be used to prove the existence of specific cellular structures like the EBI. It cannot show which is the 'central' cell claimed by the authors. The authors do not explain how they reached the conclusion that there is a specific structure with a central erythroid progenitor.

The authors' spatial transcriptomic data can easily be accounted for by the already known interactions between erythroid progenitors and precursors, both in the context of the classical EBI and outside it. Contrary to the authors' reply in their rebuttal, the known autoregulatory interactions between erythroid progenitors and precursors are not simply signaling interactions at a distance. They require close physical contact and membrane apposition to allow interaction of membrane proteins (like death receptors and their membrane-bound ligands).

3. The importance of ICAM4 in erythropoiesis and its expression on erythroblasts are already known. The experiments the authors show- using an antibody against ICAM4- do not prove they are disrupting an EBI structure. No EBI structures are shown in these assays.

4. While there are fewer reports and investigations of human EBIs compared with the mouse/rat/rabbit EBIs (given that model organisms are by definition more accessible), the human EBI was visualized multiple times. Examples are:

Early work from the Weatherall group showing clear detailed high-resolution images of human EBIs from surgically-removed ribs
J Exp Med (1988) 168 (3): 1193–1198.
https://doi.org/10.1084/jem.168.3.1193

More recent work, includes:

International Journal of Hematology (2021) 113:775–776
https://doi.org/10.1007/s12185-021-03147-1

haematologica 2016; 101:e178

DOI: 10.4274/tjh.galenos.2019.2019.0398 Turk J Hematol 2020;37:123-12

The authors appear to conflate two separate concepts: whether a conventional EBIs exist in humans, and whether there is a new erythroid-centered EBI in humans. Their inability to show the old structure does not take away its existence- since it's been shown by many other authors. In any case, though, absence of conventional EBIs does not prove the existence of a new one. The data they provide does not show a new structure.

The mouse data, where the authors do find the classical EBI, does not serve as a 'strong positive control' to the human, since the critical tissue harvesting and preparation steps are very different and these are critical in preserving (or disrupting) the EBI.

Of note, erythropoiesis can proceed in the absence of macrophages and EBIs. The EBI structure is supportive but not essential. This is well known in vitro, where erythroid colony formation proceeds in the absence of macrophages, and in vivo, where macrophage elimination does not prevent erythropoiesis, only prevents attaining svery high rates of erythropoiesis.

Reviewer #2 (Remarks to the Author):

Han and colleagues have substantially strengthened the manuscript by enhancing its rigor and adding complementary methods that support authors' findings. However, a number of issues raised in the original review remain insufficiently addressed in the revised version and rebuttal.

1. The authors are to be complimented for their efforts to address the relationship between C1q+ and previously reported EBI macrophage subsets, particularly for applying RNA-FISH for the in situ identification of C1q+ cells. These analyses revealed that C1q+ macrophages partially overlap with previously reported EpoR+, F4/80+, and CD169+ EBI macrophages. Could the authors demonstrate the novelty of the findings over previous studies then? The authors could consider quantifying how many F4/80+ macrophages express C1q+ in situ to confirm their flow cytometry data. Also, could the authors perform image analysis in the EpoR-tdTomato mouse to identify the overlap with C1q+ macrophages in situ and whether the C1q+ cells represent a distinct subset? Linking macrophage heterogeneity with potentially distinct spatial patterns or ideally their functional properties would be valuable. For example, are there any differences in the location or spatial neighborhood of C1q+EpoR+ versus C1q+EpoR- macrophages in the mouse fetal liver and adult bone marrow, in line with the Extended

data Fig. 6g and h?

2. In the revised manuscript version, the authors have successfully performed IF to further characterize the “other macrophage” subset and identified Tyrobp+ as a potential marker. The authors provide data for significantly fewer erythroid cells locating around “other macrophages” compared to those around C1q+ macrophages. Could the authors demonstrate the functional significance of this observation?

3. The authors should be complimented again for their efforts to increase n numbers in their spatial transcriptomic analyses. However, it is still not clear how many samples did the authors analyze from murine fetal liver and whether those came from different mice or were consecutive sections from the same mouse (technical replicates). Author should show the “H&E” and “Spatial distribution of clusters” shown in their Extended Figure 1c and 1e respectively for all the murine fetal liver samples they analyzed. In addition, authors could consider displaying the precise n number of technical and biological replicates directly in Extended Figure 1a for clarity. An n=3 (biological replicate) will be required for robust biological analysis.

4. In the original and revised version of their manuscript, the authors state a preferential localization between C1q+ macrophages and erythroid cells. Could authors explain why not all C1q+macrophages are surrounded by erythrocytes on their Figure 2g? How does this correlate with Extended data Figure 6g and h where 8-14 erythrocytes seem to surround C1q+ cells?

5. The authors performed bone marrow transplantation using total bone marrow cells from wild-type and C1qa whole-body knockout mice; however, the phenotypes are mild. Could they show whether the proximity of erythrocytes towards “other macrophages” increased in the C1qa KO mice?

6. New Extended Data Fig. 11: Could authors show the individual H&E and Spatial distribution of clusters for each analyzed patient/condition in addition to the shown spatial correlation analysis?

Reviewer #3 (Remarks to the Author):

In this manuscript, entitled ‘Spatial transcriptomic mapping reveals distinct erythroid niches between mice and humans’, Han, Ren et al., applied spatial omics to dissect erythroblast islands in mouse and human hematopoietic tissues during development and under stress conditions. In mice, the authors identified C1q as a hallmark of central macrophages in erythroblast island formation (EBI). Notably, in humans, no such central macrophages were identified, and the islands were more erythroid centered, suggesting a more self-organizing structure. This structure is further compromised in myeloid diseases. The authors further describe ICAM4 to have a critical role in maintaining human erythroid-centered islands, which is being confirmed in an iPSC-derived bone marrow organoid model.

Overall, this is a high-quality paper, which describes a quite fundamental difference in the role of macrophages vs. a self-organization of erythroblastic island formation between mice and human. The reduction of ICAM4 in the context of myelodysplastic syndrome is also interesting. The spatial transcriptomic approaches surrounding Visium and Xenium have been well-complemented by additional approaches, including microscopy and RNA-FISH to overcome challenges with respect to the relatively low resolution of the Visium platform. Nevertheless, the Visium platform could identify some of the key findings of the paper. Large parts of the paper are rather descriptive but are supported by key validating experiments.

Of note, this Reviewer was consulted only after the first round of review. Revisiting the response to the other Reviewers, the manuscript appears to have much improved over the course of the revision and this Reviewer finds the responses and discussion of revision experiments by the authors to be adequate and constructive.

Independently, this Reviewer has some additional suggestions, which would further strengthen the major conclusions of the manuscript as well as provide some additional important nuance:

Major comments
1. To further support their claims and complement the immunofluorescence images, this Reviewer would suggest leveraging the matching H&E stains to demonstrate the corresponding colocalization of C1q+ macrophages and erythroid (progenitor) cells as inferred via the spatial transcriptomics data (for Fig. 1 and 2, and Extended Data Fig. 1, 2 and 4 experiments). Essentially analogous to images shown in Extended Data Fig. 9k. Here, additional cell type labels may be helpful for the non-hemato/pathologists.

2. For the bone marrow organoid experiments in Figure 6, the authors should show CD71 vs. CD235a plots to show to what extend anti-ICAM4 treatment disrupts differentiation. Related to this and Fig. 6g-h. What does ICAM4 expression look like as a function of erythroid differentiation stage? Is it already expressed at the proerythroblast stage? What erythroblast serves as the core unit of the EBIs? Is this role being maintained by a differentiating erythroblast as it matures? These would be important aspects to discuss.

3. Related to the MDS sample analysis: in MDS, erythroid differentiation is/may be compromised, which may affect ICAM4 expression. Extended Data Fig. 13b only shows a negative/positive control staining of ICAM4, and it would be interesting to show what ICAM4 expression looks like in the MDS samples as a function of erythroid differentiation stage.

4. Can the authors quantify the average number of erythroid (progenitor) cells per EBI? Is this number similar in mice and

humans? Presumably EBIs become larger in PHZ-treated mice?

5. This Reviewer would suggest leveraging the existing data to attempt answering how these differences in EBI organization come about in mice and humans. Do erythroid and macrophages express distinct receptor-ligand levels/profiles? This may provide some additional nuance to how these species-specific differences come about. Depending on the receptor/ligand target, may be gene regulatory aspects can be interrogated (e.g., upstream transcription factor expression).

6. Are there differences known surrounding the extracellular matrix that may affect the distinct organizations in mice and human?

Minor comments
1. Can the authors clarify the following statement? “Notably, in addition to the expected complement and coagulation hallmarks, the heme metabolism pathway was also significantly enriched in C1q+ macrophage, indicating the roles of C1q+ macrophage in supporting erythropoiesis (Extended Data Fig. 3a).” How would C1q+ macrophages precisely support erythropoiesis?

2. Surrounding Figure 2 and the following statements: “Indeed, we found that the C1q protein level detected by flow cytometry was particularly high in pyrenocytes compared to erythroblasts and reticulocytes in mouse bone marrow under stress (Extended Data Fig. 7b).” and “Quantification of flow cytometry detected pyrenocytes in the 48-hour cultured erythroblasts in the presence or absence of macrophages and/or recombinant C1q.” the authors should show the matching flow plots and gating strategy.

3. Do terminally differentiated red blood cells retain or lose ICAM4 expression?

4. The authors themselves say that in vitro studies fail to replicate in vivo niches. Considering the use of a bone marrow organoid model, this should be critically discussed.

Version 3:

Decision Letter:

13th Mar 2026

Dear Dr. Ji,

Your Article, entitled "Spatial transcriptomic mapping reveals distinct erythroid niches between mice and humans", has now been seen by the 3 original referees. You will see from their comments below that while reviewers #2 and #3 are overall satisfied with the revision, reviewer #1 raises some serious concerns. We are interested in the possibility of publishing your study in Nature Genetics, but would like to consider your response to these concerns in the form of a revised manuscript before we make a final decision on publication.

We therefore invite you to revise your manuscript taking into account all reviewer comments. Please highlight all changes in the manuscript text file. At this stage we will need you to upload a copy of the manuscript in MS Word .docx or similar editable format.

We are committed to providing a fair and constructive peer-review process. Do not hesitate to contact me if there are specific requests from the reviewers that you believe are impossible to address or unlikely to yield a meaningful outcome.

When revising your manuscript:

*1) Include a “Response to referees” document detailing, point-by-point, how you addressed each referee comment. If no action was taken to address a point, you must provide a compelling argument. This response may be sent back to the referees along with the revised manuscript.

*2) If you have not done so already please begin to revise your manuscript so that it conforms to our Article format instructions, available
here.
Refer also to any guidelines provided in this letter.

*3) Include a revised version of any required Reporting Summary: https://www.nature.com/documents/nr-reporting-summary.pdf
It will be available to referees (and, potentially, statisticians) to aid in their evaluation if the manuscript goes back for peer review.
A revised checklist is essential for re-review of the paper.

Please be aware of our guidelines on digital image standards.

EXTENDED DATA FIGURES

When re-submitting your manuscript, please ensure that any supplementary figures and tables that are crucial to the manuscript's conclusions are converted into Extended Data figures and tables to increase visibility of these data. Extended Data figures and tables are online-only (present in the online PDF and full-text HTML versions of the paper), peer-reviewed display items that provide essential background to the article but are not included in the main article due to space constraints. A maximum of ten Extended Data display items (figures and tables) is permitted.

Please use the link below to submit your revised manuscript and related files:

Link Redacted

Note: This URL links to your confidential home page and associated information about manuscripts you may have submitted, or that you are reviewing for us. If you wish to forward this email to co-authors, please delete the link to your homepage.

Please do not hesitate to contact me if you have any questions or would like to discuss these revisions further.

Nature Genetics is committed to improving transparency in authorship. As part of our efforts in this direction, we are now requesting that all authors identified as 'corresponding author' on published papers create and link their Open Researcher and Contributor Identifier (ORCID) with their account on the Manuscript Tracking System (MTS), prior to acceptance. ORCID helps the scientific community achieve unambiguous attribution of all scholarly contributions. You can create and link your ORCID from the home page of the MTS by clicking on 'Modify my Springer Nature account'. For more information please visit please visit www.springernature.com/orcid.

We look forward to seeing the revised manuscript and thank you for the opportunity to review your work.

Sincerely,

Tiago

Tiago Faial, PhD
Chief Editor
Nature Genetics
https://orcid.org/0000-0003-0864-1200

Reviewers' Comments:

Reviewer #1 (Remarks to the Author):

The authors have submitted a third version of this manuscript, in which they have now gone back on their main original claims, effectively confirming that they were not justified in the first place.

The first original claim, that human erythroblastic islands are 'erythroid centered' rather than macrophage centered, was dropped, and now the authors changed it into a claim that human erythroid cells are in 'self organized clusters' without any central cell. The authors write about their previous claim that they never meant it in the first place, that 'Rather, we used "centered" in a figurative sense to convey that erythroid cells appear to aggregate independently of macrophages, forming self-organized clusters'. It seems to me that the evidence for this last claim is also very weak. Simply finding cells in clusters does not make them 'self-organized'. There is no functional data to support a claim of organization. Close apposition between erythroid cells has been noted previously (as in my prior comments, with which the authors agree). These findings are therefore not new.

The second central claim originally made by the authors was that in human tissue there are no 'conventional' erythroblastic islands in which erythroblasts surround and are attached to macrophages. The authors now agree that such structures do exist, but claim they are present at lower frequency than in mice. It is difficult to claim that this is a sufficiently novel or significant finding.

Reviewer #1 (Remarks on code availability):

I did not review the code - I have no special expertise in this.

Reviewer #2 (Remarks to the Author):

The authors have adequately addressed my comments and I have no further suggestions. The additional experiments, analyses and text edits have significantly enhanced the quality of the study.

Reviewer #3 (Remarks to the Author):

In the revised manuscript, the authors have comprehensively addressed all prior scientific comments, including through the addition of new data and revisions to previous statements to clarify their interpretation. This reviewer has no further substantive suggestions; however, minor typographical and formal issues should be checked. For example, on page 4 (bottom), gene names should be formatted in italics.

Version 4:

Decision Letter:

Our ref: NG-A66466R3

30th Apr 2026

Dear Dr. Ji,

Thank you for submitting your revised manuscript "Spatial transcriptomic mapping reveals distinct erythroid niches between mice and humans" (NG-A66466R3). It has now been seen by the original referees and their comments are below. The reviewers find that the paper has improved in revision, and therefore we'll be happy in principle to publish it in Nature Genetics, pending minor revisions to comply with our editorial and formatting guidelines.

If the current version of your manuscript is in a PDF format, please email us a copy of the file in an editable format (Microsoft Word or LaTex)-- we can not proceed with PDFs at this stage.

We are now performing detailed checks on your paper and will send you a checklist detailing our editorial and formatting requirements soon. Please do not upload the final materials and make any revisions until you receive this additional information from us.

Thank you again for your interest in Nature Genetics Please do not hesitate to contact me if you have any questions.

Congratulations!

Sincerely,
Chiara

Chiara Anania, PhD
Associate Editor
Nature Genetics
https://orcid.org/0000-0003-1549-4157

Open Access This Peer Review File is licensed under a Creative Commons Attribution 4.0 International License, which permits use, sharing, adaptation, distribution and reproduction in any medium or format, as long as you give appropriate credit to the original author(s) and the source, provide a link to the Creative Commons license, and indicate if changes were made.
In cases where reviewers are anonymous, credit should be given to 'Anonymous Referee' and the source.
The images or other third party material in this Peer Review File are included in the article's Creative Commons license, unless indicated otherwise in a credit line to the material. If material is not included in the article's Creative Commons license and your intended use is not permitted by statutory regulation or exceeds the permitted use, you will need to obtain permission directly from the copyright holder.
To view a copy of this license, visit https://creativecommons.org/licenses/by/4.0/

Dear Dr. Anania,

We would like to thank the reviewers for their valuable and insightful comments. We are particularly grateful to Reviewer #2 for their comprehensive and constructive feedback, which has significantly contributed to improving the quality and clarity of the manuscript. We also thank Reviewer #1 for their deep engagement with the work and for raising important conceptual points grounded in longstanding models in the field. While our findings challenge some of these traditional paradigms, we have taken great care to address all concerns through additional experiments, clarification of interpretations, and substantial revisions to the manuscript. In light of the reviewers' comments, we have performed additional experiments and substantially revised the manuscript. All changes are highlighted in yellow in the revised version. Please find our point-by-point responses to each comment below.

Point-by-point response:

Reviewer #1:

The erythroblastic island (EBI) is the established niche for erythroid differentiation in both mouse and human tissue: erythroid precursors at all stages of differentiation are attached to a central macrophage. Han et al use spatial transcriptomics to investigate the EBI. They make two principal claims: first, that in mouse, the central macrophage of the EBI is characterized by expression of the complement factor C1q. Second, that the EBI in human tissue does not have a central macrophage, and instead is 'centered' by erythroid progenitors. The manuscript does not provide convincing evidence for either of these claims. Further, it ignores (without comment or explanation) decades of careful work from multiple authors that contradict their claims. Finally, the authors identify ICAM4 as a mediator of the human EBI interactions. However, ICAM4 is already well known to be required for EBI interactions. Unlike the model proposed by the authors, which ignore the central macrophage, previous work identified interactions between erythroblast ICAM4 and macrophage alpha V (doi:10.1016/j.tracli.2010.05.009).

We appreciate the reviewer's feedback and recognize the importance of addressing longstanding models of erythroblastic islands (EBIs). However, we respectfully disagree with the assertion that our manuscript does not provide convincing evidence for our claims. Our study presents extensive data using spatial transcriptomics, now further supported by new immunofluorescence and RNA FISH assays, offering an unprecedented, unbiased view of EBI organization in both mouse and human tissues. If there are specific concerns about our data analysis or interpretation, we would welcome detailed feedback, as this would be more constructive than a general dismissal.

Regarding the point that we "ignore decades of careful work," we emphasize that these foundational studies, which we have explicitly and respectfully cited throughout the manuscript, are based predominantly on murine models. Our findings in mice do not contradict the existing literature but instead refine and expand upon it by highlighting a previously underappreciated C1q+ macrophage population within the EBI niche. In the revised manuscript, we now further characterize C1q+ EBI macrophages by their expression of previously reported EBI markers, including EpoR (**new Extended Data Fig. 3**).

The novelty of our study lies in the observation that, unlike in mice, human EBIs do not predominantly feature a central macrophage. Given the limited number of prior human EBI studies, our work provides much-needed spatial and functional insights that challenge conventional paradigms. We respectfully suggest that such findings be evaluated on their merits rather than dismissed based on established models derived from mouse systems.

Additionally, we fully acknowledge that ICAM4 has been previously reported to mediate interactions between erythroblasts and macrophage αV integrin (reference 40 in our manuscript). In light of this, we directly examined the involvement of αV integrin (CD51) in our study, as shown in **Fig. 6**. We conducted antibody blocking experiments in colony assays under erythroid-only differentiation conditions (Epo alone). Notably, blocking RHAG or CD51 had no effect on erythropoiesis, indicating that these molecules may not be functionally involved in human EBI formation under these conditions. To our knowledge, this represents a novel finding in human erythroid niche biology, as such experiments have not been reported previously.

We also wish to clarify what may be a misinterpretation of our findings regarding ICAM4. While prior studies demonstrated ICAM4–αV integrin interactions in murine macrophage-centered EBIs, this does not preclude a distinct role for ICAM4 in human erythroid-centered EBI structures. Our study does not dispute macrophage–erythroblast interactions in mice; rather, we present new spatial and functional evidence supporting an erythroid-centered model in human tissues. In the revised manuscript, we provide additional validation of ICAM4's role in this architecture (**new Fig. 6g–h**).

We hope the reviewer will consider these clarifications and we would be grateful for any further specific feedback that could help improve the manuscript.

The specific characteristics of the EBI macrophages are not fully known. This is a difficult area of research, since there are many macrophage subtypes that are dynamically changing, and macrophage routinely ingests some of the attached maturing erythroblasts and all extruded nuclei during the final enucleation step. It is therefore difficult to separate with confidence the transcriptomes of macrophages from those of attached erythroblasts. Here this problem is further compounded by the very low resolution of the spatial transcriptomic platform (10x Genomics Visium), where the sequencing unit integrates the transcriptomes of about 10 cells. The authors use published single-cell RNA-seq datasets to try to deduce which cell types are present in each of their spatial units. However, they provide no validation that their deconvolution is successful. Any conclusions of such an approach are therefore indirect, and would require independent validation. Their first finding, that C1q marks the central macrophage, is based on positive correlation between C1q and erythroid genes. However, C1q is also expressed in erythroblasts and red cells, and so it is possible that C1q is in fact of erythroid, not macrophage, origin. There is minimal validation of the macrophage origin for C1q in the manuscript (one fluorescent image without controls). Of note, the authors use phenylhydrazine to generate hemolytic anemia in their mice. They find the expected increase in erythroid cels but there is no associated increase in the C1q+ signal, making it unlikely that it truly or uniquely marks the EBI macrophage.

We appreciate the reviewer's insights on the complexities of studying EBI macrophages and

acknowledge the challenges involved in distinguishing macrophage transcriptomes from those of closely associated erythroblasts. We have made significant efforts to address these limitations through additional analyses and validations.

To address concerns about the resolution of the Visium platform, we supplemented our analysis with higher-resolution Xenium single-cell spatial transcriptomic data, which provides single-molecule RNA detection. In the revised manuscript, we further strengthened our findings by incorporating new data using the high-plex Xenium 5K panel, capable of detecting more than 5,000 genes (**new Extended Data Table 3, Extended Data Fig. 4, and Extended Data Fig. 5g–j**), thereby significantly enhancing detection fidelity. These data serve as independent validation of our cell-type deconvolution and confirm the presence of C1q transcripts in macrophage clusters within mouse EBIs.

In addition, we now include new immunofluorescence and RNA FISH data (**Fig. 2n and Extended Data Fig. 6**), which provide orthogonal evidence supporting the expression of C1q in macrophages in situ. Together, these complementary approaches offer robust validation of our findings.

Regarding the reviewer's comment that "C1q is also expressed in erythroblasts and red cells," we respectfully note that current literature does not support C1q expression in erythroid cells. Extensive single-cell RNA sequencing datasets from multiple tissues, including mouse fetal liver, bone marrow, and spleen, as well as human fetal liver and bone marrow [PMID: 35817981, 29474909, 32214235], consistently show C1q gene expression restricted to macrophage populations. To our knowledge, no published studies have reported C1q expression in erythroblasts or mature red blood cells. Should the reviewer be aware of such evidence, we would be grateful for any references.

In response to the comment regarding PHZ treatment: as expected, PHZ-induced hemolytic anemia led to a robust expansion of erythroid cells. C1q+ macrophages also increased, though to a lesser extent, as shown in **Fig. 5e** (please note the Xenium plot scale). Importantly, our Xenium spatial data revealed that under stress conditions, erythroid cells continued to interact with C1q+ macrophages and also formed erythroid-centered EBIs that appeared independent of macrophages. These findings suggest a plasticity in EBI organization under conditions of elevated erythropoietic demand, where erythroid self-assembly may supplement traditional macrophage-centered niches.

Rather than diminishing the role of C1q+ macrophages, these observations highlight dynamic and context-dependent adaptations of the erythroid niche, supporting the coexistence of both macrophage-dependent and -independent EBI structures in the mouse spleen.

We believe the revised manuscript now presents a comprehensive and well-validated set of findings, and we appreciate the reviewer's thoughtful feedback in refining the study.

The authors proceed to investigate human EBIs. They fail to find a positive correlation between macrophage marker genes and erythroid genes in their spatial transcriptomic studies of human tissue. They do find positive correlation between cells that they term 'erythroid progenitors' and

cells termed 'erythroid cells' (they do not provide a precise definition for these cell types- the marker genes used to define these terms are very limited). It is highly likely that erythroid progenitors and erythroid cells would be spatially close to each other, since erythroid cells are the progeny of erythroid progenitors, and are not particularly motile. Further, it is established that erythroid progenitors and later erythroid precursors all co-habit the same EBI units. The positive correlation between erythroid progenitors and erythroid cells is therefore expected. The claim that this supports a novel structure where erythroid progenitors are the center of the EBI and where macrophages are absent are unjustified, and the authors do not provide any independent evidence to support it. Of note, autoregulatory interactions between mature erythroblasts and erythroid progenitors within the EBI niche are already well documented (e.g. Nature volume 401, pages489–493 (1999).

We appreciate the reviewer's comments and the opportunity to clarify our findings. However, we find the reviewer's interpretation to be strongly aligned with the conventional macrophage-centered EBI model, without fully considering the novel aspects of our data.

First, our study does not merely claim that macrophages are absent from human EBIs. Rather, we present unbiased spatial transcriptomic data showing that erythroid progenitors, rather than macrophages, exhibit the strongest spatial correlation with differentiating erythroid cells. This is a direct observation from our data and is not a pre-conceived model. The reviewer argues that the correlation between erythroid progenitors and erythroid cells is expected due to their spatial proximity but does not address a key point—if macrophages were truly central to human EBIs, their marker genes should also show a strong correlation with erythroid genes. However, our data clearly show that this correlation is absent. If spatial proximity alone explained our findings, macrophages should exhibit the same pattern, but they do not. This inconsistency in the reviewer's reasoning suggests a preference for the traditional macrophage-centered model while not fully engaging with evidence that challenges it.

Second, we acknowledge that autoregulatory interactions between erythroblasts and erythroid progenitors have been reported previously (e.g., *Nature* volume 401, pages 489–493, 1999), and we do not dispute this. However, these studies did not establish the spatial organization of human EBIs, nor did they use spatial transcriptomic approaches to assess EBI structure comprehensively. The statement that our findings are not novel may overlook the fundamental distinction between prior work, which focused on <u>signaling interactions</u>, and our study, which provides the first spatially resolved transcriptomic evidence suggesting an alternative EBI organization in human tissue.

Furthermore, the reviewer's statement that we use a "limited set of marker genes" to define erythroid progenitors and erythroid cells is an oversimplification. Our classification is based on well-established marker genes for erythroid differentiation, which are routinely used in both experimental and clinical hematology. These markers were reported based on previously published scRNA-seq datasets (reference 32-33, now with a complete marker gene list in **new Extended Data Tables 1 and 5**), ensuring their validity in identifying distinct erythroid populations. We welcome suggestions for additional markers that the reviewer believes would improve cell-type classification. However, simply stating that our definitions are inadequate without specifying what is lacking does not constitute a substantive critique.

Finally, we emphasize that our conclusions are supported by multiple lines of independent evidence, including Xenium single-cell spatial transcriptomics, immunofluorescence, and ICAM4 functional data, all of which further validate the organization of human EBIs as revealed by our spatial transcriptomics. We encourage the reviewer to consider these data rather than discounting them based on pre-existing models derived from murine studies.

The authors should consider that:
1. Only a fraction of all tissue macrophages are in the EBI. It is not clear that those in the EBI are transcriptionally distinct from those that are not. This would make finding a positive correlation between erythroid cells and macrophages very difficult.
2. The macrophage has long cytoplasmic extensions that mediate its interactions with erythroblasts. These interactions would fail to be detected in the visium platform.
3. Functional subsets of macrophages might further complicate interpretation of the low-resolution transcriptomic data from the visium platform.

We appreciate the reviewer's thoughtful considerations and agree that the complexity of tissue macrophage populations poses challenges in studying erythroblastic islands (EBIs). We would like to clarify a few key points regarding our approach and findings.

1. We fully acknowledge that only a subset of macrophages participate in EBIs and that transcriptional distinctions between EBI-associated and non-EBI macrophages may not always be clear. Nonetheless, we have successfully detected macrophage-centered EBI structures in murine hematopoietic tissues, providing a robust positive control for our spatial transcriptomic analyses (Visium and Xenium) and demonstrating their capacity to detect such cellular interactions. If macrophages played a predominant role in human EBI organization, we would still expect to observe a measurable correlation between macrophage markers and erythroid genes. The absence of such correlation in human tissue, despite being observed in mouse models, suggests a fundamental difference in EBI organization rather than a technical limitation in detecting macrophage heterogeneity.

2. We agree that long cytoplasmic extensions of macrophages may be difficult to capture using Visium due to its resolution constraints. However, despite these cytoplasmic extensions (if present), we successfully detected macrophage-centered EBI structures in murine hematopoietic tissues, indicating that such morphological features do not hinder the ability of unbiased spatial transcriptomic analyses to identify these interactions. To further address this, we complemented our Visium data with higher-resolution Xenium single-cell spatial transcriptomics, which allows for finer spatial mapping of gene expression at the sub-cellular level. The Xenium studies did not reveal macrophage cytoplasmic extensions interacting with erythroblasts in mice or humans. In addition, our new immunofluorescence plus RNA FISH analysis did not reveal macrophage cytoplasmic extensions around erythroid clusters either (**new Fig. 2n and Extended Data Fig. 6**).

3. While macrophage functional heterogeneity is well documented, the notion that Visium resolution is insufficient to detect relevant macrophage subpopulations assumes these subsets are transcriptionally distinct in a manner that would be missed. To mitigate this concern, we integrated multiple modalities, including matched scRNA-seq references, immunofluorescence,

and RNA FISH, to ensure robust identification of macrophage-associated signals. If macrophages were central to human EBI architecture, we would expect consistent spatial proximity and transcriptional correlation across these platforms, as we observe in mice. However, this pattern is not seen in human tissues.

We appreciate the reviewer's insights and hope these clarifications provide a clearer understanding of our integrated approach. Taken together, our findings suggest species-specific differences in EBI structure that merit further investigation and interpretation beyond classical macrophage-centered models.

Reviewer #2:

Major concerns:

• The study relies on the use of bone marrow samples lacking hard bone tissue such 8 days post-natal murine bones or human bone marrow clot biopsies (Fig. 2a,b and Fig. 4a), to avoid the previously described decalcification induced RNA degradation limitation. However, recent studies have emerged illustrating the feasibility of applying spatial transcriptomics to decalcified bone samples (PMID: 37255850). Also, protocols have been developed that use EDTA-based decalcification, allowing high-quality RNA to be preserved for spatial gene expression analysis in bone tissues (PMID: 38767376). The inclusion of the bone matrix itself can provide important insights into the bone marrow microenvironment but would also better preserve bone marrow architecture for spatial analyses. Have the authors tries those protocols and could they consider them to improve the presented tissue quality?

We appreciate the reviewer's insightful comments and fully agree that preserving bone marrow architecture while maintaining RNA integrity is crucial for spatial transcriptomic analysis. Based on the reviewer's suggestion, we attempted EDTA-based decalcification, same as what was described in the published paper (PMID: 38767376), followed by Visium spatial transcriptomics on murine bone marrow. To ensure data quality, we applied standard filtering criteria: gene expression threshold = 1, genes detected in a minimum of 50 cells, and a minimum of 200 detected genes per cell. However, we observed that only a small fraction of spots (approximately 15%) and genes (less than 10%) passed these filters with the EDTA method, leading to poor data quality (See data below).

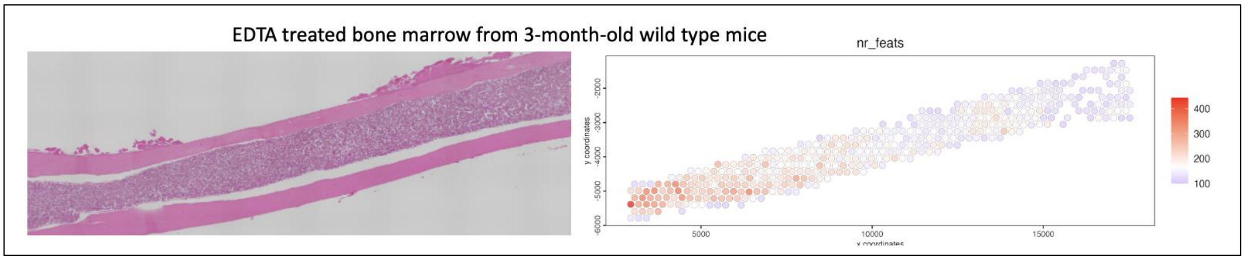

The published report (PMID: 38767376) only contains a procedure and quality analysis. <u>There were no real spatial transcriptomic studies performed in this paper</u>. We did not find published

spatial transcriptomic data from the same research group. To further assess the impact of the EDTA-based method on spatial transcriptomics, we reanalyzed published Visium data from a different group using EDTA-decalcified adult mouse leg bone (PMID: 37926705). Our analysis showed similar results, with significant mRNA degradation affecting gene detection and overall data quality (See data below on the left). These findings suggest that despite EDTA's milder decalcification properties, it still compromises RNA integrity to an extent that makes Visium-based spatial transcriptomics unreliable. In contrast, the 8-day postnatal murine bones and cryo-sectioned human bone marrow clot biopsies used in our study yielded high-quality data (See data below on the right, note the differences in scales), with the majority of cells and genes passing filtering thresholds and enabling successful downstream analysis (see **new Extended Data Fig. 5k** for quality comparison).

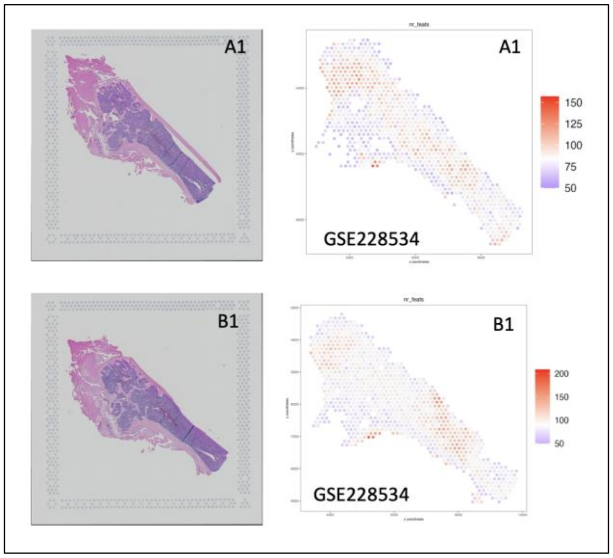

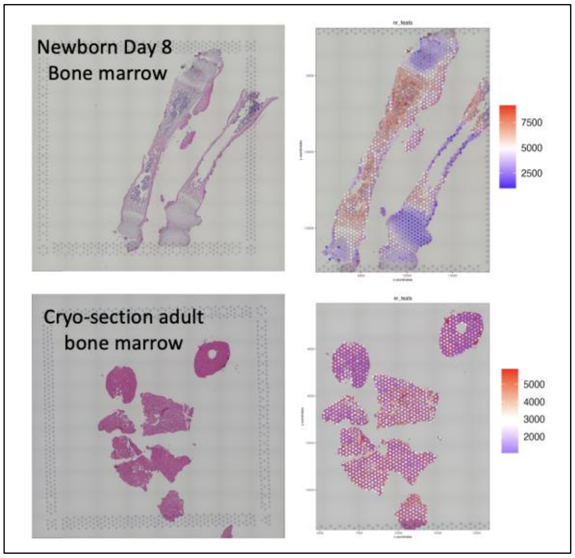

Regarding the study in PMID: 37255850, we note that the authors used the Nanostring GeoMx Digital Spatial Profiling platform. While GeoMx provides valuable spatial transcriptomic insights, it has several limitations compared to Visium and Xenium. Specifically, GeoMx relies on targeted gene panels rather than whole-transcriptome profiling, limiting the discovery of novel spatial gene expression patterns. Additionally, its resolution is significantly lower than that of Xenium, making it less suitable for high-resolution mapping of niche structures such as erythroblastic islands. Furthermore, GeoMx requires predefined regions of interest, whereas Visium and Xenium capture unbiased spatial gene expression across entire tissue sections. Given these limitations, the findings from GeoMx studies may not be directly comparable to our whole-transcriptome, high-resolution spatial analysis.

We appreciate the reviewer's suggestion and acknowledge the potential of optimized decalcification methods in spatial transcriptomics. However, based on our own experimental results and reanalysis of published datasets, we find that EDTA-based decalcification significantly compromises RNA quality for Visium analysis. We believe our approach using postnatal murine bones and cryo-sectioned murine bone marrow provides the best balance between preserving spatial architecture and maintaining transcriptomic integrity.

• Authors identify C1q as a novel marker to identify the central macrophages in EBIs and to distinguish them from "other macrophages" in mouse tissue. Previous studies (including 31101625), have identified other putative markers to identify the central macrophage including EpoR, CD169, etc. Could the authors check confirm that their C1q does not overlap with the previously reported EpoR+ macrophages at the transcriptional and protein level? IF images of C1q, EpoR, F4/80 and CD169 would be highly recommend for this.

We appreciate the reviewer's insightful suggestion and have performed additional analyses to clarify the relationship between C1q+ macrophages and previously reported EpoR+ EBI macrophages.

1. **Transcriptomic Analysis:** We examined the published dataset from the study identifying EpoR+ EBI macrophages (PMID: 31101625) and found that all three C1q family members (*C1qa, C1qb, C1qc*) were significantly upregulated in *F4/80+EpoR+* macrophages compared to *F4/80+EpoR-* macrophages (**new Extended Data Fig. 3b**). To further validate this, we obtained bone marrow from *EpoR-tdTomato* transgenic mice and sorted *F4/80+EpoR+* and *F4/80+EpoR-* macrophages following the methods described in the original study (PMID: 31101625). Quantitative PCR (qPCR) analysis confirmed that *C1qb* and *C1qc* expression levels were significantly higher in *F4/80+EpoR+* macrophages than in *F4/80+EpoR-* macrophages (**new Extended Data Fig. 3c**).

2. **Flow Cytometry Analysis:** To further characterize *F4/80+C1q+* macrophages, we performed flow cytometry assays to simultaneously assess CD163, CD169, CD11b, F4/80, and C1q expression. Our results showed that *F4/80+C1q+* macrophages exhibited higher CD163 and CD169 expression and lower CD11b expression compared to *F4/80+C1q-* macrophages (**new Extended Data Fig. 3d, e**). In the bone marrow from *EpoR-tdTomato* transgenic mice, flow cytometry assay revealed that *EpoR* protein levels were not significantly different between *F4/80+C1q+* and *F4/80+C1q-* macrophages, suggesting that while C1q+ macrophages may overlap with EpoR+ macrophages, they are not exclusively defined by EpoR expression (**new Extended Data Fig. 3f**).

3. **Imaging study:** We performed a traditional immunofluorescence assay of C1q, Ter119, and F4/80, which revealed a dotted and diffuse expression pattern of C1q, consistent with the secreted nature of the protein (**new Extended Data Fig. 6a**). Recognizing this limitation, we further performed immunofluorescence (IF) combined with RNA fluorescence in situ hybridization (RNA-FISH). These analyses revealed that *C1qa* mRNA+ macrophages form EBI structures with Ter119+ erythroid cells in mouse fetal liver and bone marrow (**new Fig. 2n and Extended Data Fig. 6b-c**). Additionally, *C1qa* mRNA staining showed partial colocalization with F4/80 staining, further confirming the macrophage identity of C1q-expressing cells (**new Extended Data Fig. 6d**).

Based on these findings, we conclude that C1q+ macrophages partially overlap with previously reported EpoR+, F4/80+, and CD169+ EBI macrophages, reinforcing their role in EBI formation while also highlighting potential heterogeneity within this macrophage population. We appreciate the reviewer's suggestion, which has helped further clarify the transcriptional and protein-level relationships between these macrophage subtypes.

• It would be crucial to better characterize the "other macrophage" cluster population. Could the authors quantitatively access their profile and differences from the C1q central macrophage at the transcriptional and protein level using IF?

We have already provided transcriptomic differences between *C1q*+ macrophages and other macrophages in **Fig. 1j and 1k**, and demonstrated their distinct gene expression profiles. To further validate these findings at the protein level, we performed additional experiments using IF. Given that C1q is a secreted protein, traditional IF staining resulted in a non-specific diffuse pattern (**new Extended Data Fig. 6a**). Therefore, we used RNA-FISH to specifically detect *C1qa* mRNA, which ensures precise localization of *C1q*+ macrophages within the EBI structure (**new Fig. 2n and Extended Data Fig. 6b-c**). We also identified *Tyrobp*+ other macrophages by IF. Both *C1q*+ and *Tyrobp*+ macrophages express F4/80 (**new Extended Data Fig. 6d**). Furthermore, we also performed RNA FISH on Tyrobp and found rare or no erythroblasts surrounding Tyrobp+ macrophages in mice (**new Extended Data Fig. 6e-f**). The quantification of erythroblasts surrounding macrophages indeed demonstrated significantly fewer erythroid cells around other macrophages than those around C1q+ macrophages (**new Extended Data Fig. 6g-h**). These additional data further confirm the distinct roles of *C1q*+ macrophages in EBI formation compared to other macrophages within the bone marrow environment.

We appreciate the reviewer's insightful comment, which has helped us strengthen the characterization of these macrophage subpopulations.

• The C1q protein is expected to have extracellular and intracellular localization (cytoplasm). Could the authors comment on the C1q staining in Figure 1n, as the central C1q+ green structure appears to have no nucleus (DAPI) and doesn't seem to have a macrophage cell morphology. Is this cytoplasmic or extracellular staining? In line with this, it would be beneficial if the authors could show multiple different C1q+ macrophage centered EBIs by IF imaging.

As C1q is a secreted protein, it can localize both intracellularly and extracellularly (in the surrounding microenvironment, including binding to erythroid cells). The green C1q+ structure in old Figure 1n likely represents intracellular C1q. The absence of a visible DAPI+ nucleus in the same plane could be due to the nucleus being slightly above or below the imaging focal plane, rather than an indication that the structure lacks a nucleus.

Old Figure 1n also exhibited dotted C1q+ signals throughout the imaging focal plane. This is consistent with previous reports that C1q proteins can diffuse and bind to various cell surfaces, particularly erythroid cells, which we also observed in our new IF staining (**new Extended Data Fig. 6a**) that replaced old Figure 1n. We also found that the extruded nuclei (pyrenocytes) exhibited the highest C1q surface levels compared to reticulocytes or erythroblasts (**new Extended Data Fig. 7b**).

To improve specificity and avoid the non-specific, diffuse staining of C1q, we have included new images in **new Fig. 2n and Extended Fig. 6b-c** using RNA-FISH for *C1qa* combined with IF staining. This approach clearly revealed multiple *C1q*+ macrophages surrounded by maturing

erythroblasts, confirming their role in EBI structures. Additionally, these data acknowledge that extracellular C1q staining may occur due to its secreted nature.

We appreciate the reviewer's insightful comment, which has helped us further refine our imaging and interpretation.

• On the same panel (Figure 1n), a cell seems to have membrane Ter119 (magenta) and polarized C1q (green) staining on its cell membrane (2 cells left from the central C1q+ objects). Could the authors explain that observation?

This cell is most likely an erythroblast with both Ter119 and C1q staining, consistent with the nature of C1q as a secreted protein binding to erythroblasts, as we discussed above.

• The exact number of biological replicates analyzed for spatial transcriptomics is unclear in almost all figures. For instance, did the authors analyze only one section for the murine fetal liver in Figure 1 (Visium and Xenium)? If not, it would be very helpful to display their fully analyzed multiple sections in supplemental to enable assessment of sample and biological variability.

We thank the reviewer for this valuable suggestion, which has helped strengthen the rigor of our study. In the original manuscript, we analyzed one section of the murine fetal liver for both Visium and Xenium spatial transcriptomics. However, we performed multiple (4) human bone marrow clot section spatial transcriptomic studies in normal individuals (**new Extended Data Fig 9f-i**) and 7 MDS patients (**new Extended Data Fig. 11**). In the revised version, we have repeated both Visium and Xenium assays on additional murine fetal liver and human bone marrow samples to ensure consistency of our findings. The data from these repeated samples are now included in **new Extended Data Fig. 1c-h and Extended Data Fig. 2** for mice, and **new Extended Data Fig. 9b-e** for humans, and they show similar results as the respective replicates, reinforcing the reproducibility of our conclusions.

• The authors state "A strong interaction between C1q+ macrophage and erythroid cells is evident after calculating potential interactions among different cell types based on the distance of cells derived from their spatial information, which further indicates C1q+ macrophage as the EBI macrophage" in (Fig. 1l, m). Similar statements have been made for the adult murine bone marrow (Fig. 2c, d and Fig. 2h-m). Could this be attributed to relative abundance of the C1q+ macrophages in the fetal liver tissue rather than actual correlation?

We appreciate the reviewer's insightful question regarding whether the observed interaction between C1q+ macrophages and erythroid cells could be attributed to their relative abundance rather than a true correlation. While C1q+ macrophages are present in the fetal liver, their spatial association with erythroid cells is not solely a result of abundance. In **Figs. 1l, 1m, 2c, 2d, and 2h-m**, we performed spatial interaction analysis based on cell-type distances, which quantitatively assesses how closely different cell populations associate beyond random distribution. If C1q+ macrophages were simply abundant without specific interactions, we would expect a more uniform spatial relationship with multiple cell types, rather than a preferential association with erythroid cells.

Additionally, single-cell RNA sequencing data from mouse fetal liver revealed that "other macrophages" are actually more abundant than C1q+ macrophages (**Extended Data Fig. 1b**). However, their correlation index with erythroid cells was lower than that of C1q+ macrophages, further confirming that the observed interaction is not driven by relative abundance but reflects a biologically meaningful relationship.

We have already presented data in **Figs. 1i, 1n**, **and new Extended Data Fig. 1h**, where we analyzed cell-cell interactions normalized for overall cell-type abundance. These results consistently show a strong preferential association between C1q+ macrophages and erythroid cells, reinforcing their role as EBI macrophages.

• *The authors emphasize on the role C1q macrophages as a hallmark of the EBI central macrophages in mouse hematopoietic tissues throughout development. However, the study lacks functional validation of their role in vivo. It would be beneficial to include in vivo murine model with a selective knockout of C1q macrophages to investigate the effects on the EBI's formation and erythropoiesis.*

We appreciate the reviewer's suggestion regarding the in vivo functional validation of C1q+ macrophages in EBI formation and erythropoiesis. To address this, we performed bone marrow transplantation using total bone marrow cells from wild-type and C1qa whole-body knockout mice. The results showed that C1qa knockout led to mild anemia, demonstrating its role in erythropoiesis (**new Extended Data Fig. 7e**). Importantly, C1qa knockout also resulted in a higher percentage of pyrenocytes in the bone marrow, indicating that C1q promotes nuclear engulfment during terminal erythropoiesis (**new Fig. 2p**).

To confirm the role of C1q and rule out potential compensatory effects in the in vivo assay, we conducted co-culture experiments of macrophages and erythroblasts with or without recombinant C1q treatment. We found that pyrenocytes were significantly reduced when the erythroblasts were co-cultured with macrophages together with recombinant C1q, which demonstrates that C1q+ is involved in mediating macrophage clearance of the extruded nuclei (**new Fig. 2o, new Extended Data Fig. 7c-d**). These data reinforce that C1q plays a direct role in EBI function.

These results provide functional evidence supporting our findings that C1q+ macrophages contribute to EBI formation and erythropoiesis. We appreciate the reviewer's valuable suggestion and have incorporated these new data in the revised manuscript.

• *Figure 2f: despite their efforts, the tissue integrity of the presented sample is very low which inevitably affects spatial quantification. Authors should consider removing that part or repeating with a better quality sample, maybe by following some of the recently published protocols as mentioned above (PMID: 37255850, PMID: 38767376).*

As we mentioned above regarding the suggestion to follow recently published protocols (PMID: 37255850, PMID: 38767376), we carefully evaluated these methods. As noted above, PMID: 37255850 utilized the Nanostring GeoMx DSP platform, which has significant limitations compared to Visium and Xenium, including lower resolution and reliance on predefined regions of interest rather than whole-transcriptome spatial mapping.

PMID: 38767376 employed an EDTA-based decalcification protocol, which we tested but found unsuitable for our approach. Specifically, we applied Visium spatial transcriptomics to EDTA-treated bone marrow and analyzed the data using standard filtering criteria: gene expression threshold = 1, gene detected in a minimum of 50 cells, and a minimum of 200 detected genes per cell. The results showed that only ~15% of cells/spots and <10% of genes passed these filters, leading to analysis failure (**new Extended Data Fig. 5k**). Please also refer to our response to the reviewer's first comment above, which confirms that EDTA-based decalcification leads to substantial mRNA degradation and is not suitable for spatial transcriptomics.

We agree that the overall tissue integrity in Fig. 2f appears lower compared to other samples. This is largely due to the technical challenges associated with processing newborn mouse bone marrow, which is highly fragile and prone to disruption during sectioning and slide preparation. Despite these inherent limitations, a substantial portion of the bone marrow tissue was successfully preserved and maintained sufficient structural integrity to enable meaningful spatial analysis in Fig. 2f. As shown in the corresponding data, we were still able to identify distinct cell types and assess their spatial relationships, including erythroid-C1q+ macrophage interactions.

• Figure 2h: those BM fragments do not look like intact bone marrow, which again unfortunately affects unbiased spatial quantification. This affects authors' conclusions on page 8. Could the authors show some IF images of EBIs in adult mouse bone marrow to support their findings?

Please refer to our response to the reviewer's first comment regarding EDTA methods and why cryosectioning is by far the best method to perform Visium spatial transcriptomic studies in adult mice.

As the reviewer suggested, we performed RNA-FISH combined with protein IF staining in adult mouse bone marrow and found that C1qa mRNA+ macrophage forms EBI structure with Ter119+ erythroid cells in adult mouse bone marrow (**new Extended Data Fig. 6c**).

• Figure 3: the authors state that "Erythroid cells surrounding C1Q+ macrophages could also be observed. However, this structure was not exclusive, as other cell types were also present in proximity". Given that the C1q+ centered EBIs are also visible, it could be that more cell types are integrated in the human setting like endothelial cells. Could the authors exclude that possibility based on their data?

We appreciate the reviewer's comment and the opportunity to clarify our statement. When we mention that erythroid cells surround C1Q+ macrophages, this does not imply that C1Q+ macrophage-centered EBIs are present in human tissue. As we demonstrated in **Figs. 3 and 4**, the localization of C1Q+ and other macrophages in human hematopoietic tissues appears random in relationship to the erythroid cells, without forming specific structures with the analyzed cell types. The only notable spatial relationship observed was between C1Q+ macrophages and endothelial cells, as shown in **Fig. 3l**.

Importantly, **Fig. 3l** also shows that endothelial cells do not exhibit a close spatial correlation with erythroid cells, demonstrating that endothelial cells are not a core component of human

EBIs. Our findings suggest that human EBI organization differs from that in mice, with no clear C1Q+ macrophage-centered structures observed. To avoid confusion, we changed "erythroid cells surrounding C1Q+ macrophages" to "erythroid cells adjacent to C1Q+ macrophages".

• Does the spatial profiling in Figure 1a reflect a single MDS patient? If so, would it be possible for authors to increase the n number here, as a significant variability is expected among human material even within the same disease entity (particularly since MDS is quite heterogeneous). If not, could authors show the additional patient profiles in supplemental?

In the original manuscript, **Fig. 5f** reflects a single MDS patient. However, to account for potential variability, we analyzed 7 MDS samples in the original manuscript using Visium, with the data presented in **Extended Data Fig. 11 (old Extended Data Fig. 6**). We also showed in the original manuscript that there is an increased correlation coefficient between erythroid progenitor cells and erythroid cells in post-therapy bone marrow compared to their pre-therapy counterparts (**Extended Data Fig. 11, and Fig. 5o**). This indicates that reduced physical connection among erythroid cells could be a dysplastic feature in MDS, which could be improved with therapy.

• The authors state that "Overall, these findings illustrate that human EBIs exhibit unique structures that are macrophage-independent and erythroid-centered". As mentioned above, if the existence of a multi-cellular EBI cannot be excluded, authors could rephrase their conclusion.

We appreciate the reviewer's suggestion and agree that while our findings strongly support a macrophage-independent, erythroid-centered organization in human EBIs, we cannot entirely exclude the presence of additional multicellular interactions.

To address this, we have refined our conclusion to more accurately reflect our observations. In the revised manuscript, we now state, "Overall, these findings illustrate that human EBIs exhibit a distinct organization from their murine counterparts, with erythroid precursors playing a central role. While our data do not support a macrophage-centered structure in human EBIs, we acknowledge that in addition to these erythroid-centered EBIs, there are also EBIs in humans where multiple cell types, including macrophages and other stromal cells, are intermixed."

• The authors state that "Xenium spatial studies showed continued C1q+ macrophage-centered EBIs as 3 well as markedly increased erythroid cells and their interactions (Extended Data Fig. 5h, i, Fig. 5e).". This statement is rather qualitative and the C1q+ centered EBIs are not immediately obvious, despite the evident expansion of erythroid cells. Could the authors perform quantification in multiple sections?

We thank the reviewer for this valuable suggestion. In response, we have now included a quantitative analysis of PHZ-treated versus control spleens (**new Extended Data Fig. 10j and 10k**). Both erythroid cells and macrophages were increased in number following PHZ treatment. However, when normalized to the total number of erythroid cells plus macrophages, the frequency of macrophage-centered EBIs was not significantly different between conditions (**new Extended Data Fig. 10j**). Interestingly, we observed a striking increase in erythroid-centered interactions that occurred independently of macrophages. This increase remained significant

even after normalization, suggesting that during hemolytic stress, erythroid cells may participate in or organize EBIs through alternative mechanisms (**new Extended Data Fig. 10k**). We have updated the text in the Results section to reflect these new findings and better support our conclusions.

• The manuscript suggests that ICAM4 plays a critical role in the formation of erythroid-centered EBIs in humans based on human bone marrow scRNA-seq dataset and functional validation on an iPSC-derived human bone marrow organoid model. To further strengthen this claim, it would be of interest to stain for the presence of ICAM4 protein on the healthy human bone marrow sample and its potential absence from the MDS human bone marrow samples.

We appreciate the reviewer's suggestion and have addressed this point by directly assessing ICAM4 protein expression in human bone marrow samples. To this end, we performed flow cytometry analysis, which provides a more objective and quantitative assessment compared to immunofluorescence staining, to compare ICAM4 expression levels between healthy human bone marrow and MDS bone marrow samples. The results showed that ICAM4 expression is significantly lower in MDS patients compared to healthy donors, further supporting its role in erythroid-centered EBI formation (**new Fig. 6g-h**). This conclusion is consistent with our finding that reduced physical connections among erythroid cells could be a dysplastic feature in MDS, further highlighting the disruption of normal EBI architecture in this disease.

• Given that the macrophage-erythroid cell interactions are not involved in the EBI formation in humans (through the ICAM4-integrin av axis), could the authors validate the relevant receptor-ligand pairs (shown in figure 6a) in situ and validate the corresponding cell proximity by IF imaging?

We appreciate the reviewer's suggestion and have performed additional in situ validation experiments to assess the receptor-ligand pairs shown in **Fig. 6a**.

In response, we attempted immunofluorescence staining for several candidate receptor-ligand pairs shown in Fig. 6a, including ICAM4. Unfortunately, we were unable to detect a specific and reproducible signal for ICAM4 or other binding partners by IF using currently available commercial antibodies, despite testing multiple protocols. This technical limitation highlights the inherent challenges of antibody-based detection in complex tissue environments and reinforces the value of spatial transcriptomic approaches for mapping cell-cell interactions in an unbiased manner.

However, we were able to detect strong ICAM4 expression on the surface of erythroid cells by flow cytometry (**new Fig. 6g-h**), consistent with prior reports and supporting its functional relevance in this context. Furthermore, our functional inhibition experiments (**Figs. 6b-6f**) suggest that ICAM4 plays a dominant role in erythroid-erythroid adhesion, potentially through alternative or compensatory mechanisms with many binding partners. We fully agree that identifying the precise binding partner(s) of ICAM4 in this context would be important, but we respectfully note that doing so would require extensive additional biochemical, proteomic, and genetic work. Given the scope and focus of the current study, we believe this represents a significant and independent line of investigation best pursued in future work.

We hope the reviewer appreciates that our current findings already provide a strong and multidimensional foundation for reevaluating human EBI organization and the central role of ICAM4.

Minor concerns:

• Figure 1g/h: Can authors explain how they can have negative values on the Y axis?

The values on the Y-axis represent relative expression scores, which were log-transformed and z-normalized before calculating their correlation. This transformation can result in negative values, as z-normalization centers the data around a mean of zero, with values above and below the mean appearing as positive and negative scores, respectively.

• Could the authors comment on why the identified so few hepatocytes on mouse fetal liver (Extended Figure 1b)?

We thank the reviewer's comment. It is well established that at embryonic day 14.5 (E14.5), the majority of cells in the mouse fetal liver are erythroid, as the fetal liver serves as the primary site of erythropoiesis during this developmental stage (PMID: 11844990, 18445648, 22337720, 39048071). As a result, hepatocytes are relatively sparse at this time point, which explains why we identified only a small number of hepatocytes in Extended Data Fig. 1b.

• Reference style is not consistent, as some references are lacking the journal's name and links (i.e. #26, 27).

We apologize for the inconsistency, which has been changed in the revised manuscript.

• Figure 1n: what is the distance between neighboring EBIs and how many EBIs can be found in a specific tissue volume?

We thank the reviewer for this insightful question. Based on our Xenium spatial transcriptomic analysis, we identified regions representing EBI structures and quantified their spatial distribution. Specifically, the distance between neighboring EBIs was determined by measuring the pixel distance between central macrophages of adjacent EBIs using ImageJ, then converting the pixel values into physical distances based on the actual dimensions of the tissue section on the Xenium slide.

The number of EBIs within a given tissue volume varies depending on the size of the section analyzed. However, it can be estimated by calculating the tissue area and dividing it by the average area occupied by a single EBI, as inferred from the measured inter-EBI distances. These quantifications are now presented in the revised manuscript as **Extended Data Fig. 4a**.

• Page 7: A day 8 postnatal mouse cannot be considered as "adult". Adult mouse are often 12 weeks old. Please consider revising the corresponding text.

We have revised this as “newborn”.

• Several Figure panels suffer from low rendering quality which makes them a bit difficult to read.

We have replaced lower-resolution images with high-resolution versions to ensure that all labels, markers, and data points are clearly visible. This problem could also be due to the PDF conversion process during submission. The reviewer could also directly download our figures from the submission website.

• On page 10 authors state “In addition, Xenium 9 spatial transcriptomic data also showed a close association of cells from the erythroid lineage 10 (Fig. 4h, Extended Data Fig. 4e, f).” Could the authors show the same analysis as in 4a and 4f?

Fig. 4f presents the distribution of different cell types based on Visium spatial transcriptomics data, whereas Fig. 4h is derived from Xenium data, which has a different resolution and detection approach. Due to these differences in spatial resolution and data structure, the same type of analysis cannot be directly applied to both datasets. However, each figure provides complementary insights into the spatial organization of erythroid lineage cells within the tissue.

• Could the authors also stain for C1q+ in the human bone marrow?

We have attempted C1q staining in human bone marrow samples. The staining pattern for C1q in human tissue appears diffuse. As such, it does not yield discrete cellular localization, and we did not include these data in the revised manuscript. Please see the representative image below demonstrating this diffuse staining pattern in the human bone marrow section. In this figure, CD235a again demonstrates the erythroid-centered EBI.

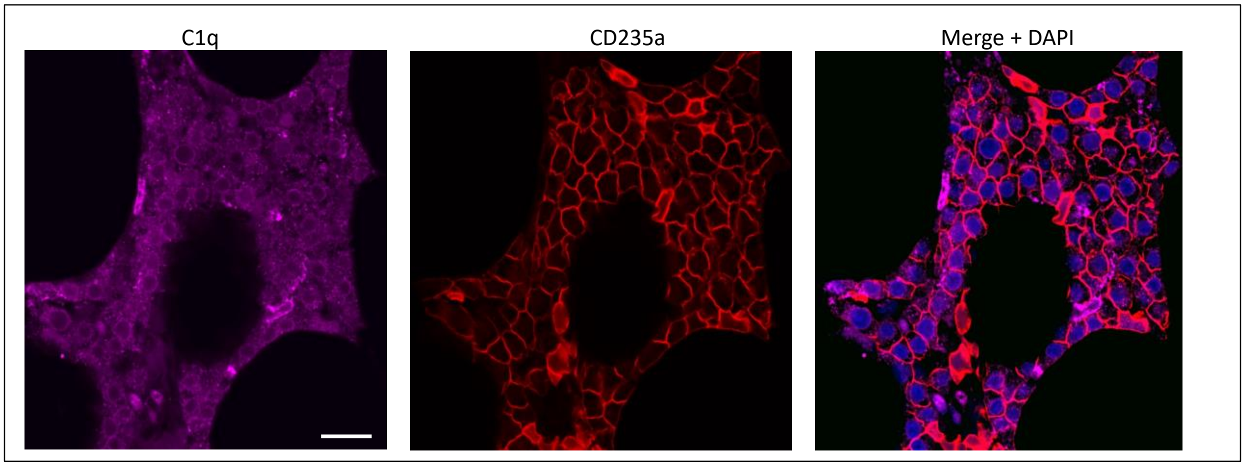

• Figure 5d: cluster annotations are missing. The C1q+ centered EBI is not immediately visible and spatial localization seems random? Could authors statistically test this possibility?

The cluster annotations are illustrated in the figure itself. We also illustrated the spatial relationships between various cell types using a heat map assay, demonstrating interactions

between two cell types based on Xenium spatial transcriptomic data (**new Extended Data Fig. 10g**), which provides statistical support for their interactions.

• Figure 6d: Could authors also show a larger area (zoom out) and several zoom in areas (in the supplemental).

We have included larger area pictures in the Extended Data Fig. 13.

Dear Dr. Faial,

It's our great pleasure to submit the revised manuscript titled "Spatial transcriptomic mapping reveals distinct erythroid niches between mice and humans." We are happy that Reviewers #2 and #3 found our previous revisions comprehensive and our study significantly enhanced. We also thank Reviewer #1 for their continued critical engagement with our work, which has allowed us to further refine and clarify our conceptual framework.

Point-by-point response:

Reviewer #1:

The authors have submitted a third version of this manuscript, in which they have now gone back on their main original claims, effectively confirming that they were not justified in the first place.

The first original claim, that human erythroblastic islands are 'erythroid centered' rather than macrophage centered, was dropped, and now the authors changed it into a claim that human erythroid cells are in 'self organized clusters' without any central cell. The authors write about their previous claim that they never meant it in the first place, that 'Rather, we used "centered" in a figurative sense to convey that erythroid cells appear to aggregate independently of macrophages, forming self-organized clusters'. It seems to me that the evidence for this last claim is also very weak. Simply finding cells in clusters does not make them 'self-organized'. There is no functional data to support a claim of organization. Close apposition between erythroid cells has been noted previously (as in my prior comments, with which the authors agree). These findings are therefore not new.

We appreciate this important point and agree that the terms "self-organized" or "self-assembled" were too strong, as they can imply an active process that is not directly supported by our current data. Our spatial transcriptomic, imaging, and organoid analyses support the presence of tight erythroid-erythroid spatial proximity and clustering, but they do not by themselves establish the basis for the clustering that arises in a "self-organized" manner. We have therefore removed the term "self-organized" or "self-assembled" throughout the manuscript and replaced it with purely descriptive language as "macrophage independent erythroid clusters" as appropriate.

With respect to novelty, the spatial erythroid-erythroid adjacency is not the only conceptual advance of the study. We show in the manuscript that the key contribution is not the mere presence of close erythroid apposition, but rather the systematic cross-species spatial comparison and functional experiments demonstrating that the dominant architecture of erythroid niches differs between mouse and human tissues, as assessed by multiple complementary in situ approaches and ex vivo assays. We also demonstrated the critical role of ICAM4 in macrophage-independent EBIs in humans. We believe this manuscript represents a meaningful conceptual and experimental advance, distinct from prior reports of erythroid cell proximity.

The second central claim originally made by the authors was that in human tissue there are no 'conventional' erythroblastic islands in which erythroblasts surround and are attached to macrophages. The authors now agree that such structures do exist, but claim they are present

at lower frequency than in mice. It is difficult to claim that this is a sufficiently novel or significant finding.

We appreciate this point and would like to further clarify. The prevailing model in hematology has long assumed that the macrophage-centered erythroblastic island (EBI) is the obligatory and deterministic unit of erythropoiesis across species. Our data reveal that in humans, this conventional EBI is not only less frequent but stochastic in its distribution. The significance of our finding lies in this divergent structural logic: while the conventional macrophage-centered EBI is critical for mouse erythropoiesis, human erythroid maturation demonstrates a much higher degree of spatial autonomy. The stochastic nature of human EBIs has been addressed in the previous revisions; we have further emphasized this in the revised manuscript.

This stochastic nature suggests that human erythropoiesis exhibits structural plasticity not observed in murine models. By identifying that a substantial portion of human erythroid maturation occurs independently of the conventional EBI context, we provide a necessary framework for investigating human-specific pathologies, such as myelodysplastic syndromes we showed in the manuscript, for which rigid murine EBI models have historically failed to yield translatable insights.

Reviewer #2

The authors have adequately addressed my comments and I have no further suggestions. The additional experiments, analyses and text edits have significantly enhanced the quality of the study.

We thank the reviewer for their acknowledgement of our revision.

Reviewer #3

In the revised manuscript, the authors have comprehensively addressed all prior scientific comments, including through the addition of new data and revisions to previous statements to clarify their interpretation. This reviewer has no further substantive suggestions; however, minor typographical and formal issues should be checked. For example, on page 4 (bottom), gene names should be formatted in italics.

We thank the reviewer for this careful reading. We have corrected the formatting of all gene names to italics throughout the manuscript, including the instance noted on page 4, and have performed a full check to ensure consistency.
